# Synthesis and Cycloaddition Reactions of 1-Azido-1,1,2,2-tetrafluoroethane

**DOI:** 10.1021/acs.joc.3c01346

**Published:** 2023-10-20

**Authors:** Elena Shaitanova, Václav Matoušek, Tadeáš Herentin, Martin Adamec, Robert Matyáš, Blanka Klepetářová, Petr Beier

**Affiliations:** † Institute of Organic Chemistry and Biochemistry of the Czech Academy of Sciences, Flemingovo Náměstí, 2, 166 10 Prague 6, Czech Republic; ‡ V. P. Kukhar Institute of Bioorganic Chemistry and Petrochemistry, The National Academy of Sciences of Ukraine, Academika Kukhara Str. 1, 02094 Kyiv, Ukraine; § CF Plus Chemicals, Karásek 1767/1, 621 00 Brno, Czech Republic; ∥ Institute of Energetic Materials, Faculty of Chemical Technology, University of Pardubice, Doubravice 41, 532 10 Pardubice, Czech Republic

## Abstract

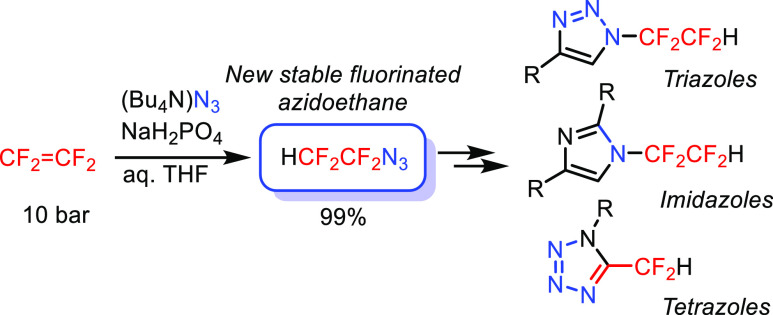

A new fluorinated azidoethane—1-azido-1,1,2,2-tetrafluoroethane—was prepared in quantitative yield by the addition of an azide anion to tetrafluoroethylene in a protic medium. The title azide was shown to be thermally stable and insensitive to impact. Copper(I)-catalyzed [3 + 2] cycloaddition with alkynes afforded 4-substituted *N*-tetrafluoroethyl-1,2,3-triazoles which underwent rhodium(II)-catalyzed transannulation with nitriles to novel *N*-tetrafluoroethylimidazoles or the reaction with triflic acid to enamido triflates. [3 + 2] Cycloaddition of the title azide with primary amines afforded novel 5-difluoromethyl tetrazoles.

## Introduction

Fluorinated organics are widely used in the development of pharmaceuticals and agrochemicals, as well as in diagnostics, polymer and material science, among other areas.^1−4^ Introduction of fluorine atoms or fluorine-containing groups to a molecule of a drug candidate is one of the most promising strategies in the development of modern pharmaceuticals. In the last 8–10 years, 20–50% of approved small-molecule drugs^5−8^ and 50–70% of agrochemicals^9,10^ contained a fluorine atom or atoms in the molecule of the active ingredient. Thus, the development of new procedures to obtain fluorine-containing small molecules is a continuing effort of high practical value.

**Figure 1:**
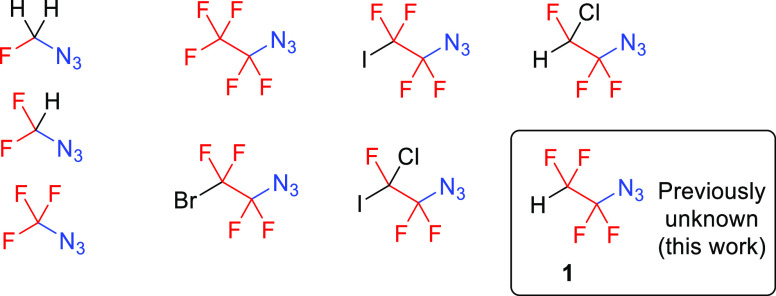
Known One- and Two-Carbon α-Fluorinated Azidoalkanes.

One group of recently introduced fluorinated reagents and building blocks are α-fluorinated azidoalkanes.^11^ Known one- and two-carbon members of this family are shown in Figure 1. Their unusually high stability (except azidofluoromethane) and synthetic utility have been demonstrated on copper(I)-catalyzed azide–alkyne cycloaddition (CuAAC), followed by transformations to novel fluorinated heterocycles,^12^ enamides,^13^ imidoyl halides,^14^ and ketenimines.^15^

Despite the importance of tetrafluoroethyl- and tetrafluoroethylene-containing compounds in synthesis and in various applications,^16^ azidotetrafluoroethane (**1**) is yet unreported. It was briefly mentioned in literature without experimental details and characterization^17^ and in situ generated by us also without characterization.^18^ Because of the established reactivity of polyfluorinated alkenes with nucleophiles, including the azide anion,^11,19^ our synthetic approach to **1** is based on the reaction of tetrafluoroethylene with a nucleophilic azide source and quench of the resulting fluorinated carbanion with a proton source.

## Results and Discussion

Tetrafluoroethylene is a multiton chemical used industrially mainly for the manufacture of poly(tetrafluoroethylene) (PTFE) and copolymers with other alkenes.^20^ Tetrafluoroethylene is an ideal two-carbon building block for incorporating fluorinated moieties such as tetrafluoroethyl, tetrafluoroethylene, and trifluorovinyl. However, it is a suspected carcinogen, unstable when exposed to radicals, and prone to exothermic polymerization. These properties require caution when handling tetrafluoroethylene.

**Scheme 1:**
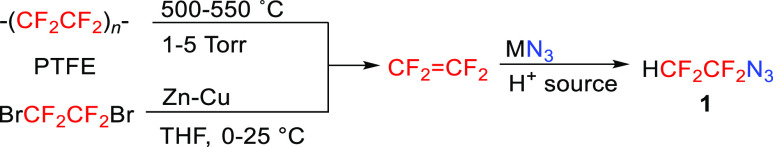
Synthetic Approaches to the Synthesis of **1** via Tetrafluoroethylene

On an industrial scale, tetrafluoroethylene is formed via the dimerization of difluorocarbene formed from HCF_2_Cl, but on a laboratory scale, the preferred methods are the reduction of 1,2-dihalotetrafluoroethane with zinc,^21^ vacuum pyrolysis of PTFE,^22^ decarboxylation of sodium pentafluoropropionate^20^ or from the Ruppert–Prakash reagent (TMSCF_3_) and sodium iodide.^23,24^ We used the first two methods to access tetrafluoroethylene and, subsequently, form the target novel azide (Scheme 1). The results of optimization employing various azide and proton sources under different conditions are summarized in Table 1.

**Table 1:** Optimization of the Synthesis of **1**^a^

entry	MN_3_	H^+^ source	solvent	temp. (°C)	yield of **1** (%)^b^
1	HN_3_	HN_3_	THF	rt	0
2^c^	NaN_3_	(Bu_4_N)HSO_4_	EtOH	60	0
3^d^	NaN_3_	(Bu_4_N)HSO_4_	THF	35	32
4	(Et_3_NH)N_3_	Et_3_NHN_3_	THF	rt	0
5^e^	(Et_3_NH)N_3_	NaH_2_PO_4_	THF	rt	80
6^e^	(Bu_4_N)N_3_	(NH_4_)_2_SO_4_	THF	60	50
7^e^	(Bu_4_N)N_3_	NaH_2_PO_4_	THF	rt	100
8^e^	(Bu_4_N)N_3_	NaH_2_PO_4_	NMP	rt	75

a Reaction conditions: CF_2_=CF_2_ (10 bar, excess) prepared by depolymerization of PTFE; MN_3_ (52 mmol), H^+^ source (104 mmol), THF (60 mL), 2 h.

b ^19^F NMR yield.

c EtOH (5 mL) + H_2_O (2 mL).

d CF_2_=CF_2_ (1 bar, excess) prepared from BrCF_2_CF_2_Br and Zn–Cu; NaN_3_ (50 mmol), (Bu_4_N)HSO_4_ (50 mmol), THF (50 mL), H_2_O (43 mL), 14 days.

e With added water (4 equiv).

**Scheme 2:**
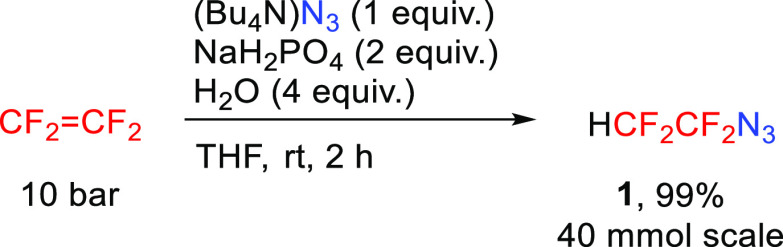
Optimized Preparative Synthesis of Azidotetrafluoroethane **1**

Performing the reaction in an autoclave with tetrafluoroethylene (ca. 10 bar) obtained by depolymerization of PTFE and the use of hydrazoic acid^25^ did not lead to any product (entry 1). The same result was observed with sodium azide and tetrabutylammonium hydrogensulfate in aqueous ethanol under elevated temperature (entry 2). However, the use of aqueous THF and extremely long reaction time (14 days) afforded a low NMR yield of **1** when only 1 bar of tetrafluoroethylene formed by the reduction of BrCF_2_CF_2_Br was used (entry 3). Returning to the use of pressurized tetrafluoroethylene in an autoclave and the use of triethylammonium azide or tetrabutylammonium azide afforded good product yields using a suitable proton source (dihydrogen phosphate or ammonium sulfate as pH buffers, entries 5 and 6). Finally, the highest yield of **1** was obtained by using tetrabutylammonium azide and sodium dihydrogen phosphate in wet THF (entry 7). The product **1** was isolated in quantitative yield by codistillation with THF (Scheme 2). A pure sample of **1** (bp 30–32 °C) was obtained by distillation from the high boiling solvent NMP (experiment Table 1, entry 8).

Although other heavily fluorinated small organic azides were proven to be unusually stable and are even commercially available, it was necessary to establish the stability limits of **1** for safe use in synthesis. An indicative thermal stability test was performed by sealing a THF + CDCl_3_ solution of **1** in a high-pressure NMR tube. After heating the tube to 150 °C for 8 h, no decomposition was observed by ^19^F NMR (see the Supporting Information for details). The Koenen test (sensitivity to heat) and fall-hammer test (sensitivity to impact) of a solution of **1** in THF (0.5 M) were both negative (see Supporting Information for details). We therefore conclude that azide **1** is safe to use on a laboratory scale in solution under ambient or moderately harsh conditions.

**Scheme 3:**
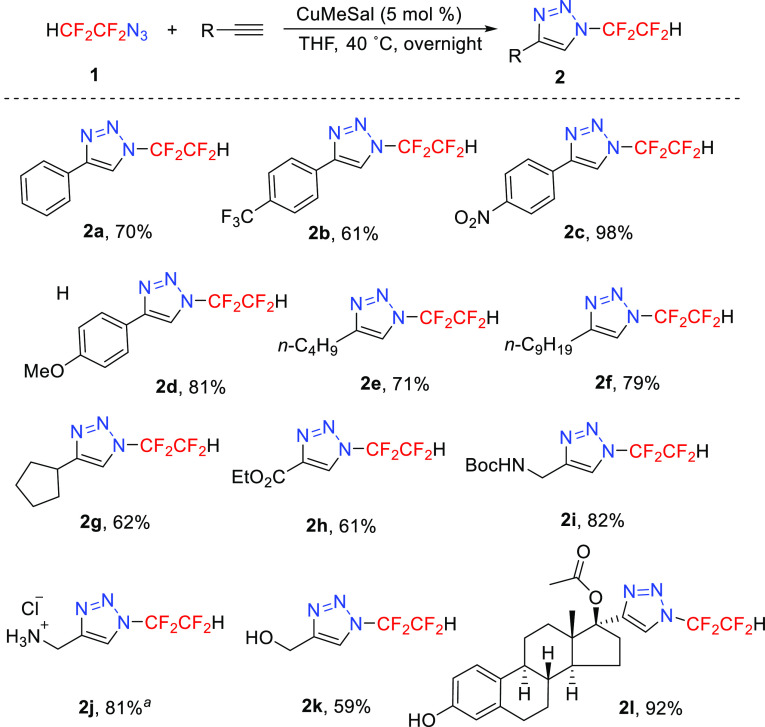
Synthesis of 4-Substituted *N*-Tetrafluoroethyl Triazoles Prepared from **2i** using HCl (6 equiv), Et_2_O, 0 °C to rt, 18 h.

After developing an efficient and scalable synthesis of azidotetrafluoroethane **1**, we evaluated its reactivity with a terminal alkyne using a CuAAC reaction. The application of conditions previously used in triazole formation from other azido(per)fluoroalkanes developed by us,^26^ namely, THF-soluble catalyst copper(I) 3-methylsalicylate (CuMeSal), afforded exclusively 1,4-disubstituted-1,2,3-triazoles in good to high yields. The reaction was not limited to aryl (electron-rich, neutral, or electron-poor) acetylenes; other competent substrates were alkyl and cycloalkyl acetylenes, containing various functional groups (ester, hydroxyl, protected amine), and even a functionalized steroid derivative (Scheme 3). The obtained triazoles are stable solids and were easily purified by column chromatography on silica gel or by crystallization. The triazole core and *N*-tetrafluoroethyl substitution of compound **2i** survived acidic deprotection, affording triazole **2j** with an amino function as a potentially useful building block.

**Scheme 4:**
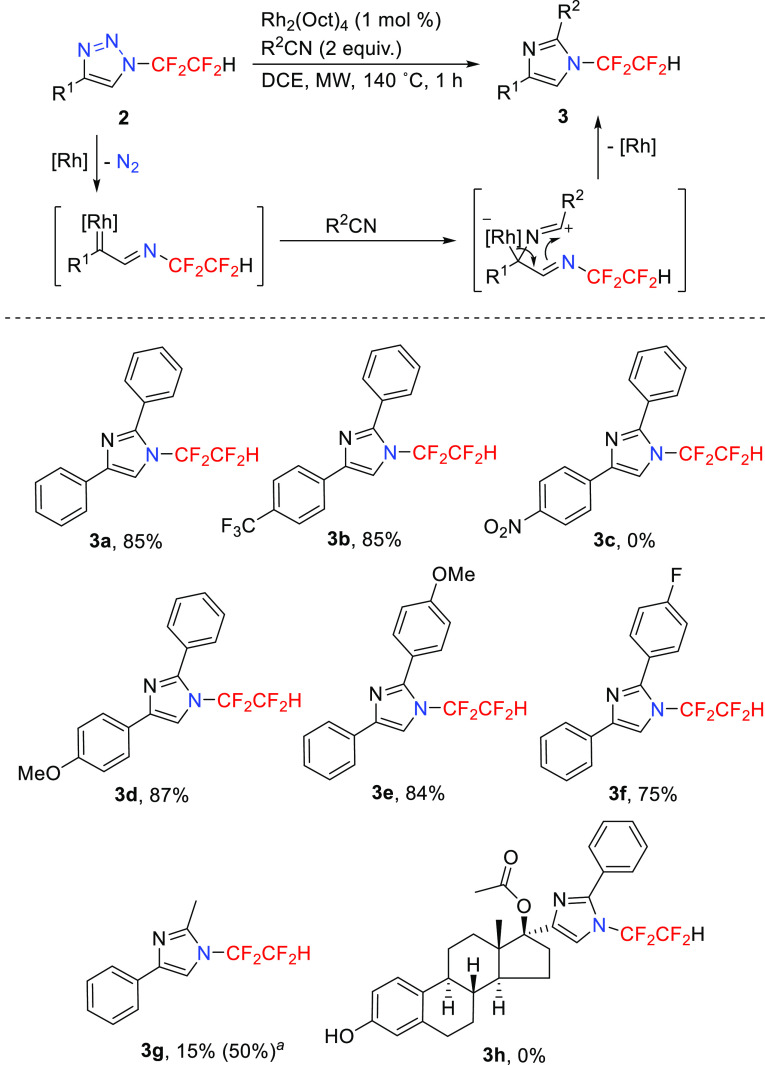
Rhodium(II)-Catalyzed Transannulation of Triazoles **2** with Nitriles Using MeCN (10 equiv) and 3 h reaction time. ^19^F NMR yield in parentheses.

Triazoles **2** were employed in a rhodium(II)-catalyzed transannulation reaction^12^ with nitriles to afford *N*-tetrafluoroethyl-substituted imidazoles. Among nitrogen heterocycles used in medicinal chemistry, imidazoles are privileged scaffolds^27,28^ and *N*-CF_2_CF_2_H-substituted imidazoles are rare.^29,30^ The method outlined above was applied to newly synthesized triazoles **2**, and the corresponding imidazoles **3** were obtained (Scheme 4). Under microwave heating, the transannulation reaction proceeded well with triazoles bearing aryl groups in position 4 (not alkyl groups), including a neutral phenyl group and moderately electron-poor and electron-rich aryl groups. The triazole with strongly electron-acceptor aryl group in position 4 was unreactive (**3c**). The reaction proceeded well with benzonitrile and its derivatives; however, with acetonitrile, the reaction was less efficient. Triazole **2l** having a steroidal structure was found to be unreactive.

**Scheme 5:**
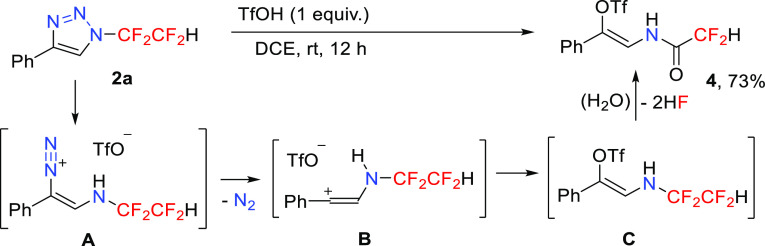
Synthesis of β-Enamido Triflate **4**

To further demonstrate the synthetic potential of the synthesized triazole products, we investigated another denitrogenative transformation, this time mediated by a strong Brønsted acid. Previously, we have shown that *N*-fluoroalkylated 1,2,3-triazoles in the presence of triflic or fluorosulfonic acids afford β-enamido triflates or fluorosulfonates, respectively, which are stereoselectively functionalized *N*-alkenyl compounds useful in enamide synthesis.^13^ Indeed, the reaction of triazole **2a** with an equimolar amount of triflic acid provided unstable enamine **C** via diazonium salt **A** and vinyl cation **B**. Intermediate **C** hydrolyzed to the corresponding β-enamido triflate **4** in a good yield (Scheme 5).

**Scheme 6:**
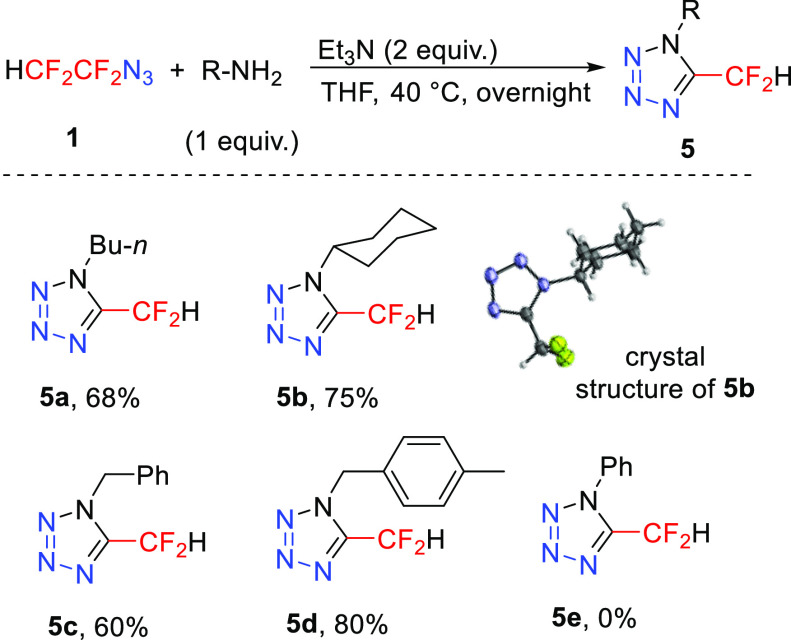
Reaction of Azidotetrafluoroethane with Primary Amines

Primary amines react with α,α-difluorinated azido alkanes to afford tetrazoles,^31^ important polyazaheterocycles^32^ displaying various bioactive properties.^33^ Because tetrazoles bearing the difluoromethyl moiety are unknown, we investigated the reaction of azide **1** with primary amines. Optimization of the reaction conditions revealed that *n*-butylamine reacted with **1** under mild conditions and full conversion of **5a** was reached in 12 h at ambient temperature or in 2 h at 40 °C (Table 2). Amide side product **6a** comes from the substitution of the azido group with amine and hydrolysis. Two equivalents of the triethylamine base are necessary for the neutralization of the 2 equiv of HF formed, and the reaction is water-tolerant. A small scope study revealed that alkyl-, cycloalkyl-, and benzyl-type primary amines were competent substrates and the corresponding tetrazoles **5** formed in good yields (Scheme 6). The structure of compound **5b** was confirmed by X-ray analysis. Aniline, on the other hand, was an ineffective amine in the preparation of the tetrazoles, most likely owing to its low nucleophilicity. Small amounts of side products **6** were separated from **5** by column chromatography or by basic hydrolysis of **6**. Tetrazoles **5** are highly resistant to basic hydrolysis and prolonged heating of the reaction mixture with NaOH (1 M) caused the hydrolysis of amide **6** but left **5** unchanged.

**Table 2:**
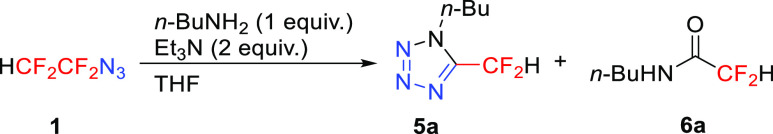
Optimization of Reaction Conditions in the Formation of Tetrazole **5a**

entry	temp. (°C)	time (h)	yield (%)^a^	
			**5a**	**6a**
1	rt	1	49	5
2	rt	2	85	5
3	rt	12	95	5
4	40	1	70	3
5	40	2	92	8
6^b^	rt	1	32	7
7^b^	rt	2	72	8
8^b^	rt	12	92	8

a ^19^F NMR yield.

b With added water (1 equiv).

**Scheme 7:**
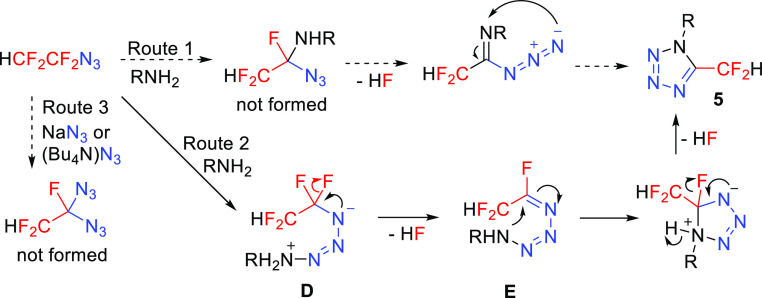
Proposed Mechanism for the Formation of Tetrazoles **5**

A single report describing the formation of 5-fluoroalkyl-substituted tetrazoles suggested the mechanism proceeding by the substitution of the α-fluorine atom of the azide with nitrogen nucleophiles (Scheme 7, route 1).^31^ However, this process is highly unlikely because halogen substitution on quaternary carbon atoms is difficult. We suggest that nucleophilic nitrogen of the primary amine attacks the terminal nitrogen of the azido moiety^34^ to form intermediate **D**, which eliminates HF to produce intermediate **E**, whose cyclization leads to tetrazole **5** (Scheme 7, route 2). Both HF eliminations are facilitated by the presence of a base. Additionally, a control experiment of utilizing the highly nucleophilic azide anion NaN_3_ or (Bu_4_N)N_3_ revealed no reactivity with **1** in a substitution fashion even under prolonged heating to 80 °C (Scheme 7, route 3) which makes route 1 unlikely.

In conclusion, a method for the preparation of the new 1-azido-1,1,2,2-tetrafluoroethane (**1**) based on the addition of the azide anion to tetrafluoroethylene in a protic environment is reported. The title azide is prepared on a multigram scale in quantitative yield and demonstrated a good thermal stability and a nonexplosive character. Azide **1** undergoes [3 + 2] cycloaddition with terminal alkynes catalyzed by Cu(I) salts to 4-substituted *N*-tetrafluoroethyl-1,2,3-triazoles. Subsequent rhodium(II)-catalyzed transannulation with nitriles provides the corresponding *N*-tetrafluoroethyl-containing imidazoles. Acid-mediated denitrogenation of triazole **2a** affords β-enamido triflate **4**. 5-Substituted *N*-tetrafluoroethyltetrazoles **5** are prepared by the reaction of azide **1** with primary amines. The reaction proceeds by the attack of the nucleophilic nitrogen of primary amines on the terminal nitrogen of the azide moiety, followed by HF elimination and, finally, cyclization.

## Experimental Section

### General Information

All reactions were carried out in oven-dried vessels under a dry N_2_ atmosphere. All chemicals were obtained from commercial sources and used as received. (Bu_4_N)N_3_ was prepared using a published procedure.^35^ THF was freshly distilled over Na/benzophenone prior to use. CDCl_3_ and DMF were dried using molecular sieves (3 and 4 Å, respectively). ^1^H, ^13^C, and ^19^F NMR spectra were measured at ambient temperature using 5 mm diameter NMR tubes. The chemical shift values (δ) are reported in parts per million relative to internal Me_4_Si (0 ppm for ^1^H and ^13^C NMR) or residual solvents and internal CFCl_3_ (0 ppm for ^19^F NMR).

### 1-Azido-1,1,2,2-tetrafluoroethane (**1**)

Tetrafluoroethylene was obtained by the pyrolysis of PTFE shavings (25 g) in a quartz tube (46 mm diameter) at 500–550 °C in an electric furnace for 3 h under vacuum (1–5 Torr) and condensation in a trap immersed in liquid nitrogen. **Caution!** Tetrafluoroethylene is toxic and its mixtures with air are explosive. To a 300 mL-stainless autoclave equipped with a glass insert and a magnetic stirring bar, the following was added: (Bu_4_N)N_3_ (11.5 g, 40 mmol, 1 equiv), NaH_2_PO_4_ (9.7 g, 80 mmol, 2 equiv), H_2_O (2.9 mL, 160 mmol, 4 equiv), and THF (60 mL). The autoclave was cooled with liquid nitrogen, and tetrafluoroethylene (∼10 g, 100 mmol, 3.5 equiv) was condensed to it from the trap. The autoclave was sealed, the cooling bath was removed, and the mixture was allowed to warm to room temperature and stirred overnight (10 bar). Then, the autoclave was cooled with an ice bath, the gaseous products were vented off, and citric acid (5.76 g, 40 mmol, 1 equiv) and Na_2_SO_4_ (10 g, 70 mmol) were added. The reaction mixture was filtered and distilled at ambient pressure to afford **1** (oil bath temperature up to 90 °C, heating mantle) together with THF to a cooled (−78 °C) receiving flask containing PhCF_3_ as an internal standard. The product was obtained as a THF solution. **Caution!** Excessive heating of the solution of **1** or neat **1** can cause explosion. Yield: 99% (57 mL of 0.7 M THF solution, 39.86 mmol). An analytically pure sample was obtained by repeating the procedure using NMP instead of THF and redistillation under ambient pressure giving a colorless liquid, bp 30–32 °C, IR (CDCl_3_ film): 2991, 2163, 1292, 1273, 1236, 1130, 1065 cm^–1^; ^1^H NMR (400 MHz, CDCl_3_): δ 5.79 (tt, *J* = 53.0 Hz, 2.3 Hz); ^13^C{^1^H} NMR (101 MHz, CDCl_3_): δ 115.4 (tt, ^1^*J*_C–F_ = 267.4, Hz, ^2^*J*_C–F_ = 28.5 Hz, CF_2_), 108.2 (tt, ^1^*J*_C–F_ = 253.1 Hz, ^2^*J*_C–F_ = 41.8 Hz, CF_2_H); ^19^F NMR (376 MHz, CDCl_3_): δ −95.0 (d, *J* = 6.0 Hz, 2F), −137.7 (dt, *J* = 53.0, 6.0 Hz, 2F); HRMS (EI^+^): *m*/*z* calcd for C_2_HF_4_N_3_ [M]^+^ 143.0101; found, 143.0107.

### General Procedure for the Synthesis of Triazoles **2**

Terminal alkyne (1 mmol) was added to a THF solution of azide **1** (1.2 mmol, 3 mL) in a screw-cap tube. CuMeSal (11 mg, 0.05 mmol, 5 mol %) was added, and the tube was closed. The reaction mixture was stirred overnight at 40 °C (aluminum heating block and heating mantle). The solvent was evaporated under reduced pressure, and the crude product was purified by column chromatography on silica gel.

#### 4-Phenyl-1-(1,1,2,2-tetrafluoroethyl)-1*H*-1,2,3-triazole (**2a**)

Purified by column chromatography (cyclohexane/EtOAc, 6:1) and obtained as white crystals. Yield: 171 mg, 70%, mp 62 °C. NMR spectra corresponded to the literature.^36^

#### 1-(1,1,2,2-Tetrafluoroethyl)-4-(4-(trifluoromethyl)-phenyl)-1*H*-1,2,3-triazole (**2b**)

Purified by column chromatography (cyclohexane/EtOAc, 6:1) and obtained as white crystals. Yield: 191 mg, 61%, mp 76 °C; ^1^H NMR (500 MHz, CDCl_3_): δ 8.26 (d, *J* = 0.8 Hz, 1H), 7.98 (m, 2H), 7.75–7.68 (m, 2H), 6.64 (tt, *J* = 52.3, 4.4 Hz, 1H); ^13^C{^1^H} NMR (126 MHz, CDCl_3_): δ 147.3, 132.3, 131.3 (q, *J*_C–F_ = 32.5 Hz), 126.4, 126.2 (q, *J*_C–F_ = 4.1 Hz), 123.9 (q, *J*_C_–_F_ = 271.5 Hz), 112.3 (tt, *J*_C–F_ = 267.5, 29.0 Hz, CF_2_), 107.7 (tt, *J*_C–F_ = 254.6, 35.7 Hz, CF_2_H); ^19^F NMR (376 MHz, CDCl_3_): δ −62.9 (s, 1F), −98.9 (td, *J* = 7.5, 4.5 Hz, 2F), −137.2 (dt, *J* = 52.5, 7.5 Hz, 2F); HRMS (APCI^+^): *m*/*z* calcd for C_11_H_7_F_7_N_3_ [M]^+^ 314.0522; found, 314.0522.

#### 4-(4-Nitrophenyl)-1-(1,1,2,2-tetrafluoroethyl)-1*H*-1,2,3-triazole (**2c**)

Purified by column chromatography (cyclohexane/EtOAc, 6:1) and obtained as white crystals. Yield: 284 mg, 98%, mp 116 °C; ^1^H NMR (400 MHz, CDCl_3_): δ 8.40–8.35 (m, 3H), 8.13–8.06 (m, 2H), 6.68 (tt, *J* = 52.3, 4.4 Hz, 1H); ^13^C{^1^H} NMR (126 MHz, CDCl_3_): δ 148.3, 146.5, 134.9, 126.9, 124.6, 119.1, 112.3 (tt, *J*_C–F_ = 265.0, 31.0 Hz, CF_2_), 107.7 (tt, *J*_C–F_ = 255.0, 36.0 Hz, CF_2_H); ^19^F NMR (376 MHz, CDCl_3_): δ −98.9 (td, *J* = 7.3, 4.3 Hz, 2F), −137.1 (dt, *J* = 52.5, 7.4 Hz, 2F); HRMS (EI^+^): *m*/*z* calcd for C_10_H_6_F_4_N_4_O_2_ [M]^+^ 290.0421; found, 290.0419.

#### 4-(4-Methoxyphenyl)-1-(1,1,2,2-tetrafluoroethyl)-1*H*-1,2,3-triazole (**2d**)^18^

Purified by column chromatography (cyclohexane/EtOAc, 8:1) and obtained as a white solid. Yield: 223 mg, 81%, mp 63 °C; ^1^H NMR (400 MHz, CDCl_3_): δ 8.11 (d, *J* = 0.8 Hz, 1H), 7.88–7.77 (m, 2H), 7.07–6.97 (m, 2H), 6.68 (tt, *J* = 52.4, 4.6 Hz, 1H), 3.88 (s, 3H); ^13^C{^1^H} NMR (101 MHz, CDCl_3_): δ 160.4, 148.5, 127.5, 121.3, 116.4, 114.5, 107.7 (tt, *J*_C–F_ = 254.5, 36.0 Hz, CF_2_H), 109.5 (tt, *J*_C–F_ = 264.0, 28.9 Hz, CF_2_), 55.4; ^19^F NMR (376 MHz, CDCl_3_): δ −98.9 (td, *J* = 7.8, 4.6 Hz, 2F), −137.3 (dt, *J* = 52.6, 7.9 Hz, 2F); HRMS (ESI^+^): *m*/*z* calcd for C_11_H_10_F_4_N_3_O [M + H]^+^ 276.07600; found, 276.0756.

#### 4-Butyl-1-(1,1,2,2-tetrafluoroethyl)-1*H*-1,2,3-triazole (**2e**)

Purified by column chromatography (cyclohexane/EtOAc, 3:1) and obtained as a colorless oil. Yield: 160 mg, 71%; ^1^H NMR (400 MHz, CDCl_3_): δ 7.72 (s, 1H), 6.62 (tt, *J* = 52.5, 4.6 Hz, 1H), 2.93–2.69 (m, 2H), 1.78–1.65 (m, 2H), 1.50–1.36 (m, 2H), 0.96 (t, *J* = 7.3 Hz, 3H); ^13^C{^1^H} NMR (101 MHz, CDCl_3_): δ 149.3, 118.6, 112.1 (tt, *J*_C–F_ = 264.0, 30.0 Hz, CF_2_), 107.7 (tt, *J*_C_–_F_ 253.5, 35.4 Hz, CF_2_H), 31.0, 24.9, 22.2, 13.7; ^19^F NMR (376 MHz, CDCl_3_): δ −98.9 (td, *J* = 7.8, 4.7 Hz, 2F), −137.5 (dt, *J* = 52.5, 7.8 Hz, 2F); HRMS (ESI^+^): *m*/*z* calcd for C_8_H_12_F_4_N_3_ [M + H]^+^ 226.0962; found, 226.0962.

#### 4-Nonyl-1-(1,1,2,2-tetrafluoroethyl)-1*H*-1,2,3-triazole (**2f**)

Purified by column chromatography (cyclohexane/EtOAc, 9:1) and obtained as a colorless oil. Yield: 133 mg, 79%; ^1^H NMR (400 MHz, CDCl_3_): δ 7.72 (p, *J* = 0.8 Hz, 1H), 6.63 (tt, *J* = 52.4, 4.7 Hz, 1H), 2.79 (m, 2H), 1.73 (m, 2H), 1.34 (m, 12H), 0.90 (m, 3H); ^13^C{^1^H} NMR (101 MHz, CDCl_3_): δ 149.4, 118.6, 112.0 (tt, *J*_C–F_ = 265.2, 30.0 Hz, CF_2_), 107.7 (tt, *J* = 253.5, 35.4 Hz, CF_2_H), 31.8, 29.4, 29.3, 29.2, 29.1, 29.0, 25.3, 22.6, 14.0; ^19^F NMR (376 MHz, CDCl_3_): δ −98.9 (td, *J* = 7.8, 4.8 Hz, 2F), −137.5 (dt, *J* = 52.6, 7.7 Hz, 2F); HRMS (ESI^+^): *m*/*z* calcd for C_13_H_22_F_4_N_3_ [M + H]^+^ 296.1745; found, 296.1744.

#### 4-Cyclopentyl-1-(1,1,2,2-tetrafluoroethyl)-1*H*-1,2,3-triazole (**2g**)

Purified by column chromatography (cyclohexane/EtOAc, 8:1) and obtained as a colorless oil. Yield: 147 mg, 62%; ^1^H NMR (400 MHz, CDCl_3_): δ 7.70 (q, *J* = 0.9 Hz, 1H), 6.63 (tt, *J* = 52.5, 4.8 Hz, 1H), 3.26 (dtd, *J* = 15.7, 7.8, 3.9 Hz, 1H), 2.17 (m, 2H), 1.68–1.74 (m, 6H); ^13^C{^1^H} NMR (101 MHz, CDCl_3_): δ 153.6, 117.7, 112.0 (tt, *J*_C–F_ = 265.2, 29.0 Hz, CF_2_), 107.7 (tt, *J* = 253.7, 35.4 Hz, CF_2_H), 36.4, 33.0, 25.1; ^19^F NMR (376 MHz, CDCl_3_): δ −98.8 (td, *J* = 7.9, 4.8 Hz, 2F), −137.5 (dt, *J* = 52.4, 7.9 Hz, 2F); HRMS (ESI^+^): *m*/*z* calcd for C_9_H_11_F_4_N_3_ [M + H]^+^ 238.0965; found, 238.0962.

#### Ethyl 1-(1,1,2,2-Tetrafluoroethyl)-1*H*-1,2,3-triazole-4-carboxylate (**2h**)

Purified by column chromatography (cyclohexane/EtOAc, 3:7) and obtained as a white solid. Yield: 147 mg, 61%; ^1^H NMR (500 MHz, CDCl_3_): δ 8.50 (s, 1H), 6.60 (tt, *J* = 52.3, 4.2 Hz, 1H), 4.47 (q, *J* = 7.1 Hz, 2H), 1.43 (t, *J* = 7.1 Hz, 3H); ^13^C{^1^H} NMR (101 MHz, CDCl_3_): δ 159.3, 141.0, 126.1, 112.1 (tt, *J* = 268.0, 29.3 Hz), 107.5 (tt, *J* = 254.3, 35.7 Hz), 62.0, 14.2; ^19^F NMR (471 MHz, CDCl_3_): δ −99.2 (dd, *J* = 11.0, 6.6 Hz, 2F), −137.2 (dt, *J* = 52.4, 7.0 Hz, 2F); HRMS (ESI^+^): calcd *m*/*z* for C_7_H_8_F_4_N_3_O_2_ [M]^+^ 242.0547; found, 242.0549.

#### *tert*-Butyl ((1-(1,1,2,2-Tetrafluoroethyl)-1*H*-1,2,3-triazol-4-yl)methyl)carbamate (**2i**)

Purified by column chromatography (cyclohexane/EtOAc, 3:1) and obtained as a colorless oil. Yield: 244 mg, 82%; ^1^H NMR (300 MHz, CDCl_3_): δ 7.99 (s, 1H), 6.59 (tt, *J* = 52.4, 4.5 Hz, 1H), 5.12 (s, 1H), 4.47 (d, *J* = 6.1 Hz, 2H), 1.45 (s, 9H); ^13^C{^1^H} NMR (126 MHz, CDCl_3_): δ 155.8, 146.6, 120.7, 112.04 (tt, *J* = 264.5, 29 Hz), 107.6 (tt, *J* = 253.8, 35.6 Hz), 80.2, 77.2, 35.8, 28.21; ^19^F NMR (282 MHz, CDCl_3_): δ −98.9 (dd, *J* = 11.8, 7.2 Hz, 2F), −137.3 (dt, *J* = 52.4, 7.5 Hz, 2F); HRMS (ESI^+^): calcd *m*/*z* for C_10_H_14_F_4_N_4_NaO_2_ [M]^+^ 321.0945; found, 321.0945.

#### (3-(1,1,2,2-Tetrafluoroethyl)-1*H*-triazol-4-yl)methanammonium Chloride (**2j**)

To a 20 mL round-bottom flask containing **2i** (0.24 g, 0.82 mmol, 1 equiv) was added a cold solution of HCl in Et_2_O (0.81 mL of 6 M, 4.88 mmol, 6 equiv). The reaction mixture was stirred at 0 °C for 2 h and then at rt for 16 h. The formed solid was filtered off on a glass frit, washed with cold Et_2_O (−30 °C, 2 × 10 mL), and dried under reduced pressure. The product was obtained as a white solid. Yield: 163 mg, 81%, mp 145 °C; ^1^H NMR (500 MHz, DMSO-*d*_6_): δ 9.04 (s, 1H), 8.86 (s, 2H), 7.48 (tt, *J* = 50.9, 3.6 Hz, 1H), 4.23 (s, *J* = 24.7 Hz, 2H); ^13^C{^1^H} NMR (126 MHz, DMSO-*d*_6_): δ 142.6, 124.4, 112.5 (tt, *J*_C–F_ = 266.0, 28.5 Hz, CF_2_), 108.4 (tt, *J* = 252.5, 36.5 Hz, CF_2_H), 33.9 (s); ^19^F NMR (471 MHz, DMSO-*d*_6_): δ −97.7 (dd, *J* = 10.1, 6.1 Hz, 2F), −137.6 (dt, *J* = 50.9, 6.5 Hz, 2F); HRMS (ESI^+^) calcd *m*/*z* for C_5_H_7_F_4_N_4_ [M]^+^ 199.0601; found, 199.0603.

#### (1-(1,1,2,2-Tetrafluoroethyl)-1*H*-1,2,3-triazol-4-yl)methanol (**2k**)

Purified by column chromatography (cyclohexane/EtOAc, 4:1) and obtained as a pale-yellow oil. Yield: 117 mg, 59%; ^1^H NMR (300 MHz, CDCl_3_): δ 8.03 (m, 1H), 6.57 (tt, *J* = 52.4, 4.5 Hz, 1H), 4.83 (s, 2H), 4.13(t, 1H); ^13^C{^1^H} NMR (101 MHz, CDCl_3_): δ 148.7, 120.4, 112.1 (tt, *J* = 264.0, 29.0 Hz), 107.6 (tt, *J* = 253.7, 35.9 Hz), 55.6; ^19^F NMR (282 MHz, CDCl_3_): δ −99.2 (td, *J* = 7.1, 4.1 Hz, 2F), −137.5 (dt, *J* = 52.4, 7.5 Hz, 2F); HRMS (ESI^+^) calcd *m*/*z* for C_5_H_6_F_4_N_3_O [M]^+^ 200.0441; found, 200.0442.

#### (8*R*,9*S*,13*S*,14*S*,17*S*)-3-Hydroxy-13-methyl-17-(1-(1,1,2,2-tetrafluoroethyl)-1*H*-1,2,3-triazol-4-yl)-7,8,9,11,12,13,14, 15,16,17-decahydro-6*H*-cyclopenta[*a*]phenanthren-17-yl Acetate (**2l**)

Purified by column chromatography (cyclohexane/EtOAc, 9:1) and obtained as a white solid. Yield: 442 mg, 92%, mp ∼200 °C (decomp.); ^1^H NMR (401 MHz, CDCl_3_): δ 7.74 (d, *J* = 0.9 Hz, 1H), 7.07 (dd, *J* = 8.5, 1.1 Hz, 1H), 6.58 (m, 2H), 6.60 (tt, 1H, *J* = 53.4, 4.4 Hz, 1H), 4.86 (s, 1H), 3.05 (ddd, *J* = 15.4, 9.7, 5.9 Hz, 1H), 2.93–2.74 (m, 2H), 2.37–2.16 (m, 2H), 2.10 (s, 3H), 2.07–1.74 (m, 4H), 1.69–1.30 (m, 5H), 1.12 (s, 3H), 0.70 (td, *J* = 12.8, 4.2 Hz, 1H); ^13^C{^1^H} NMR (101 MHz, CDCl_3_): δ 170.2, 153.5, 151, 138.1, 132.1, 126.4, 119.5, 115.3, 112.1 (tt, *J*_C–F_ = 266.0, 28.7 Hz, CF_2_), 107.6 (tt, *J* = 253.5, 35.6 Hz, CF_2_H), 87.9, 47.9, 46.7, 43.2, 39.1, 35.7, 33.2, 29.6, 27.3, 26.0, 24.0, 21.6, 14.8; ^19^F NMR (376 MHz, CDCl_3_): δ −98.0 to −99.6 (m, 2F), −137.2 (dt, *J* = 52.5, 7.8 Hz, 2F); HRMS (ESI^+^): *m*/*z* calcd for C_24_H_27_F_4_N_3_NaO_3_ [M + Na]^+^ 504.1882; found, 504.1881.

### General Procedure for the Synthesis of Imidazoles **3**

Triazole **3** (0.4 mmol) was dissolved in dry DCE (2 mL) in a microwave tube. Rh_2_(Oct)_4_ (0.004 mmol, 1 mol %) and the corresponding nitrile RCN (0.8 mmol, 2 equiv) were added, and the tube was closed and briefly sonicated. The reaction mixture was microwave heated at 140 °C, the solvent was evaporated under reduced pressure, and then the crude product was purified by column chromatography on silica gel.

#### 2,4-Diphenyl-1-(1,1,2,2-tetrafluoroethyl)-1*H*-imidazole (**3a**)

Reaction time: 30 min. Purified by column chromatography (cyclohexane) and obtained as a white solid. Yield: 272 mg, 85%. NMR spectra corresponded to the literature.^29^

#### 2-Phenyl-1-(1,1,2,2-tetrafluoroethyl)-4-(4-(trifluoromethyl)phenyl)-1*H*-imidazole (**3b**)

Reaction time: 1 h. Purified by column chromatography (cyclohexane) and obtained as a white solid. Yield: 330 mg, 85%. NMR spectra corresponded to the literature.^29^

#### 4-(4-Methoxyphenyl)-2-phenyl-1-(1,1,2,2-tetrafluoroethyl)-1*H*-imidazole (**3d**)

Reaction time: 1 h. Purified by column chromatography (cyclohexane/EtOAc, 3:1) and obtained as a white solid. Yield: 304 mg, 87%, mp 61 °C; ^1^H NMR (400 MHz, CDCl_3_): δ 7.84–7.77 (m, 2H), 7.69–7.60 (m, 2H), 7.55–7.45 (m, 4H), 7.01–6.94 (m, 2H), 5.78 (tt, *J* = 52.9, 3.1 Hz, 1H), 3.86 (s, 3H); ^13^C{^1^H} NMR (101 MHz, CDCl_3_): δ 159.5, 147.2, 142.1, 130.3, 130.1, 129.9, 128.4, 126.7, 125.2, 114.1, 112.6 (tt, *J* = 260.3, 29.0 Hz, CF_2_) 111.5, 107.8 (tt, *J* = 254.6, 42.5 Hz, CF_2_H), 55.3; ^19^F NMR (376 MHz, CDCl_3_): δ −92.0 (q, *J* = 5.1 Hz, 2F), −135.1 (dt, *J* = 53.0, 6.0 Hz, 2F); HRMS (EI^+^): calcd *m*/*z* for C_18_H_14_F_4_N_2_O [M]^+^ 350.1037; found, 350.1038.

#### 2-(4-Methoxyphenyl)-4-phenyl-1-(1,1,2,2-tetrafluoroethyl)-1*H*-imidazole (**3e**)

Reaction time: 30 min. Purified by column chromatography (cyclohexane/EtOAc, 9:1) and obtained as a colorless oil. Yield: 294 mg, 84%; ^1^H NMR (500 MHz, CDCl_3_): δ 7.87–7.81 (m, 2H), 7.53 (s, 1H), 7.43–7.28 (m, 4H), 7.22–7.13 (m, 2H), 7.04 (ddt, *J* = 8.3, 2.6, 0.8 Hz, 1H), 5.76 (tt, *J* = 52.9, 3.3 Hz, 1H), 3.84 (s, 3H); ^13^C{^1^H} NMR (126 MHz, CDCl_3_): δ 159.5, 147.2, 142.2, 131.4, 129.7, 128.8, 128.0, 125.5, 122.3, 116.4, 115.3, 112.3 (tt, *J* = 267.5, 29.0 Hz, CF_2_), 107.7 (tt, *J* = 257, 4 Hz, CF_2_H), 55.5; ^19^F NMR (376 MHz, CDCl_3_): δ −92.0 (q, *J* = 5.2 Hz, 2F), −135.2 (dt, *J* = 53.0, 6.1 Hz, 2F); HRMS (EI^+^): *m*/*z* calcd for C_18_H_14_F_4_N_2_O [M]^+^ 350.1037; found, 350.1038.

#### 2-(4-Fluorophenyl)-4-phenyl-1-(1,1,2,2-tetrafluoroethyl)-1*H*-imidazole (**3f**)

Reaction time: 45 min. Purified by column chromatography (cyclohexane/EtOAc, 9:1) and obtained as a pale-yellow oil. Yield: 253 mg, 75%; ^1^H NMR (400 MHz, CDCl_3_): δ 7.88–7.83 (m, 2H), 7.64 (ddq, *J* = 8.2, 4.0, 1.4 Hz, 2H), 7.54 (m, 1H), 7.48–7.30 (m, 3H), 7.24–7.14 (m, 2H), 5.85 (tt, *J* = 53.0, 2.5 Hz, 1H); ^13^C{^1^H} NMR (101 MHz, CDCl_3_): δ 165.0, 162.5, 146.6, 142.3, 132.2, 132.0 (dt, *J* = 8.6, 2.1 Hz), 128.8, 128.0, 126.3 (d, *J* = 3.4 Hz), 125.4, 115.6 (d, *J* = 22.0 Hz), 112.7, 110.0 (tt, *J* = 256.0, 28.6 Hz), 107.9 (tt, *J* = 258.0, 43.4 Hz); ^19^F NMR (376 MHz, CDCl_3_): δ −91.8 (q, *J* = 4.5 Hz, 2F), −110.1 (tt, *J* = 8.4, 5.2 Hz, 2F), −134.6 (dt, *J* = 52.9, 5.3 Hz, 2F); HRMS (NSI^+^): *m*/*z* calcd for C_17_H_12_F_5_N_2_ [M]^+^ 339.0914; found, 339.09152.

#### 2-Methyl-4-phenyl-1-(1,1,2,2-tetrafluoroethyl)-1*H*-imidazole (**3g**)

Reaction time: 3 h with MeCN (10 equiv). Purified by column chromatography (cyclohexane/EtOAc, 1:1) and obtained as a slightly brown oil, which decomposed. Yield: 39 mg, 15%; ^1^H NMR (400 MHz, CDCl_3_): δ 7.79–7.75 (m, 2H), 7.45–7.37 (m, 2H), 7.32 (m, 2H), 6.11 (tt, *J* = 53.2, 1.3 Hz, 1H), 2.60 (t, *J* = 2.4 Hz, 3H); ^13^C{^1^H} NMR (101 MHz, CDCl_3_): δ 145.4, 141.4, 132.5, 128.7, 127.7, 125.2, 112.7 (tt, *J* = 260.3, 30.0 Hz, CF_2_), 111.9, 108.5 (tt, *J* = 253.8, 46.5 Hz, CF_2_H), 15.1; ^19^F NMR (376 MHz, CDCl_3_): δ −95.36 (s, 2F), −134.15 (dt, *J* = 53.1, 4.2 Hz, 2F); HRMS (EI^+^): *m*/*z* calcd for C_12_H_10_F_4_N_2_ [M]^+^ 258.0775; found, 258.0776.

### Synthesis of (*Z*)-2-(2,2-Difluoroacetamido)-1-phenylvinyl Trifluoromethanesulfonate (**4**)

A solution of trifluoromethanesulfonic acid (0.025 mL, 0.32 mmol, 1 equiv) in DCE (1 mL) was added dropwise to a screw-cap glass tube containing a solution of **3a** (80 mg, 0.32 mmol, 1 equiv) in DCE (2 mL). During the addition, a suspension was formed. The reaction mixture was stirred for 12 h at room temperature. Et_2_O was added (20 mL), and the solution was extracted with water (2 × 10 mL). The organic phase was separated, dried over Na_2_SO_4_, filtered, and evaporated on silica gel, and the product was isolated by column chromatography (cyclohexane/EtOAc, 9:1) and obtained as a white solid. Yield: 252 mg, 73%, mp 65 °C; ^1^H NMR (400 MHz, CDCl_3_): δ 8.32 (d, *J* = 11.3 Hz, 1H), 7.54–7.41 (m, 5H), 7.41–7.31 (m, 1H), 6.08 (t, *J* = 53.9 Hz, 1H); ^13^C{^1^H} NMR (101 MHz, CDCl_3_): δ 159.8 (t, *J* = 26.1 Hz), 135.8, 130.7, 130.0, 129.1, 125.0, 118.4 (q, *J* = 320.3 Hz, CF_3_), 112.9, 107.8 (t, *J* = 253.2 Hz, CF_2_H); ^19^F NMR (376 MHz, CDCl_3_): δ −73.47 (s, 3F), −126.92 (d, *J* = 53.8 Hz, 2F); HRMS (ESI^–^): *m*/*z* calcd for C_11_H_8_F_5_NO_4_S [M]^−^ 345.0089; found, 345.0092.

### General Procedure for the Synthesis of Tetrazoles **5**

Under a nitrogen atmosphere, a precooled high-pressure tube with a stirring bar was filled with the solution of **1** (0.69 mmol) in THF (1 mL). Primary amine (0.69 mmol, 1 equiv) and Et_3_N (139.4 mg, 1.38 mmol, 2 equiv) were added. The tube was closed and stirred overnight at 40 °C (aluminum block, heating mantle). The solvent was evaporated, and then the reaction mixture was diluted with Et_2_O and washed with 5% aqueous HCl solution and then with water. The combined extracts were dried over Na_2_SO_4_, and the solvent was evaporated. The crude tetrazoles were purified by column chromatography or crystallization.

#### Butyl-5-(difluoromethyl)-1*H*-tetrazole (**5a**)

Purified by column chromatography (cyclohexane/EtOAc, 9:1) and obtained as a colorless oil. Yield: 120 mg, 68%; ^1^H NMR (400 MHz, CDCl_3_): δ 7.14 (t, *J* = 52.1 Hz, 1H), 4.55 (t, *J* = 7.4 Hz, 2H), 2.05–1.93 (m, 2H), 1.42 (m, 2H), 0.99 (t, *J* = 7.4 Hz, 3H); ^13^C{^1^H} NMR (101 MHz, CDCl_3_): δ 148.0 (t, *J* = 29.2 Hz), 107.4 (t, *J* = 239.1 Hz), 48.9, 31.7, 29.7, 19.5, 13.3; ^19^F NMR (376 MHz, CDCl_3_): δ −116.5 (d, *J* = 51.9 Hz); HRMS (APCI^+^): *m*/*z* calcd for C_6_H_11_F_2_N_4_ [M]^+^ 177.0945; found, 177.0946.

#### 1-Cyclohexyl-5-(difluoromethyl)-1*H*-tetrazole (**5b**)

Purified by recrystallization from pentane and obtained as a white solid. Yield: 152 mg, 75%, mp 62 °C; ^1^H NMR (500 MHz, CDCl_3_): δ 7.10 (t, *J* = 52.1 Hz, 1H), 4.57 (tt, *J* = 11.5, 4.2 Hz, 1H), 2.14–1.94 (m, 6H), 1.77 (m, 1H), 1.50–1.27 (m, 3H); ^13^C{^1^H} NMR δ (126 MHz, CDCl_3_): δ 147.5 (t, *J* = 29.1 Hz), 107.6 (t, *J* = 238.8 Hz), 60.2 (d, *J* = 2.2 Hz), 33.1, 25.3, 24.8; ^19^F NMR (376 MHz, CDCl_3_): δ −116.5 (d, *J* = 52.1 Hz); HRMS (NSI^+^): *m*/*z* calcd for C_8_H_13_F_2_N_4_ [M]^+^ 203.1099; found, 203.1099.

#### Benzyl-5-(difluoromethyl)-1*H*-tetrazole (**5c**)

Purified from byproduct **6c**. To the crude reaction mixture in CH_2_Cl_2_ (5 mL) was added a solution of 1 N NaOH (0.6 mmol, 1 equiv), and the mixture was stirred at rt for 18 h. The solution was extracted with DCM (2 × 15 mL) and washed with water (2 × 10 mL). The organic phase was dried over MgSO_4_, filtered, and concentrated to dryness under reduced pressure. The title product was obtained as a white amorphous solid. Yield: 126 mg, 60%; ^1^H NMR (400 MHz, CDCl_3_): δ 7.45–7.32 (m, 5H), 7.08 (t, *J* = 52.2 Hz, 1H), 5.72 (s, 2H); ^13^C{^1^H} NMR (101 MHz, CDCl_3_): δ 148.0 (t, *J* = 29.3 Hz), 132.5, 129.4, 129.2, 128.3, 107.3 (t, *J* = 239.9 Hz), 52.6 (t, *J* = 2.0 Hz), 29.70; ^19^F NMR (376 MHz, CDCl_3_): δ −116.1 (d, *J* = 52.1 Hz); HRMS (EI^+^): *m*/*z* calcd for C_9_H_8_F_2_N_4_ [M]^+^ 210.0712; found, 210.0710.

#### 5-(Difluoromethyl)-1-(4-methylbenzyl)-1*H*-tetrazole (**5d**)

Purified from byproduct **6d**. To the crude reaction mixture in CH_2_Cl_2_ (5 mL) was added the solution of 1 N NaOH (0.6 mmol, 1 equiv), and the mixture was stirred at rt for 18 h. The solution was extracted with DCM (2 × 15 mL) and washed with water (2 × 10 mL). The organic phase was dried over MgSO_4_, filtered, and concentrated to dryness under reduced pressure. The title product was obtained as a white solid. Yield: 179 mg, 80%, mp 120 °C; ^1^H NMR (400 MHz, CDCl_3_): δ 7.30–7.20 (m, 5H), 7.06 (t, *J* = 52.4 Hz), 5.68 (s, 2H), 2.37 (s, 3H); ^13^C{^1^H} NMR (101 MHz, CDCl_3_): δ 147.9 (t, *J* = 29.4 Hz), 139.4, 129.8, 129.5, 128.3, 107.3 (t, *J* = 243.6 Hz), 52.4 (t, *J* = 1.9 Hz), 21.2; ^19^F NMR (376 MHz, CDCl_3_): δ −116.2 (d, *J* = 52.0 Hz); HRMS (APCI^+^): *m*/*z* calcd for C_10_H_11_F_2_N_4_ [M]^+^ 225.0945; found, 225.0946.

## Data Availability

The data underlying this study are available in the published article and its Supporting Information.

## Supporting Information Available

Copies of NMR spectra and crystallographic data for **5b** (PDF)

## Accession Codes

CCDC 2239780 contains the supplementary crystallographic data for this paper. These data can be obtained free of charge via www.ccdc.cam.ac.uk/data_request/cif, or by emailing data_request@ccdc.cam.ac.uk, or by contacting The Cambridge Crystallographic Data Centre, 12 Union Road, Cambridge CB2 1EZ, UK; fax: +44 1223 336033.
