# Supplementary material for: Synthesis and Cycloaddition Reactions of 1-Azido-1,1,2,2-tetrafluoroethane
Source: J Org Chem. 2023 Oct 20;88(21):14969–77. doi: 10.1021/acs.joc.3c01346 (PMC10629226; doi:10.1021/acs.joc.3c01346)
Supplement: Supplementary file 1 — jo3c01346_si_001.pdf [file jo3c01346_si_001.pdf]

Synthesis and Cycloaddition Reactions of 1-Azido-1,1,2,2-tetrafluoroethane

Elena Shaitanova,[†‡] Václav Matoušek,[§] Tadeáš Herentin,[§] Martin Adamec,[§] Robert Matyáš,[⁋] Blanka Klepetářová,[†] Petr Beier[†*]

[†] *Institute of Organic Chemistry and Biochemistry of the Czech Academy of Sciences, Flemingovo náměstí, 2, 166 10, Prague 6, Czech Republic.*

[‡] *V. P. Kukhar Institute of Bioorganic Chemistry and Petrochemistry, The National Academy of Sciences of Ukraine, Academika Kukhara Str. 1, Kyiv, Ukraine.*

[§] *CF Plus Chemicals s.r.o., Karásek 1767/1, 621 00 Brno, Czech Republic.*

[⁋] *Institute of Energetic Materials, University of Pardubice, Doubravice 41, 532 10 Pardubice, Czech Republic.*

[*] *Corresponding author's e-mail address: beier@uochb.cas.cz*

KEYWORDS: Tetrafluoroethylene; Azide; Triazole; Imidazole; Tetrazole; Cycloaddition

Supporting information

1. Thermal stability of $HCF_2CF_2N_3$……………………………………………………..……S2
2. Sensitivity of $HCF_2CF_2N_3$ to impact and heat …………………………………………..……S3
3. Crystallographic data of **5b**……………………………………………………………………S3
4. Copies of NMR spectra ..…………………………………………………………………….S5
5. References ……………………………………………………….…………………...........S75

1. Thermal stability of $HCF_2CF_2N_3$

A solution of azide **1** (0.21 mmol, 0.2 mL in THF) was placed in a pre-cooled (-40 °C) Norrell S600 low pressure/vacuum NMR tube containing $CDCl_3$ (0.4 mL) and tightly closed. After reaching rt, the ^{19}F NMR spectrum was measured and the tube was heated to 150 °C in an oil bath. After 2 hours, the tube cooled to rt, and the ^{19}F NMR spectrum recorded. The experiment was repeated after 4 and 8 hours. In all cases the studied azide was stable and no changes in ^{19}F NMR spectra (chemical shifts or signal intensities) were observed (see Figure S1).

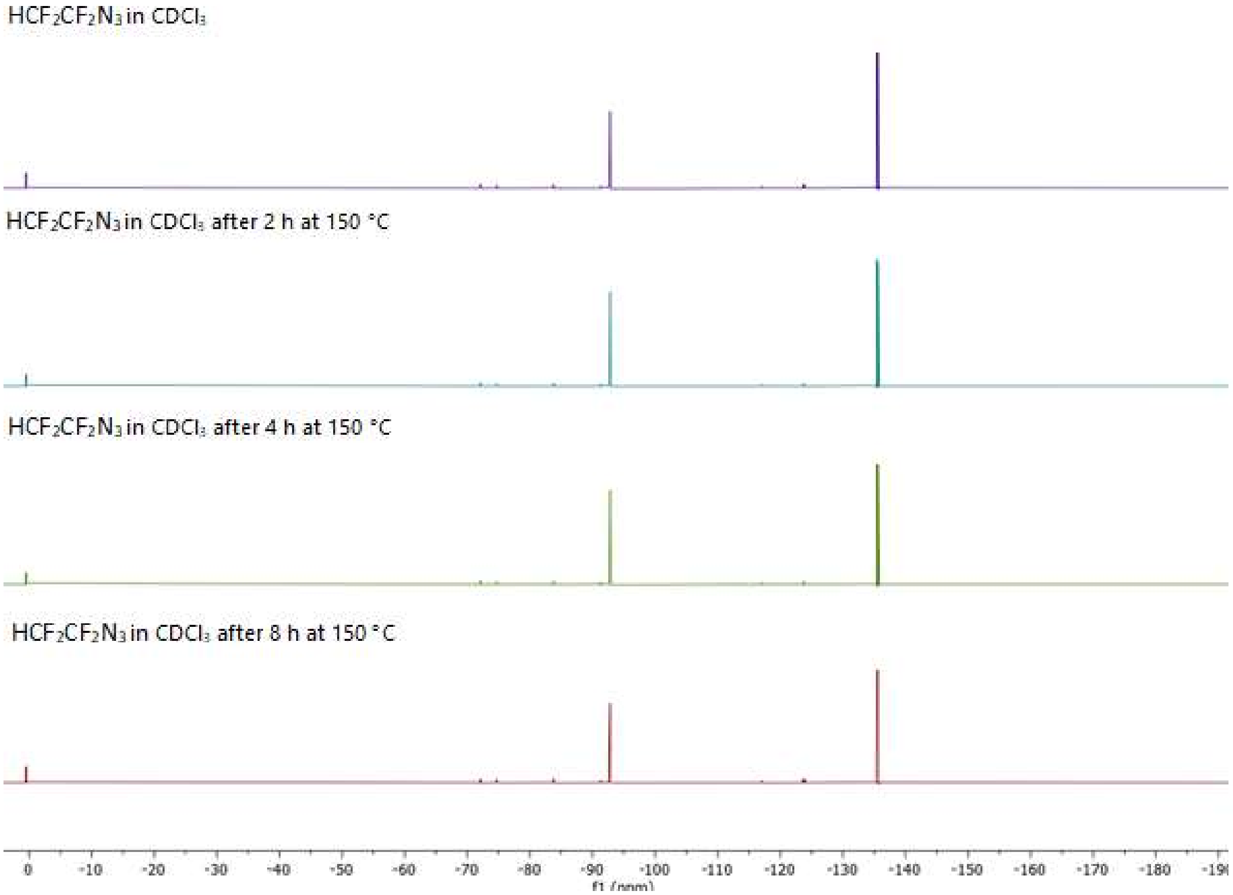

Figure S1. Determination of thermal stability of **1** by ^{19}F NMR.

2. Sensitivity of $HCF_2CF_2N_3$ to impact and heat

The Koenen test apparatus (sensitivity to heat) was produced by Explosia company (Pardubice, Czech Republic) and the steel test tube assembly was produced by OZM Research. The steel tube was filled with a solution of **1** in THF (0.5M, 27 mL). The tube was then closed using a closing device with an orifice 2 mm in diameter. The test tube assembly was then put into a heating device and the burners were ignited. The measuring apparatus, related supplies, and the measurement methodology were in compliance with the UN[1] and EC[2] regulations. The result of the test was negative (Figure S2).

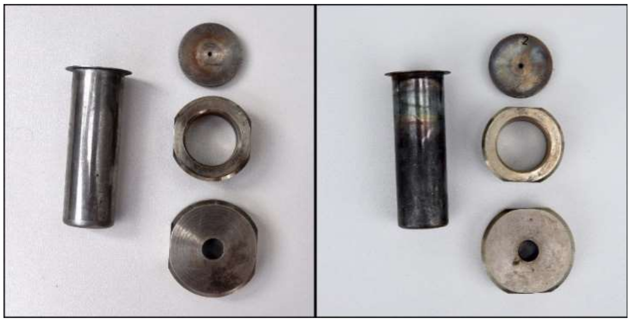

Figure S2. Koenen test (sensitivity to heat) of **1**. Individual components of the test tube assembly before (left) and after (right) the test of THF solution of **1** (0.5 M).

Sensitivity to impact was measured using a Kast fall hammer BFH-12, the testing set comprised of BFH-SC steel guide rings and BFH-SR steel cylinders. The measuring apparatus and all related supplies were acquired by OZM Research (Hrochův Týnec, Czech Republic). The measuring apparatus, related supplies, and measurement methodology were in compliance with the UN[1] and EC[2] regulations. The result of the test was negative and azide **1** was insensitive to impact of energy of 50 J.

3. X-ray crystallography of 5b

Single-crystal diffraction data of **5b** were collected using Bruker D8 VENTURE system equipped with a Photon 100 CMOS detector, a multilayer monochromator, and a CuKα Incoatec microfocus sealed tube (λ = 1.54178 Å) at 180 K. The frames were integrated with the Bruker SAINT software package.[3] The structure was solved by direct methods with SIR92[4] and were refined by full-matrix

least-squares on F with CRYSTALS.[5] The positional and anisotropic thermal parameters of all non-hydrogen atoms were refined. All hydrogen atoms were located in a difference Fourier map and then they were repositioned geometrically. They were initially refined with soft restraints on the bond lengths and angles to regularise their geometry, then their positions were refined with riding constraints

Crystal data for **5b** (colorless, 0.040 × 0.084 × 0.261 mm): $C_8H_{12}F_2N_4$, triclinic, space group *P*-1, $a = 6.5146(3)$ Å, $b = 6.7994(3)$ Å, $c = 11.3753(5)$ Å, $\alpha = 82.9563(19)°$, $\beta = 73.9497(19)°$, $\gamma = 88.4340(19)°$, $V = 480.57(2)$ Å^3, $Z = 2$, $M = 202.21$, 16885 reflections measured, 1764 independent reflections. Final $R = 0.038$, $wR = 0.038$, $GoF = 1.084$ for 1612 reflections with $I > 2\sigma(I)$ and 128 parameters. CCDC 2239780 (Figure S3).

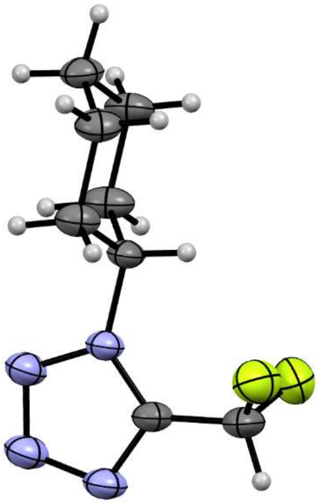

Figure S3. Crystal structure of 1-cyclohexyl-5-(difluoromethyl)-1*H*-tetrazole (**5b**). Ellipsoids are shown at 50% probability level.

4. Copies of NMR spectra

^{1}H NMR spectrum of **1** ($CDCl_3$, 400 MHz)

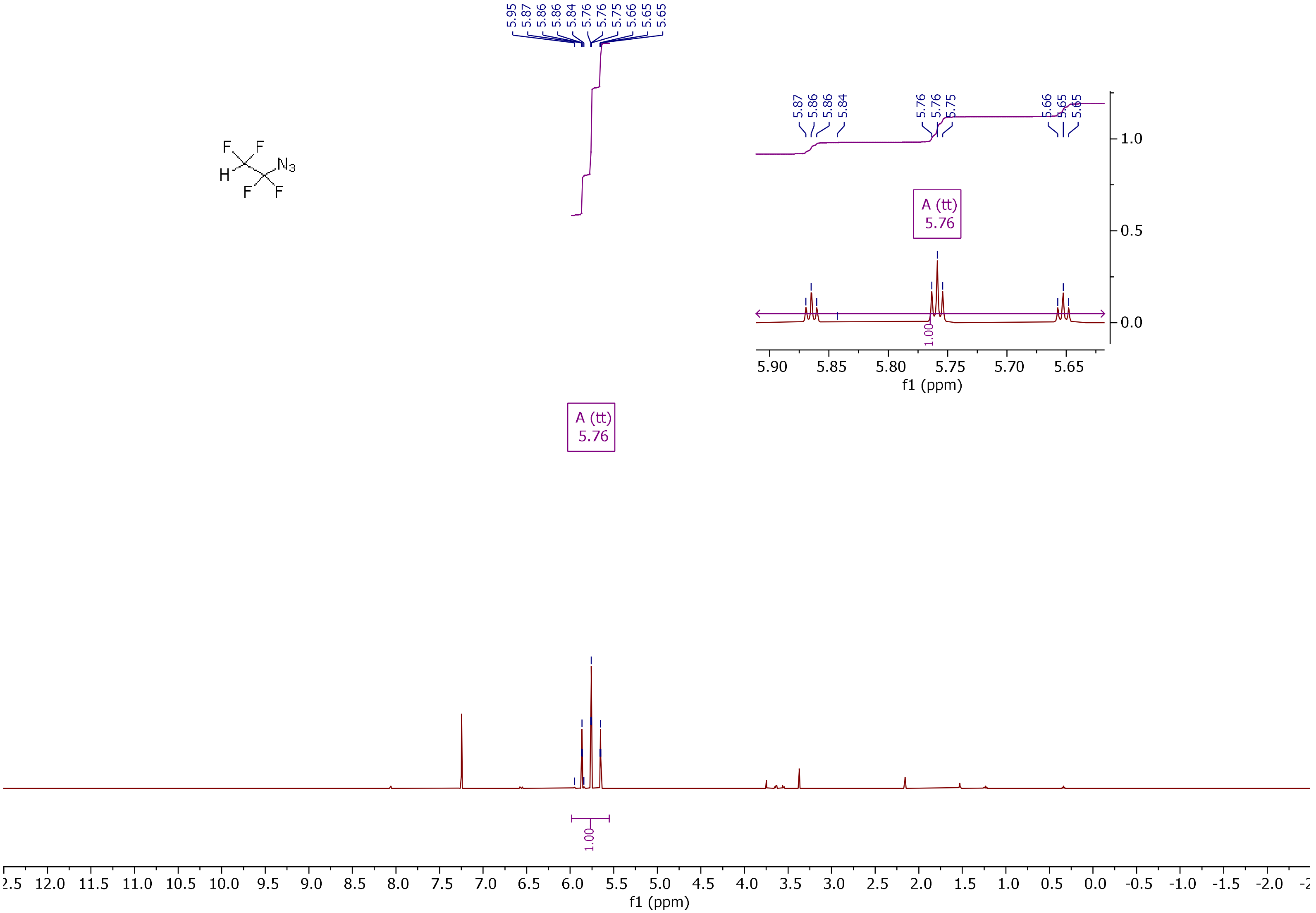

^{19}F NMR spectrum of **1** ($CDCl_3$, 377 MHz)

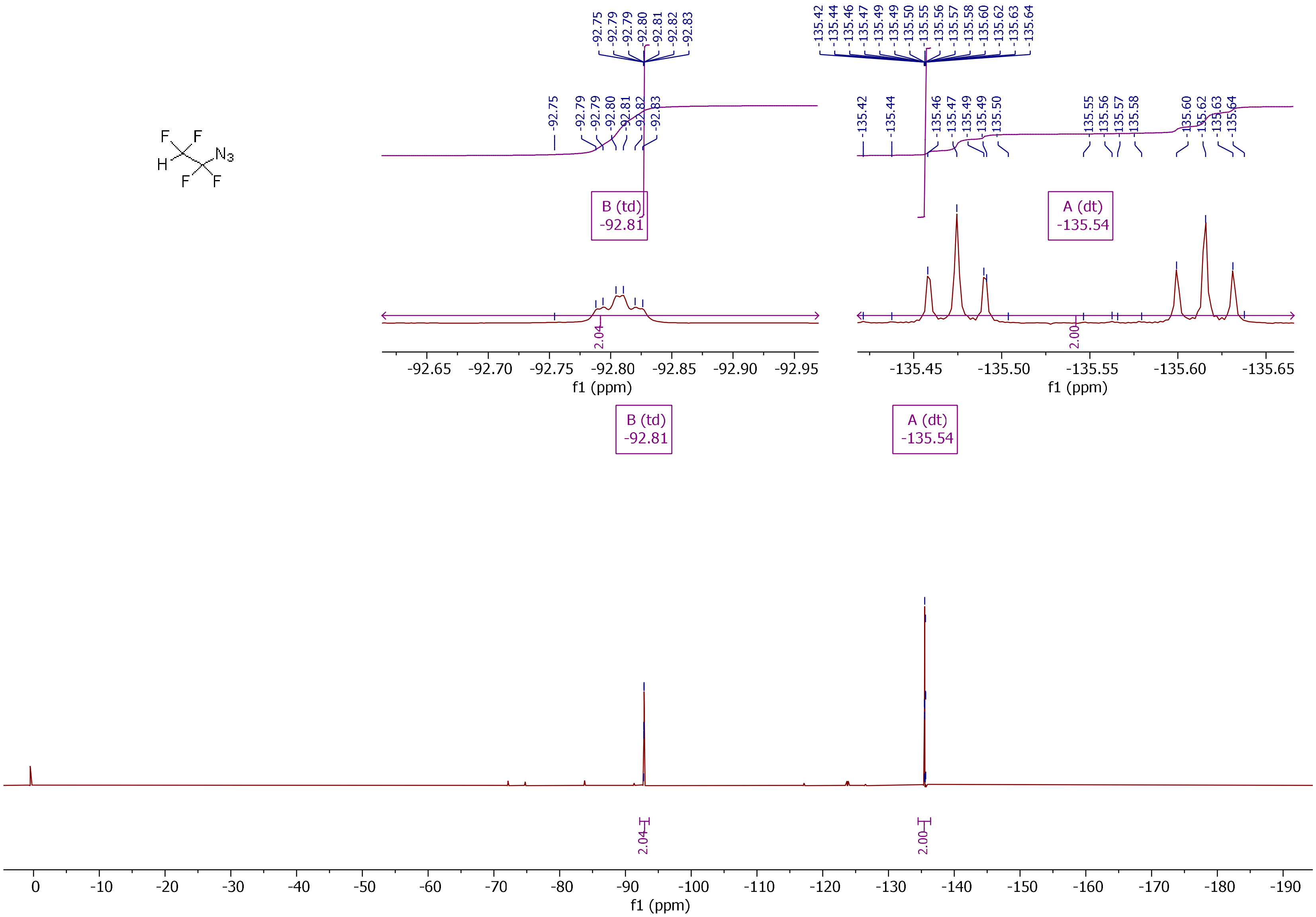

^{19}F NMR spectrum of **1** in THF solution with PhCF3 as a standard ($CDCl_3$, 377 MHz)

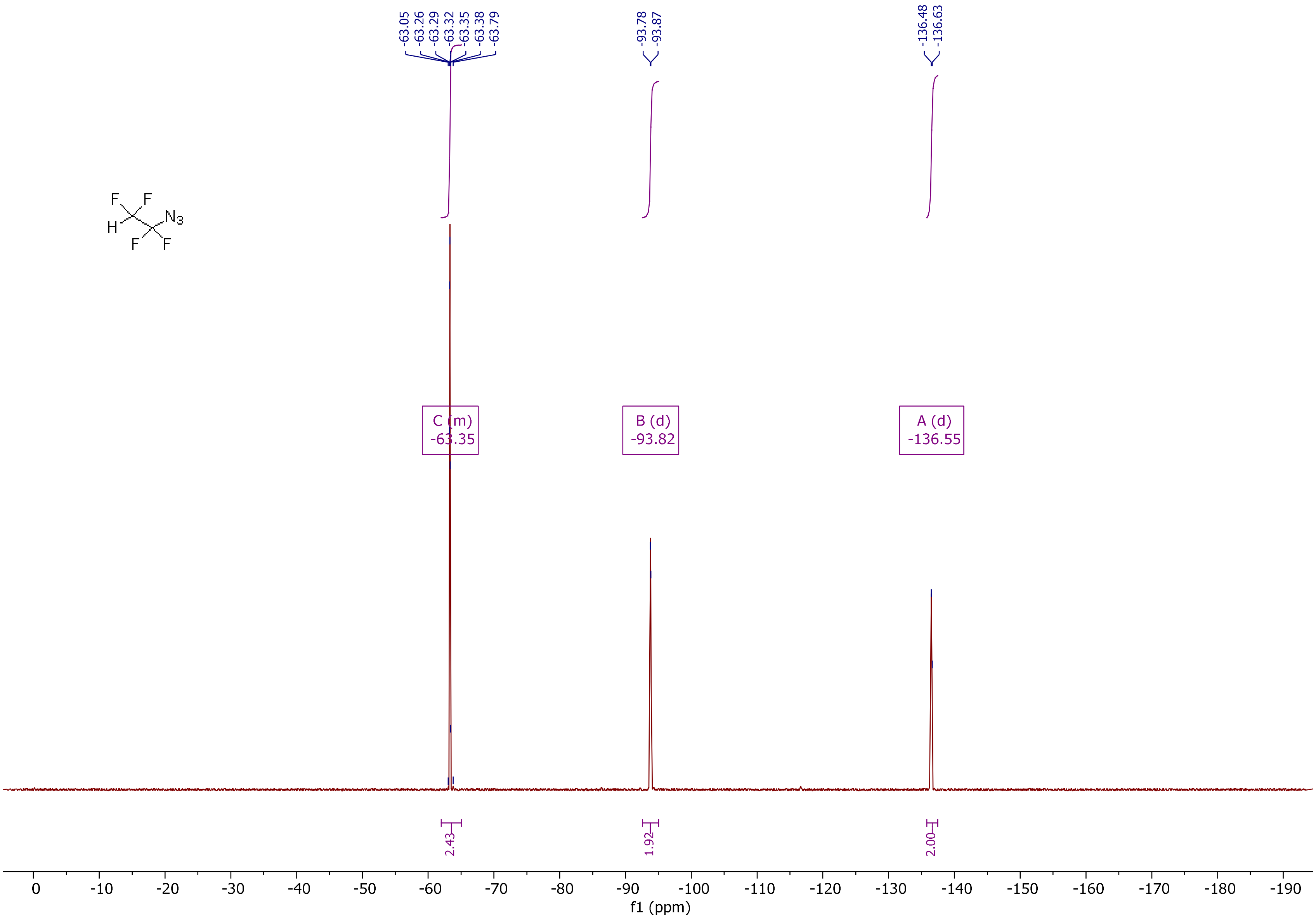

$^{13}C\{^{1}H\}$ NMR spectrum of **1** ($CDCl_3$, 101 MHz)

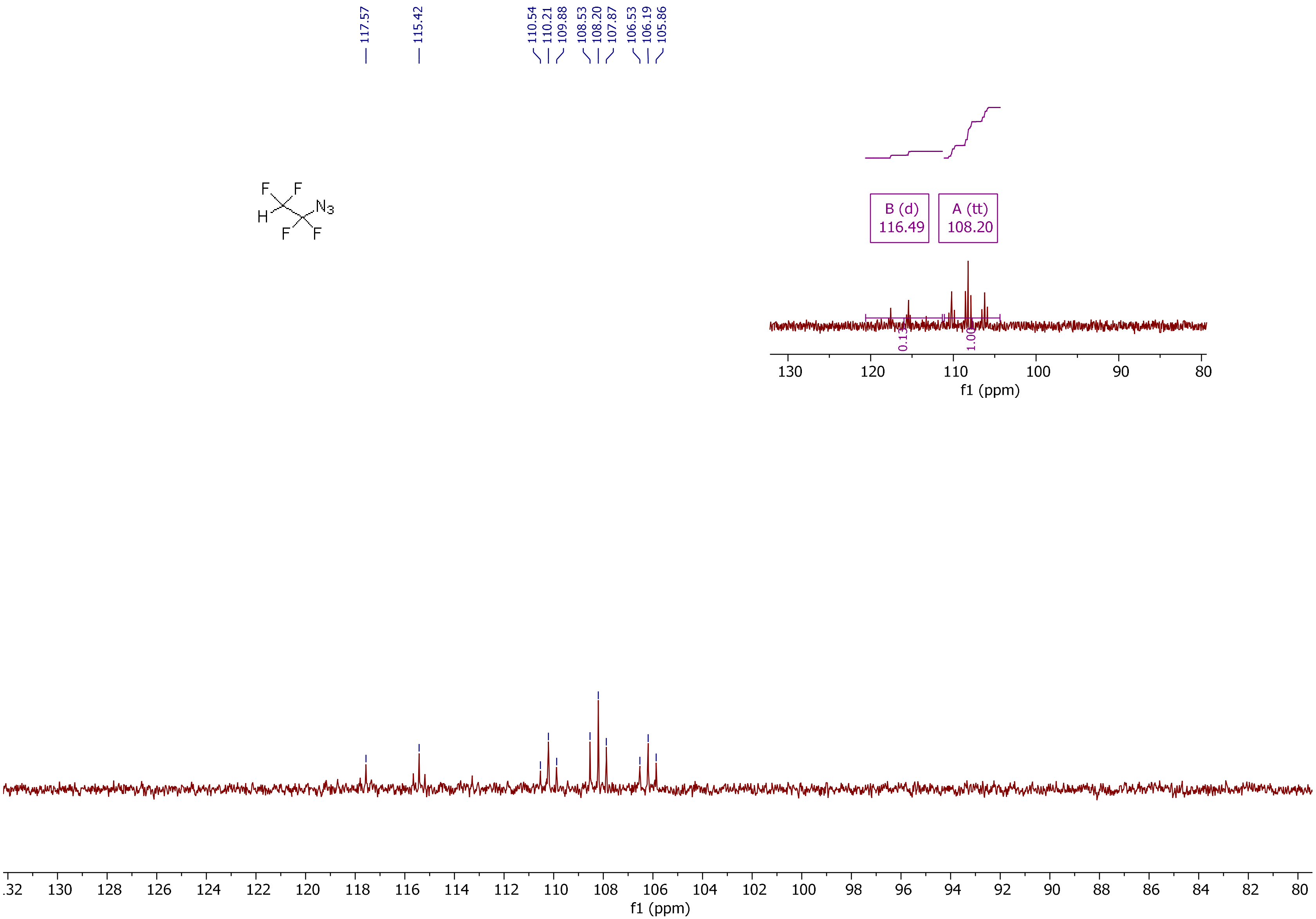

^1H NMR spectrum of **2a** ($CDCl_3$, 400 MHz)

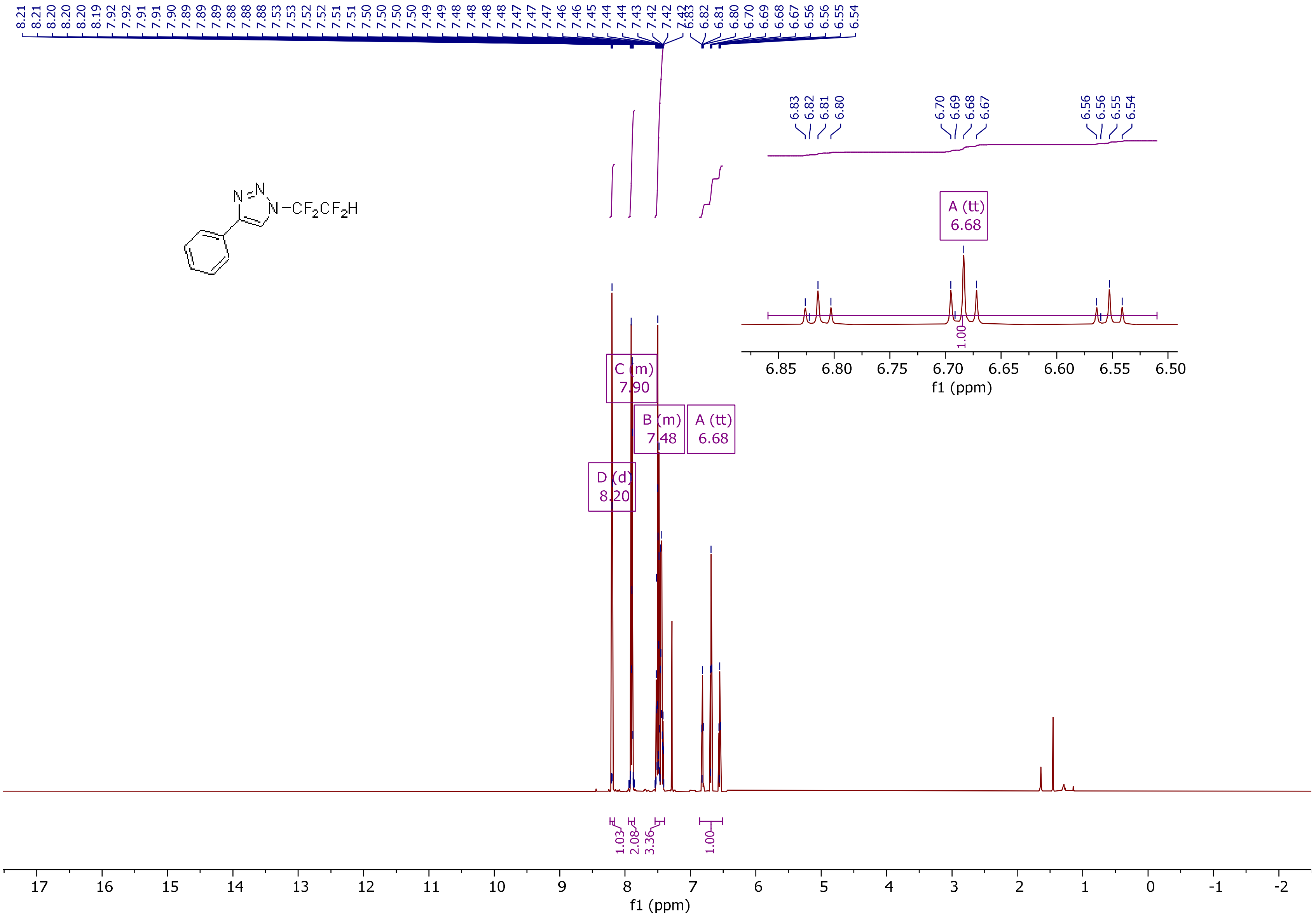

^{19}F NMR spectrum of **2a** ($CDCl_3$, 377 MHz)

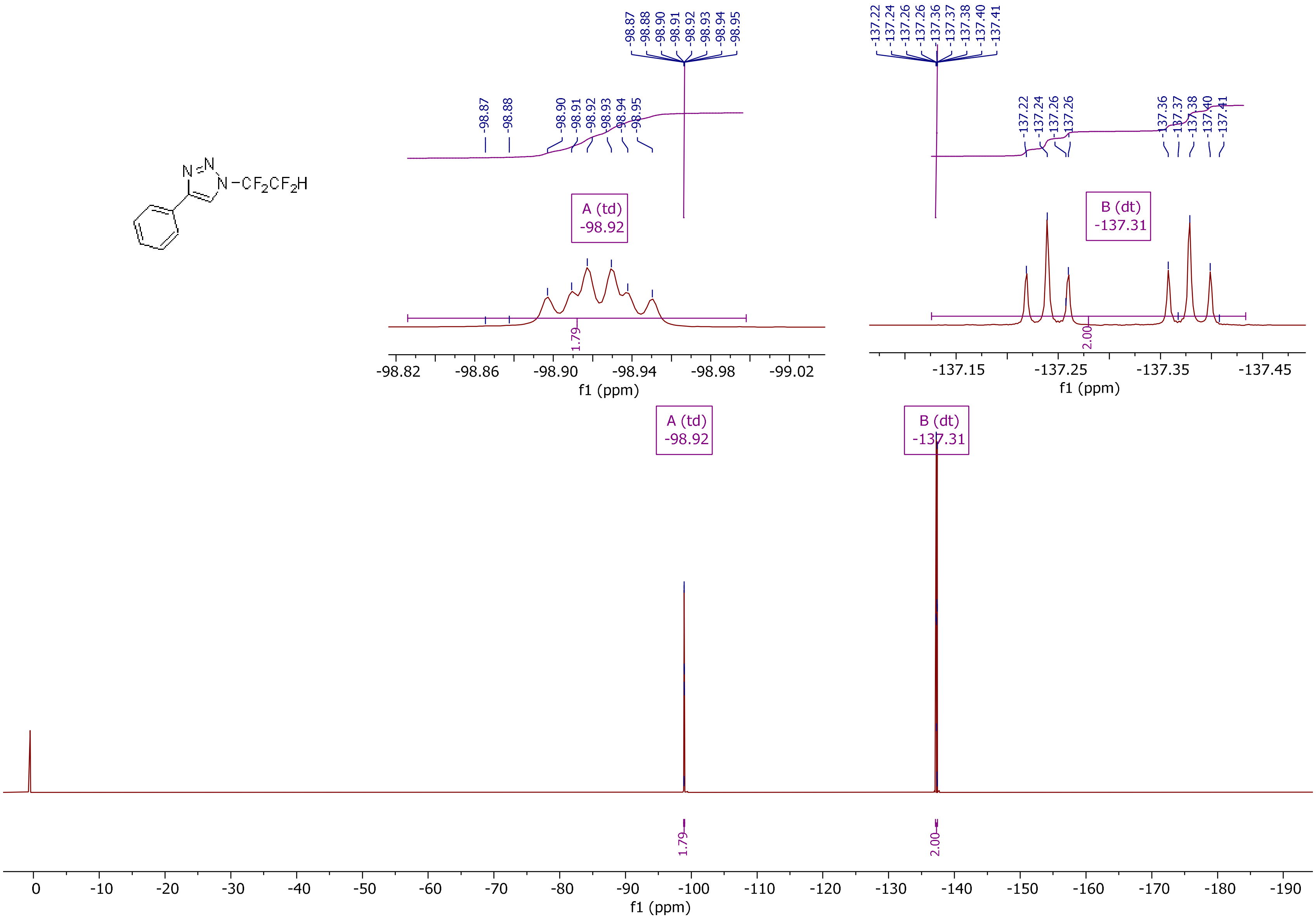

$^{13}C\{^1H\}$ NMR spectrum of **2a** ($CDCl_3$, 101 MHz)

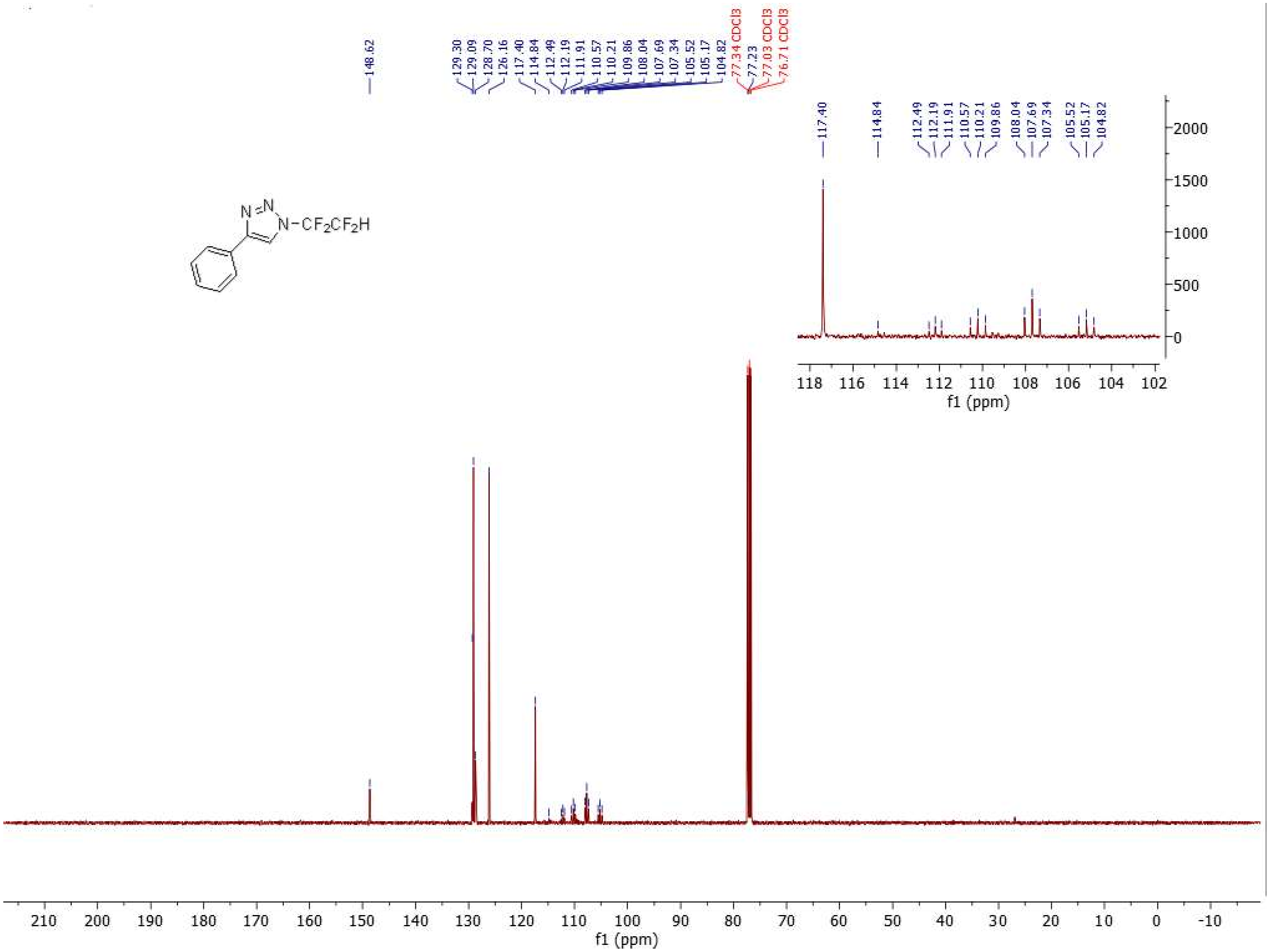

^{1}H NMR spectrum of **2b** ($CDCl_3$, 400 MHz)

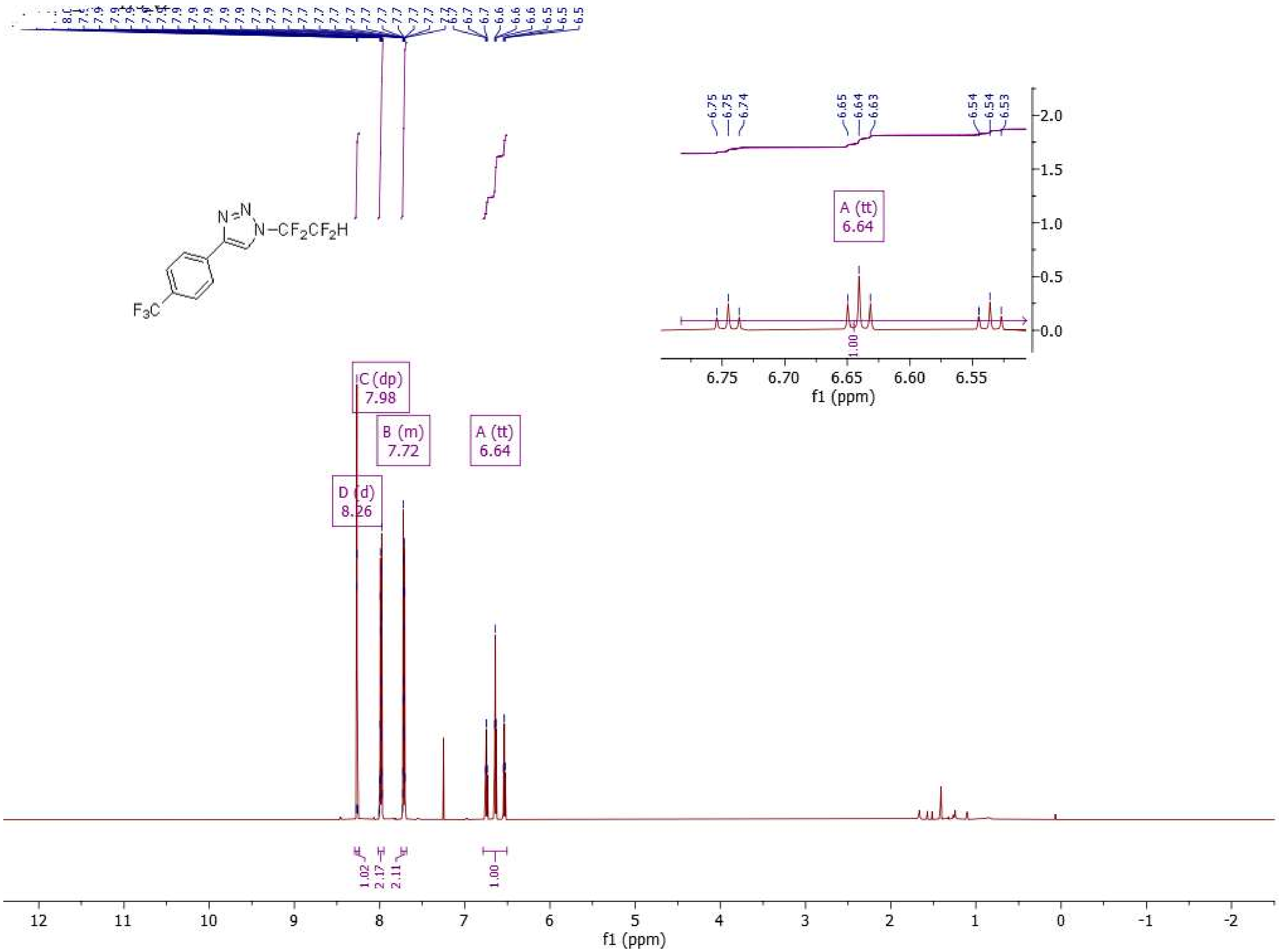

^{19}F NMR spectrum of **2b** ($CDCl_3$, 377 MHz)

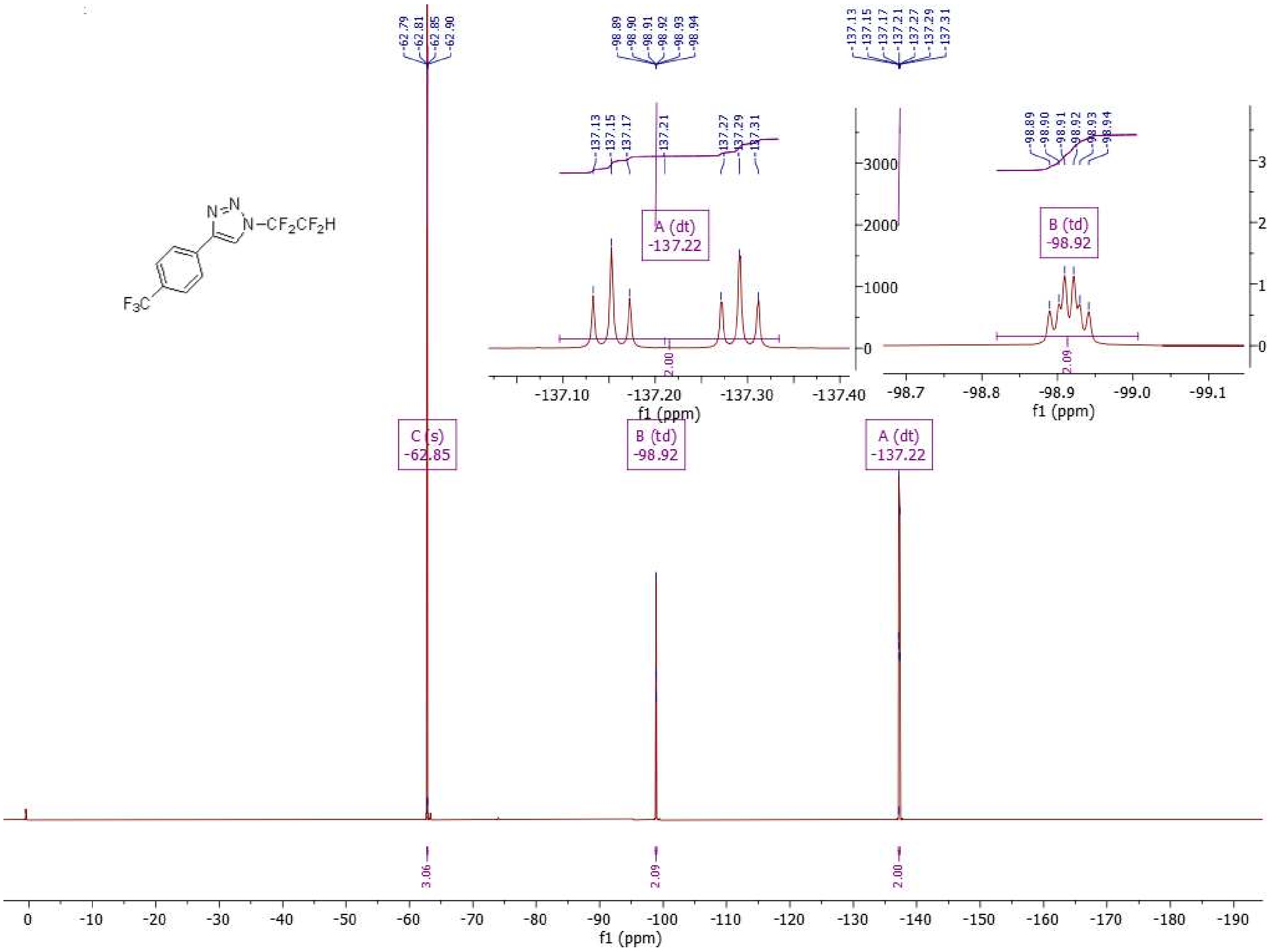

$^{13}C\{^1H\}$ NMR spectrum of **2b** ($CDCl_3$, 101 MHz)

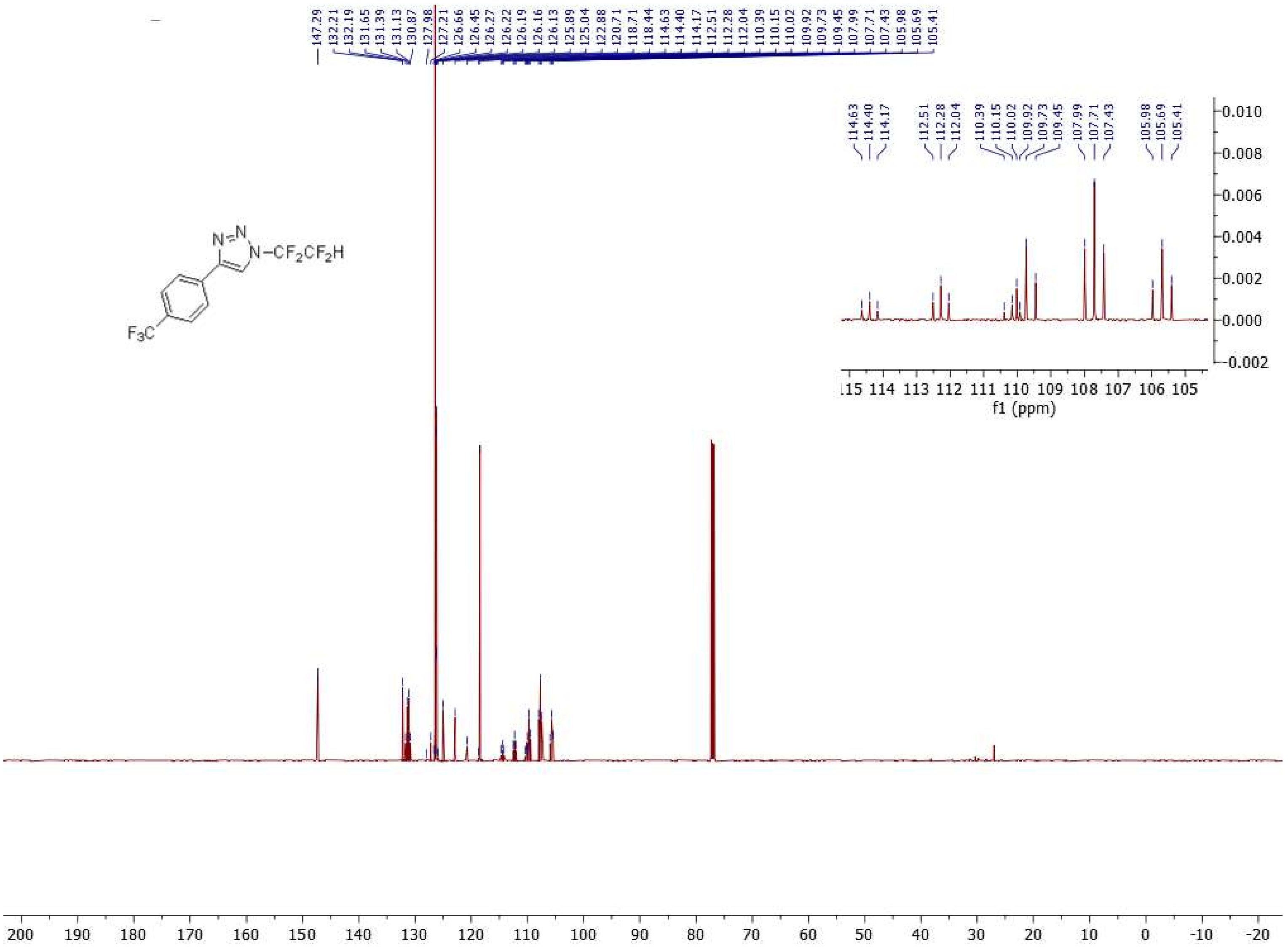

^{1}H NMR spectrum of **2c** ($CDCl_3$, 400 MHz)

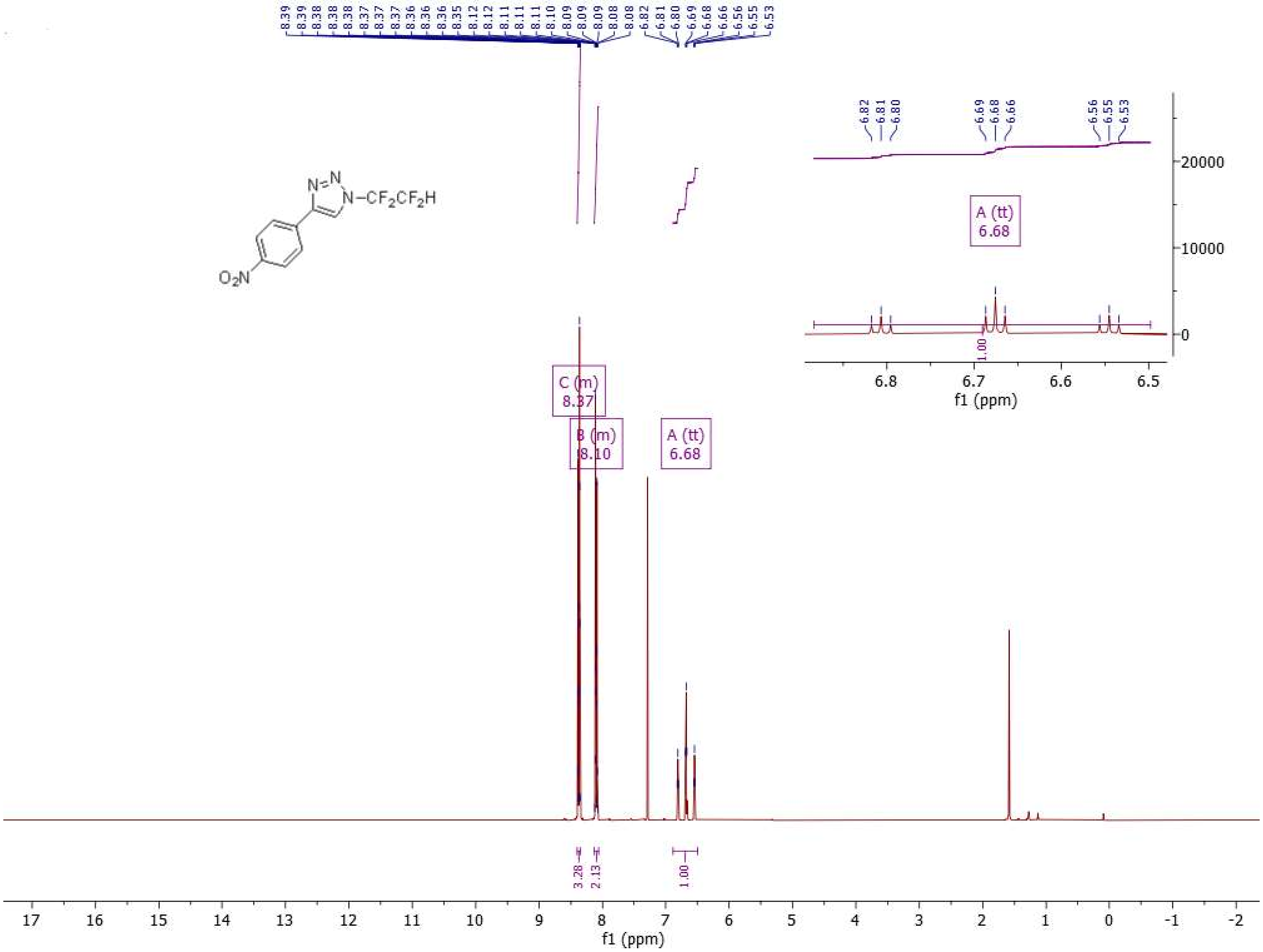

^{19}F NMR spectrum of **2c** ($CDCl_3$, 377 MHz)

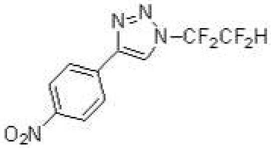

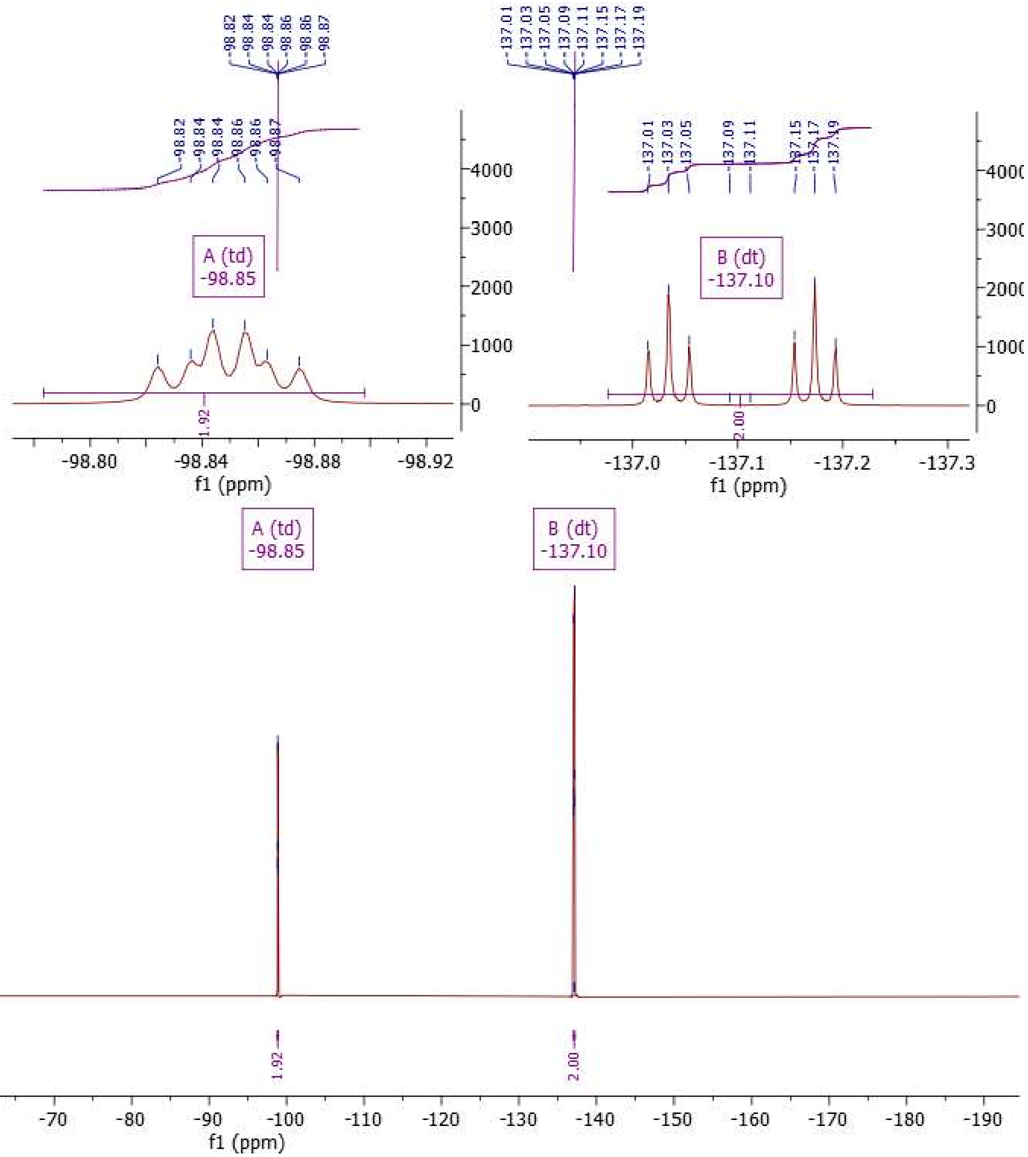

$^{13}C\{^{1}H\}$ NMR spectrum of **2c** ($CDCl_3$, 101 MHz)

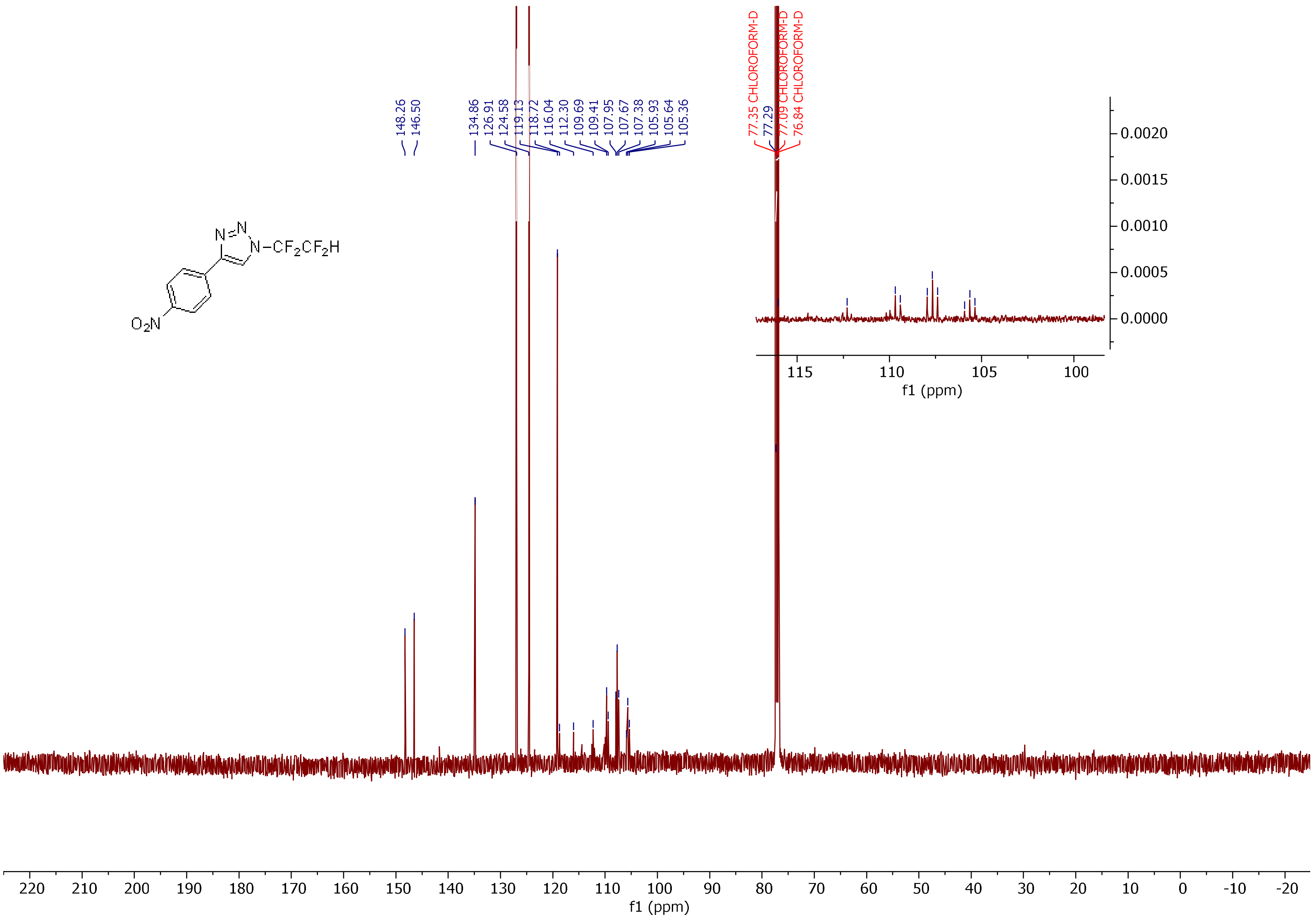

^{1}H NMR spectrum of **2d** ($CDCl_3$, 400 MHz)

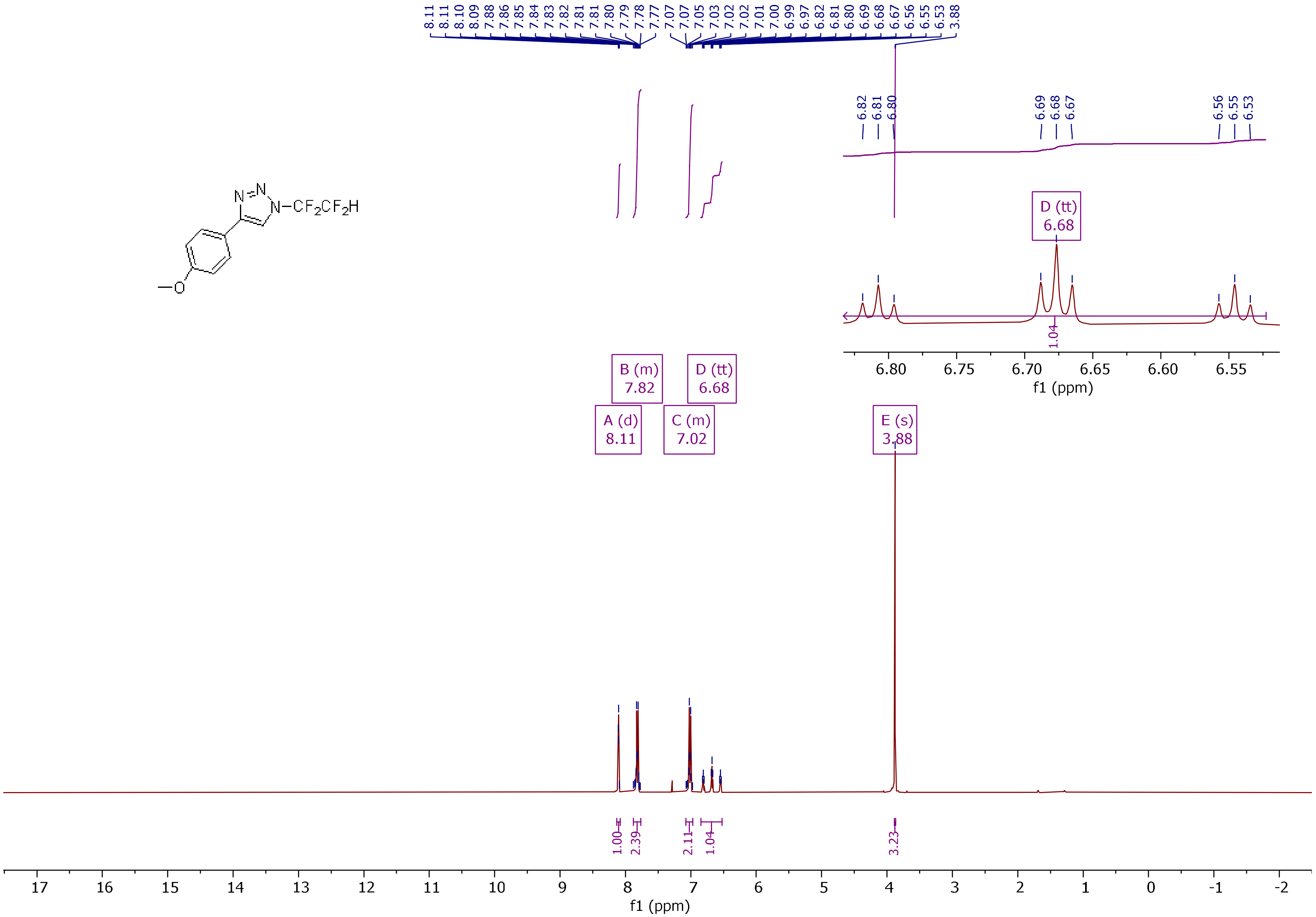

^{19}F NMR spectrum of **2d** ($CDCl_3$, 377 MHz)

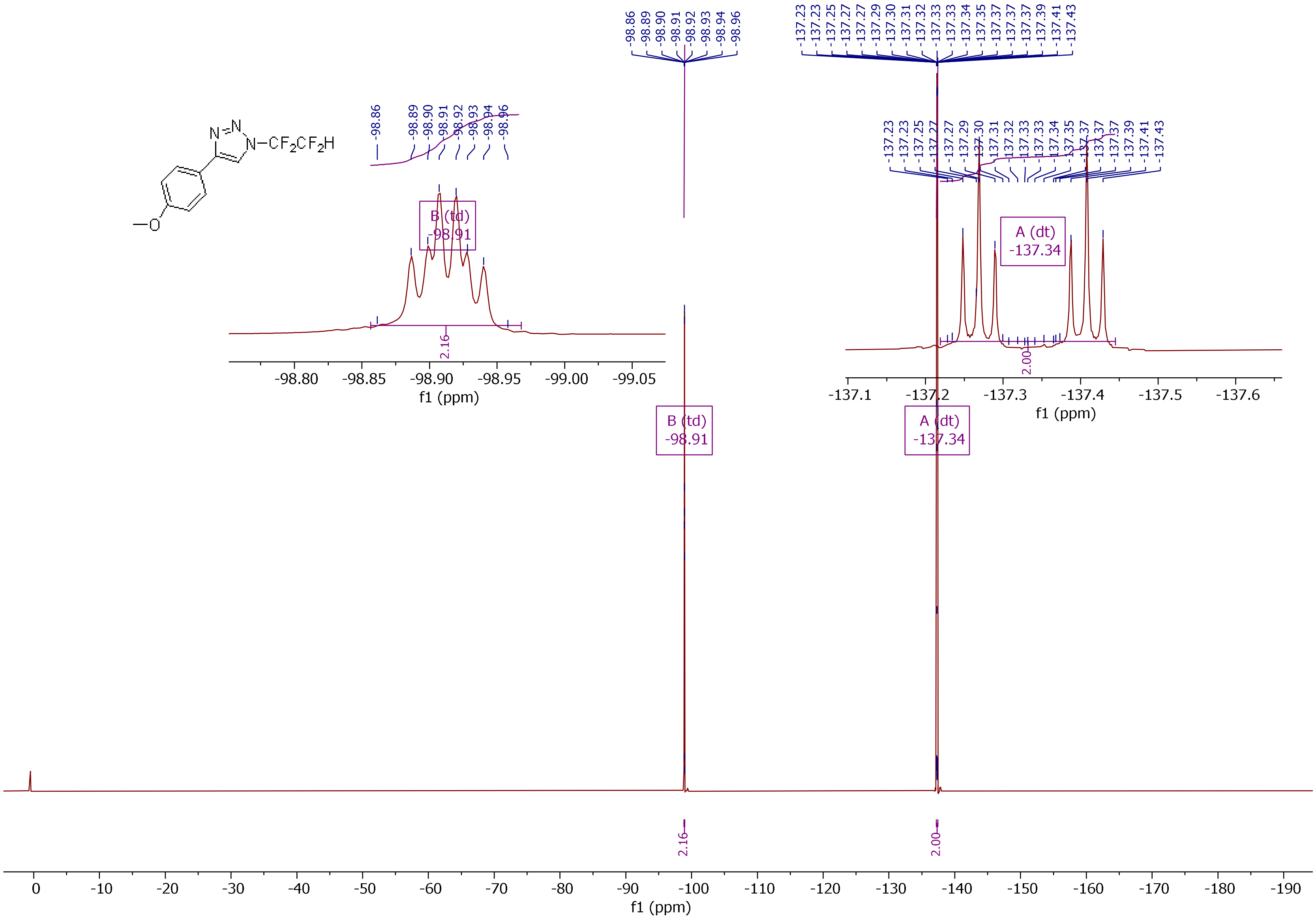

$^{13}C\{^1H\}$ NMR spectrum of **2d** ($CDCl_3$, 101 MHz)

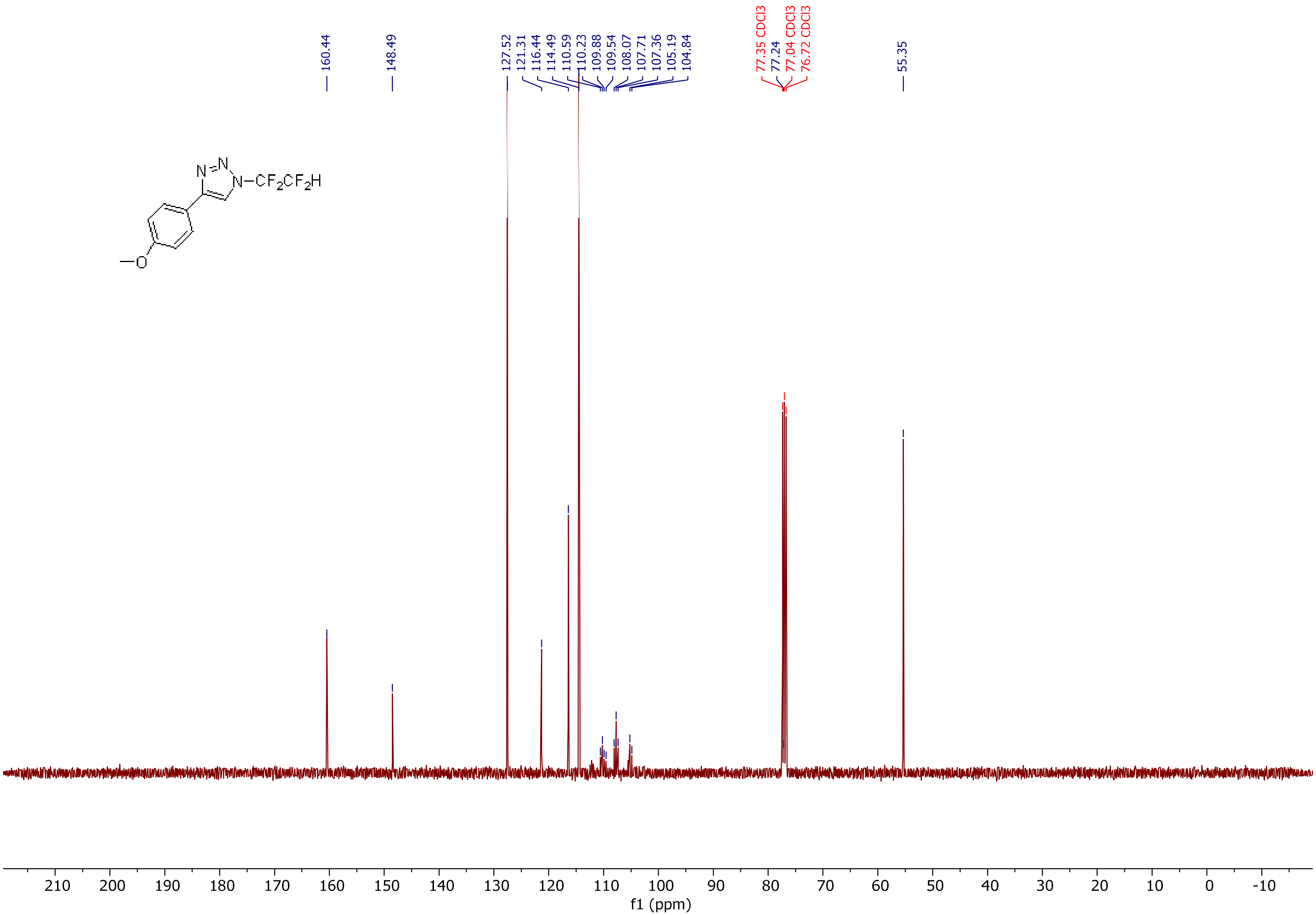

^{1}H NMR spectrum of **2e** ($CDCl_3$, 400 MHz)

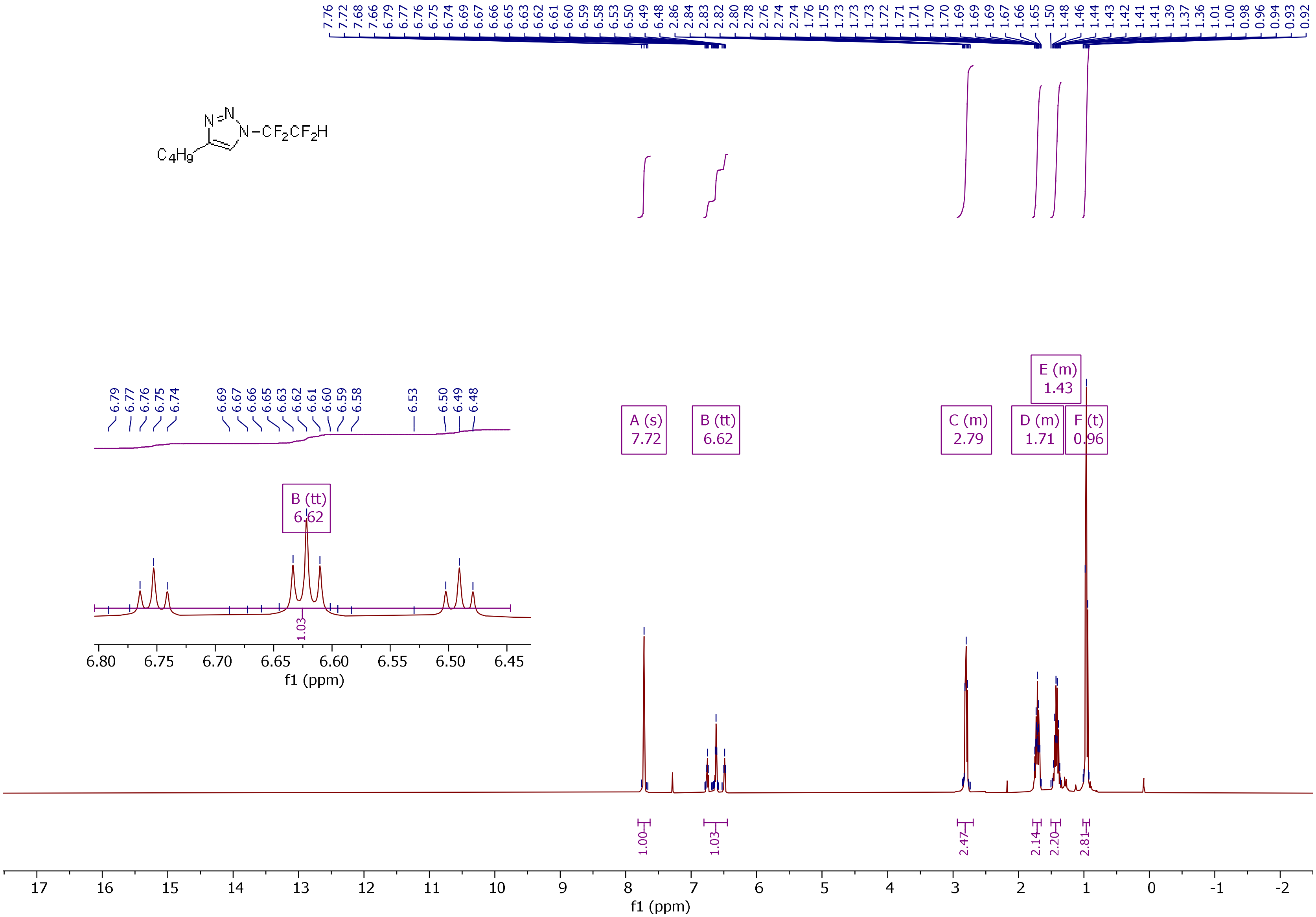

^{19}F NMR spectrum of **2e** ($CDCl_3$, 377 MHz)

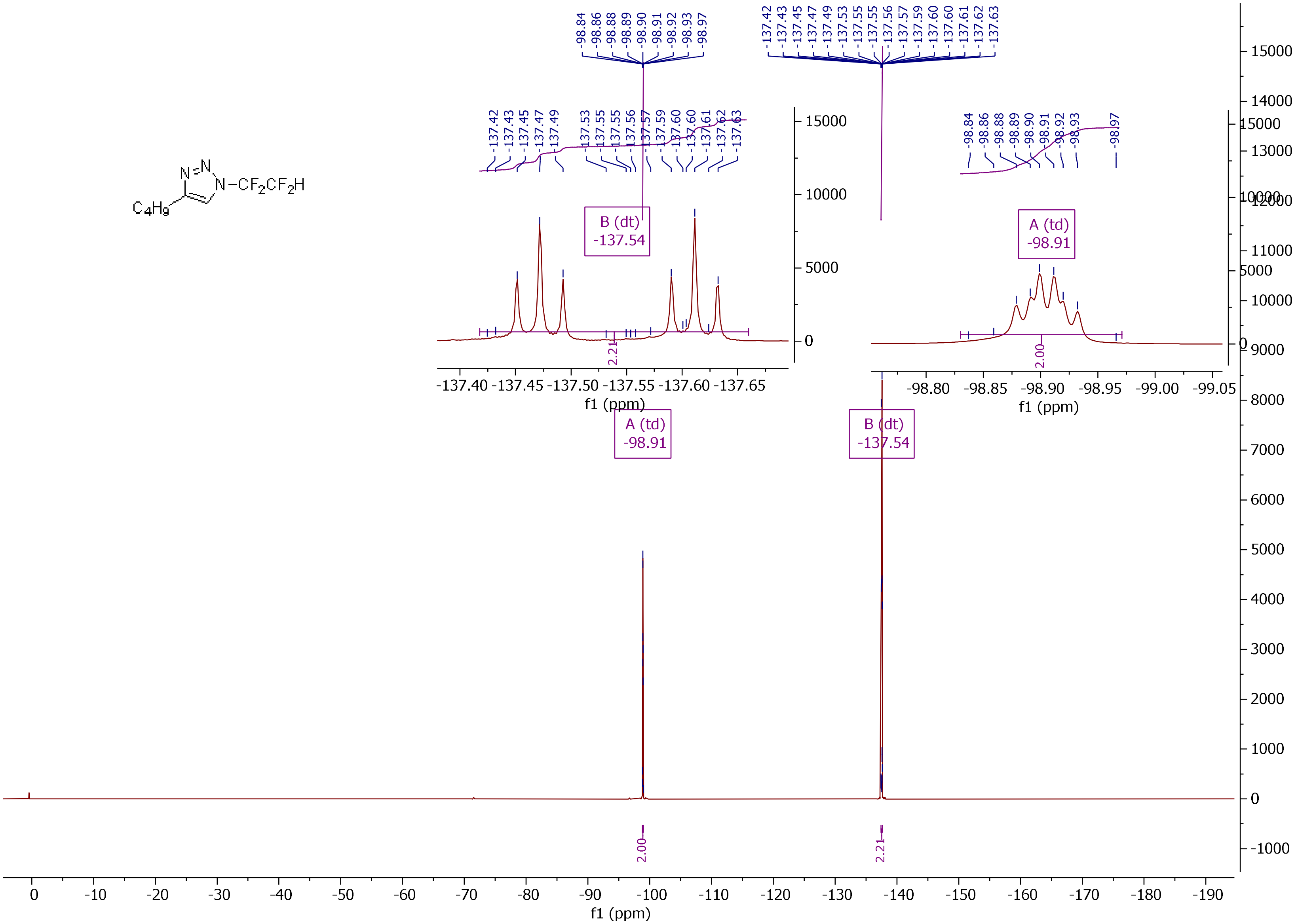

$^{13}C\{^1H\}$ NMR spectrum of **2e** ($CDCl_3$, 101 MHz)

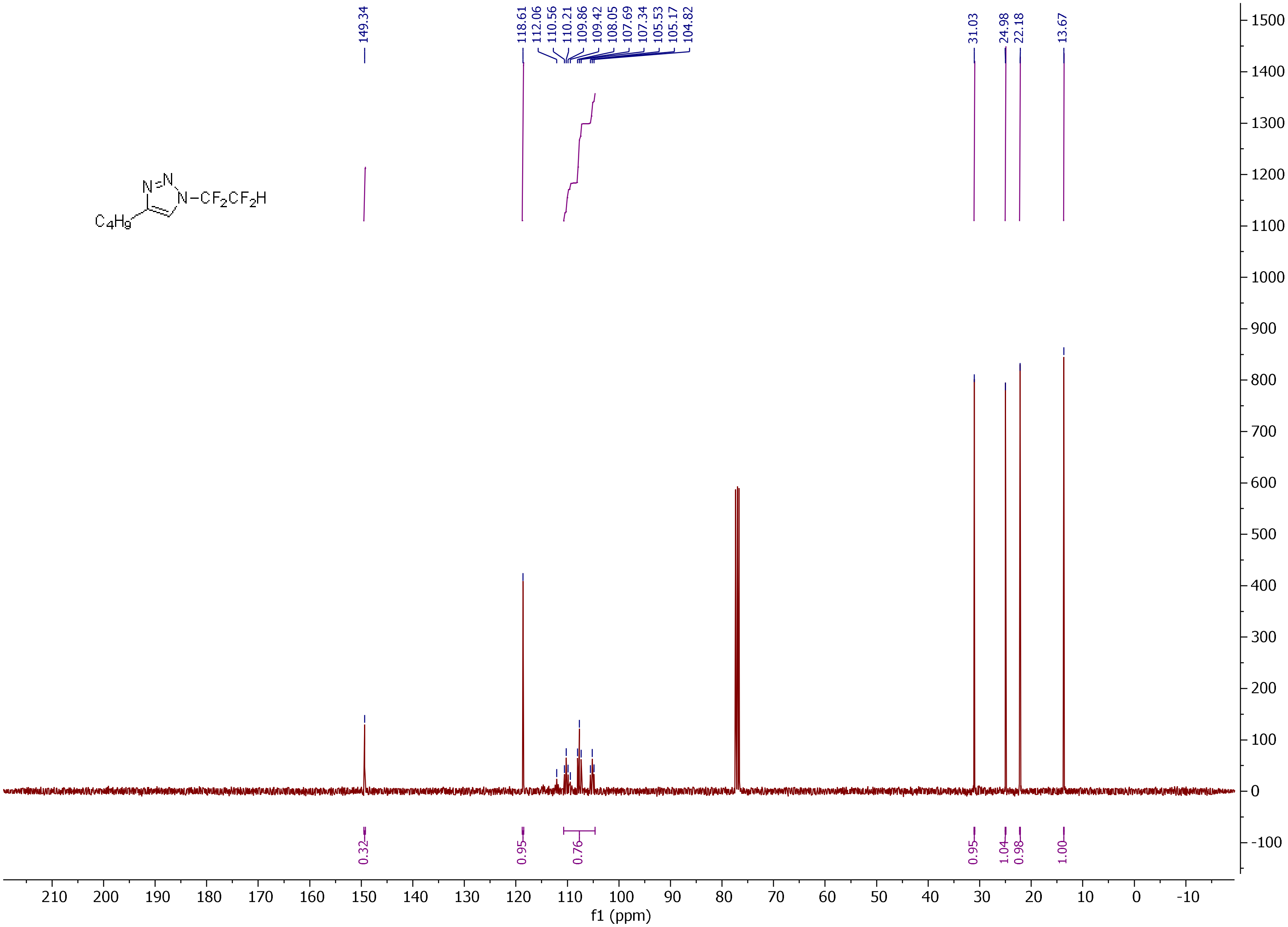

^{1}H NMR spectrum of **2f** ($CDCl_3$, 400 MHz)

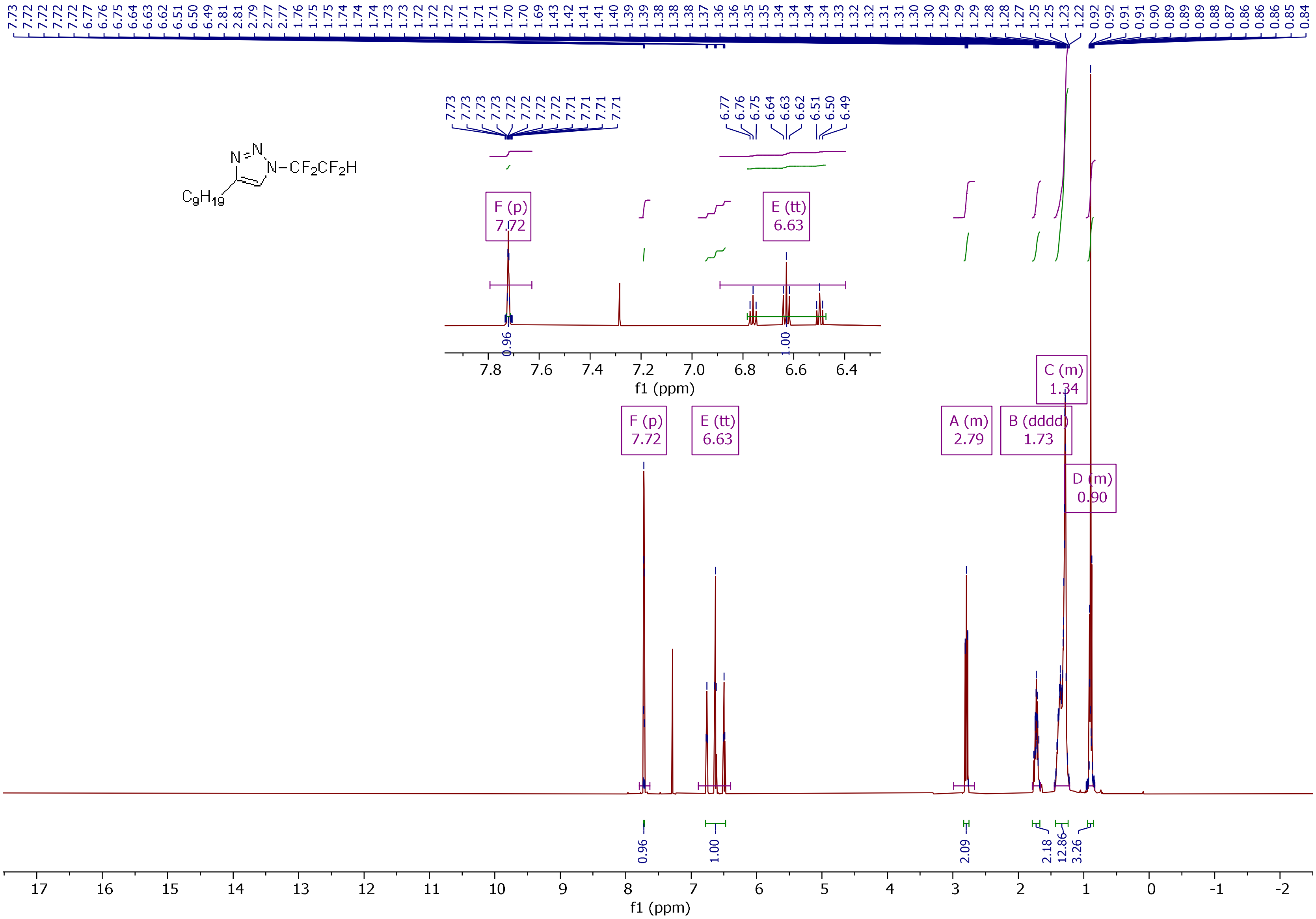

^{19}F NMR spectrum of **2f** ($CDCl_3$, 377 MHz)

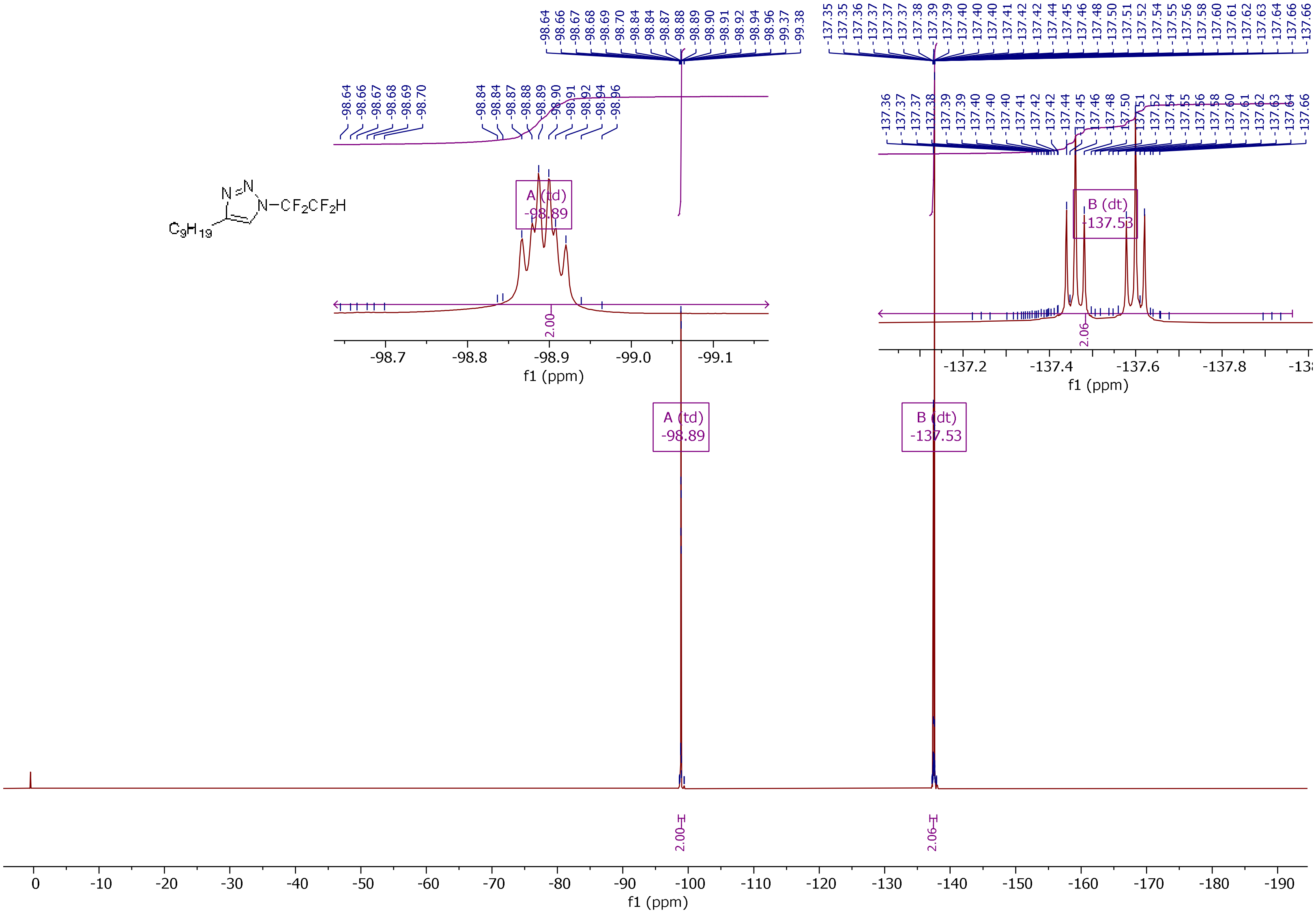

$^{13}C\{^1H\}$ NMR spectrum of **2f** ($CDCl_3$, 101 MHz)

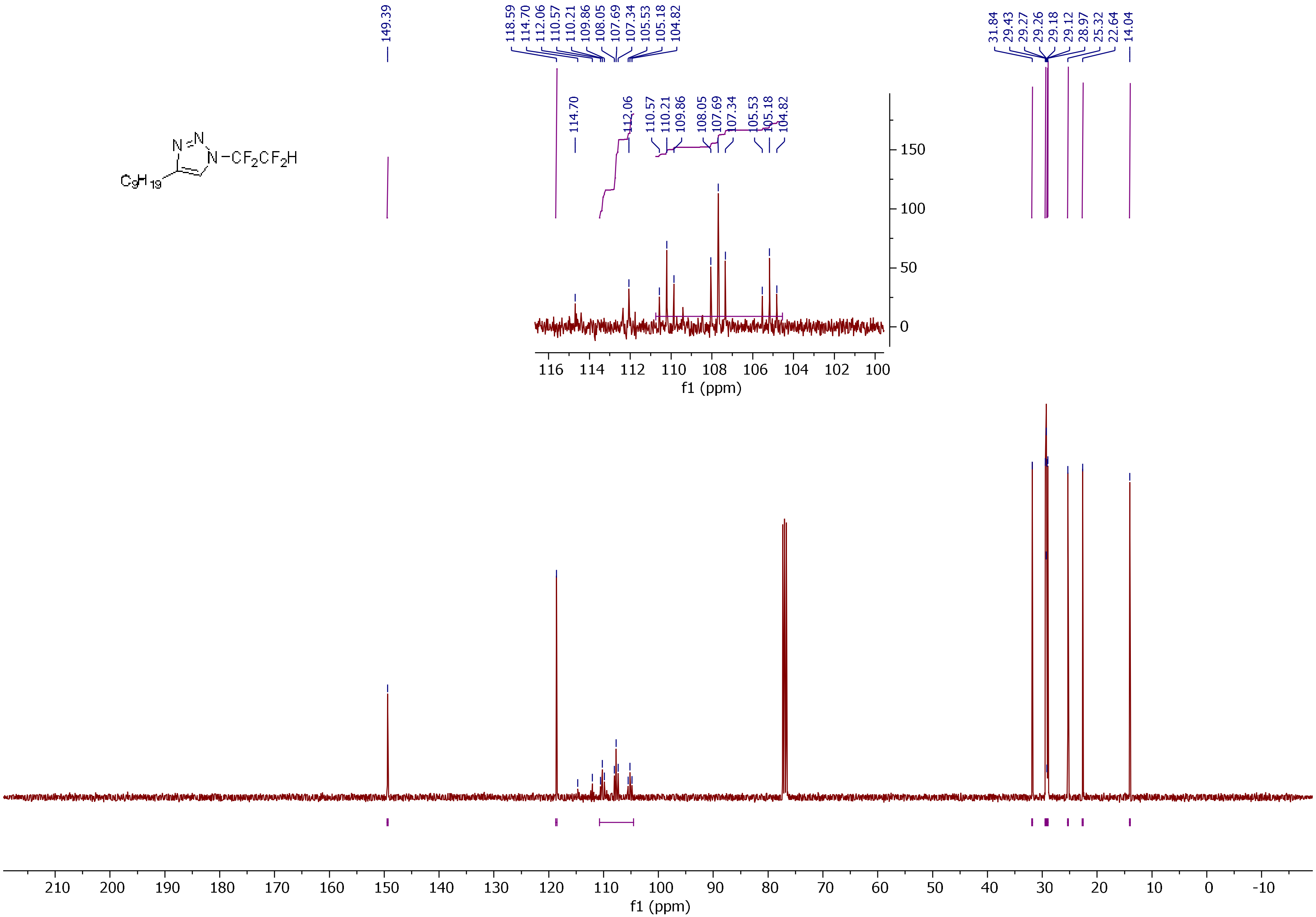

^{1}H NMR spectrum of **2g** ($CDCl_3$, 400 MHz)

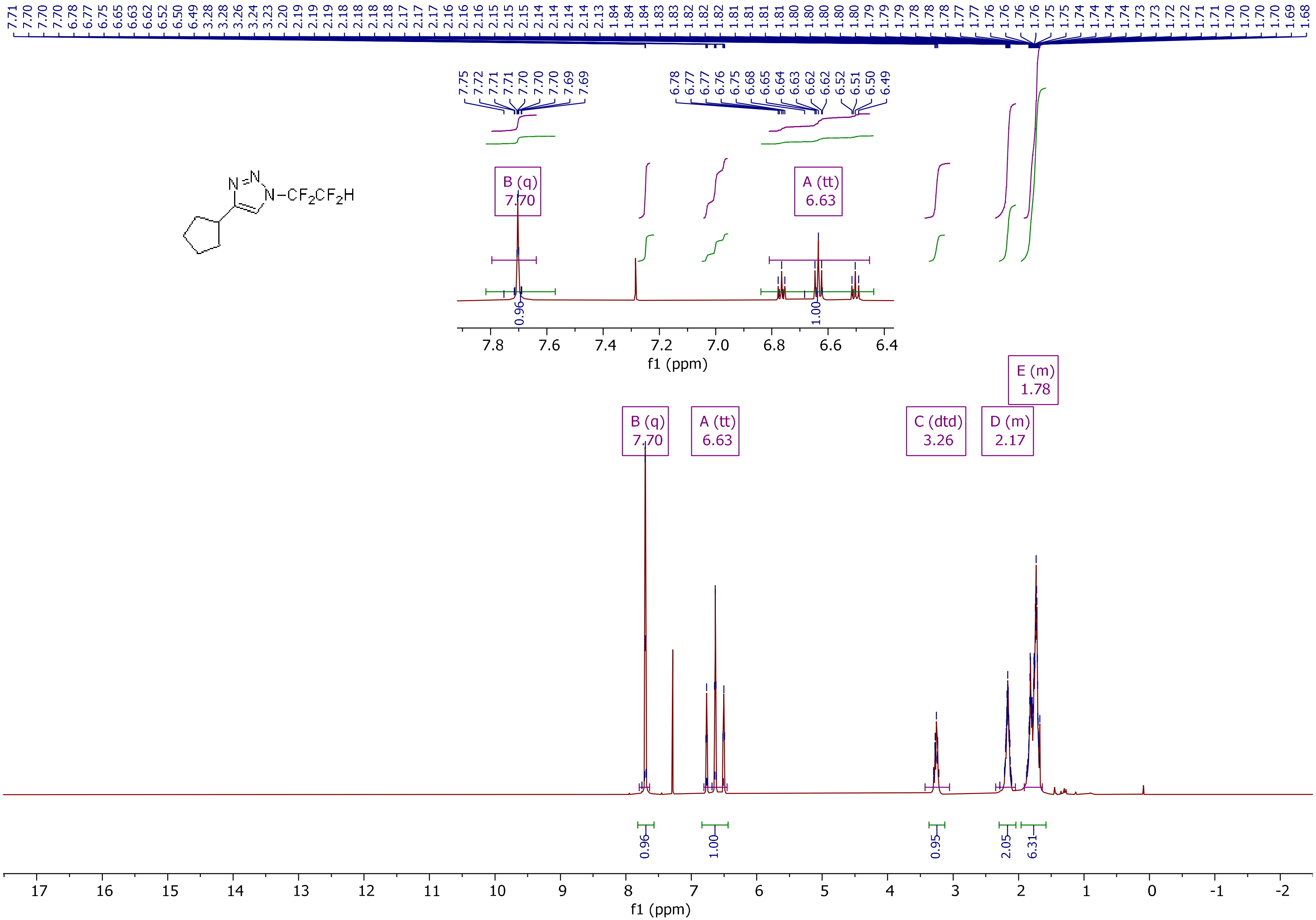

^{19}F NMR spectrum of **2g** ($CDCl_3$, 377 MHz)

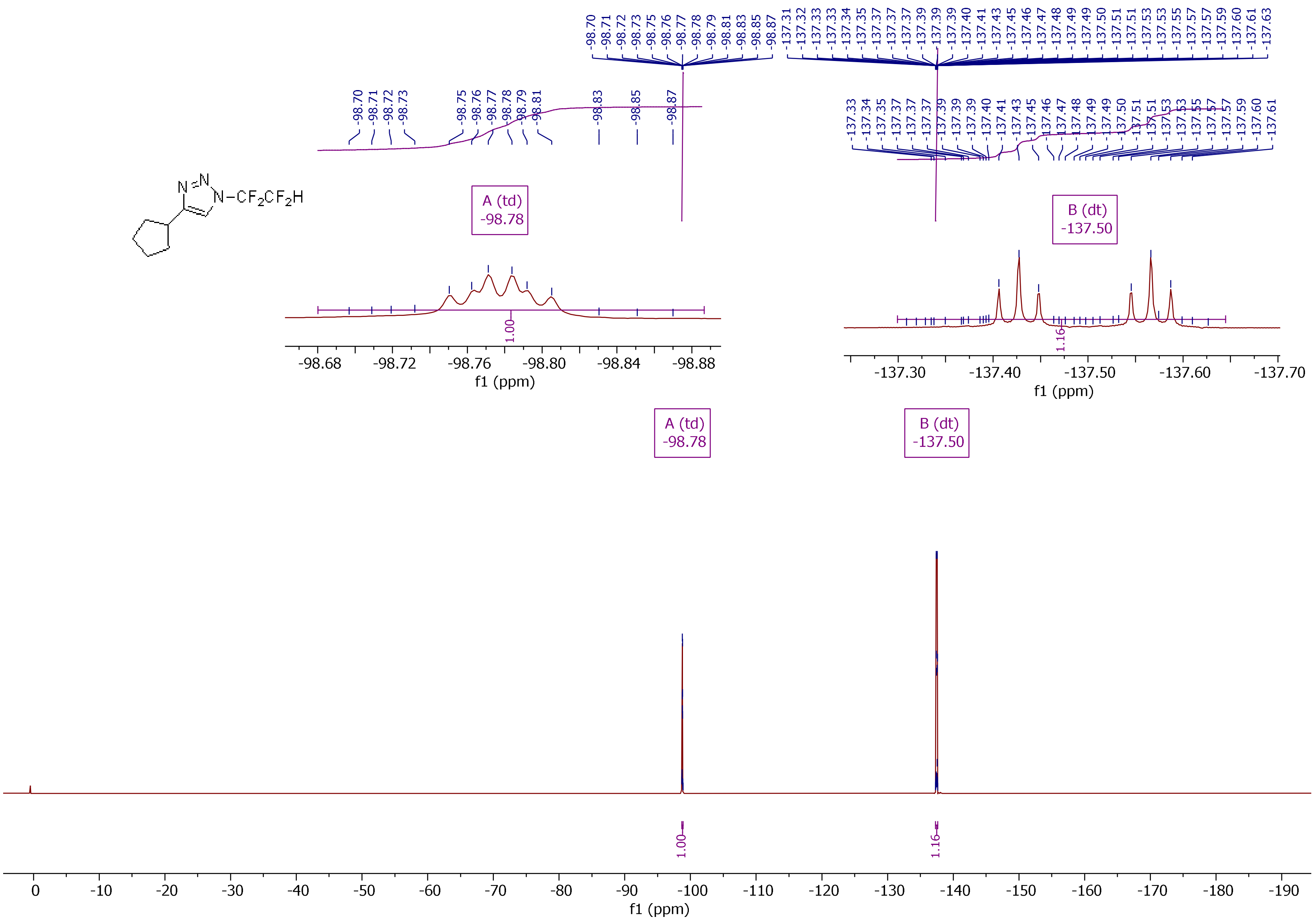

$^{13}C\{^1H\}$ NMR spectrum of **2g** ($CDCl_3$, 101 MHz)

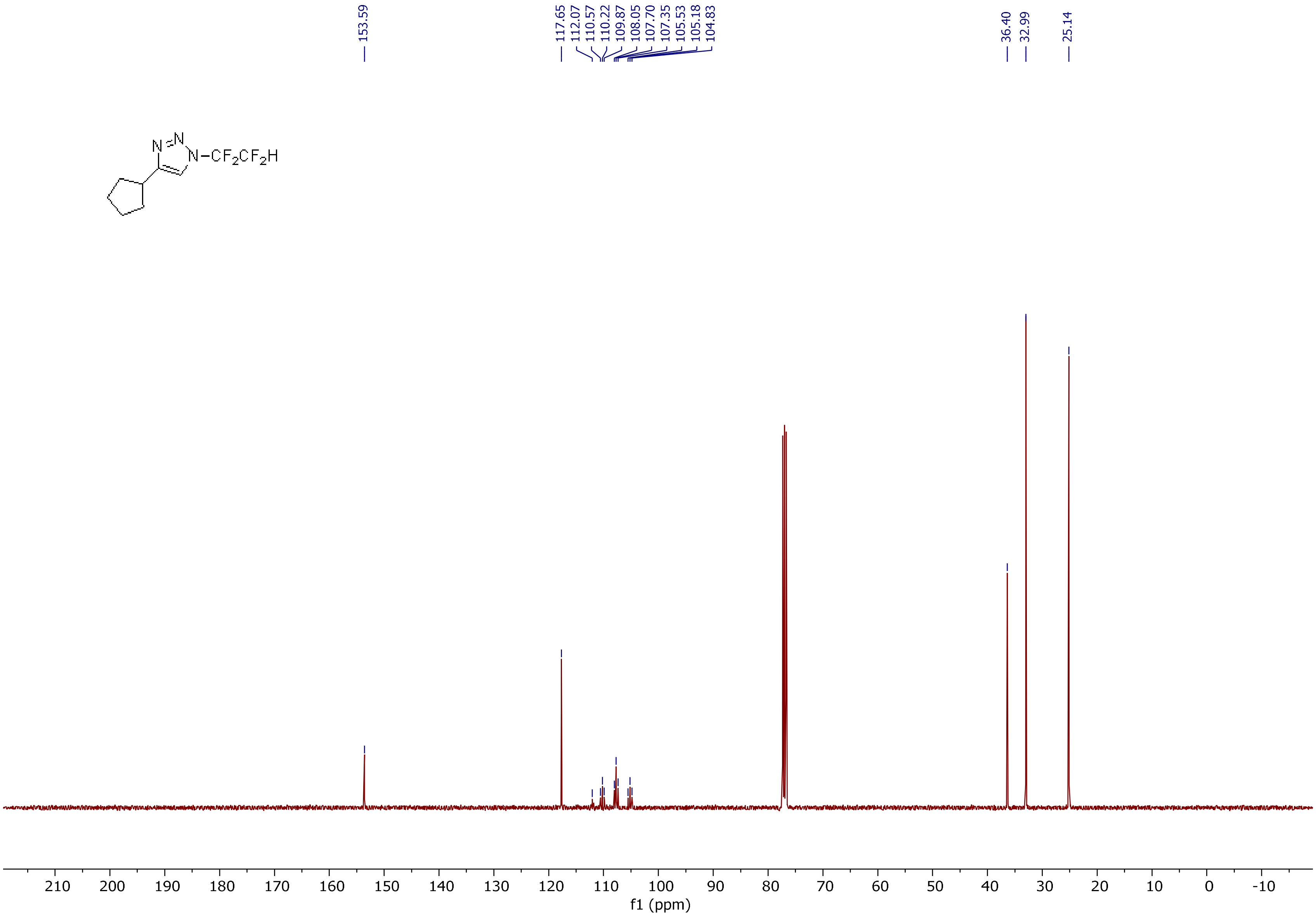

^{1}H NMR spectrum of **2h** ($CDCl_3$, 400 MHz)

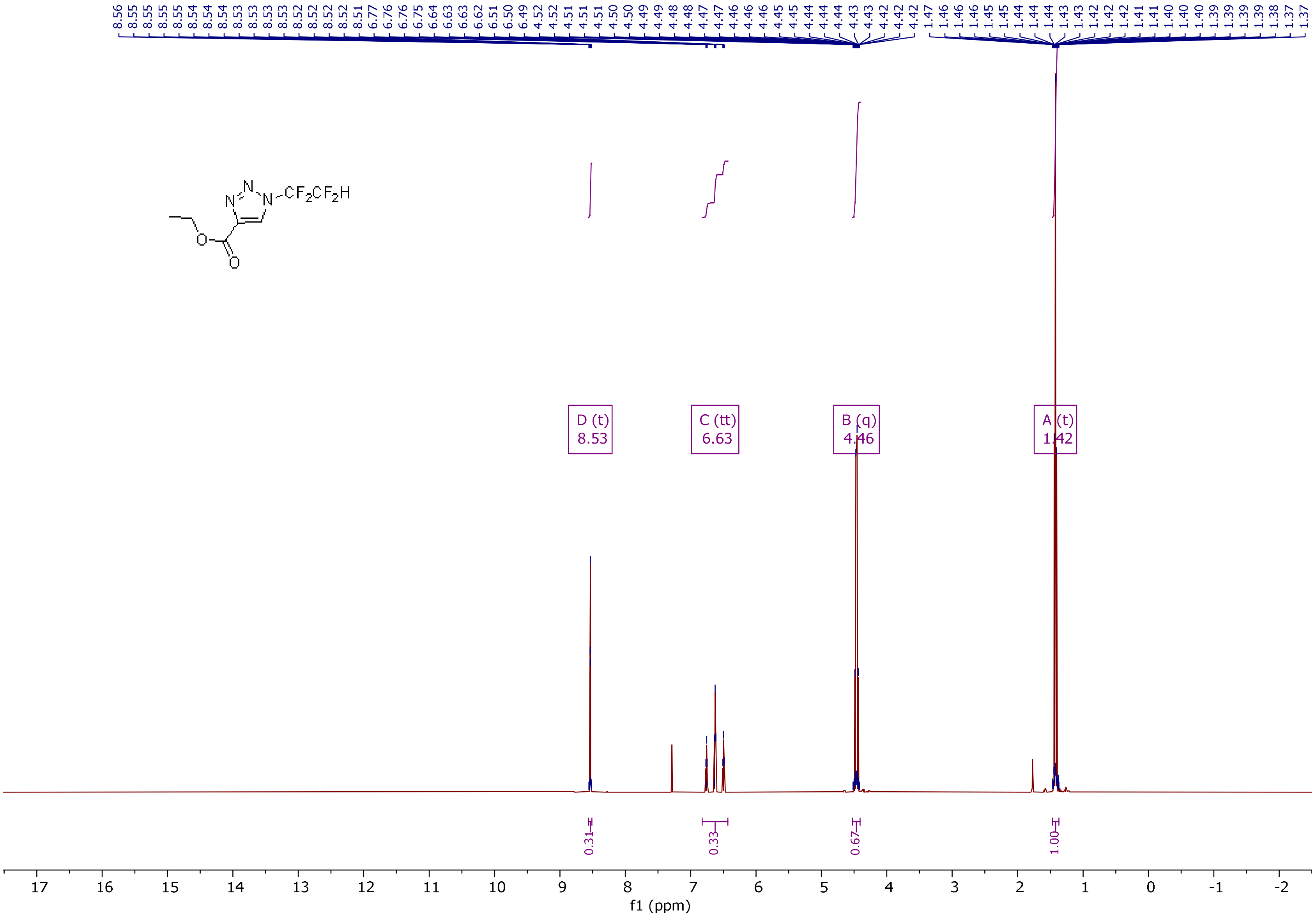

^{19}F NMR spectrum of **2h** ($CDCl_3$, 377 MHz)

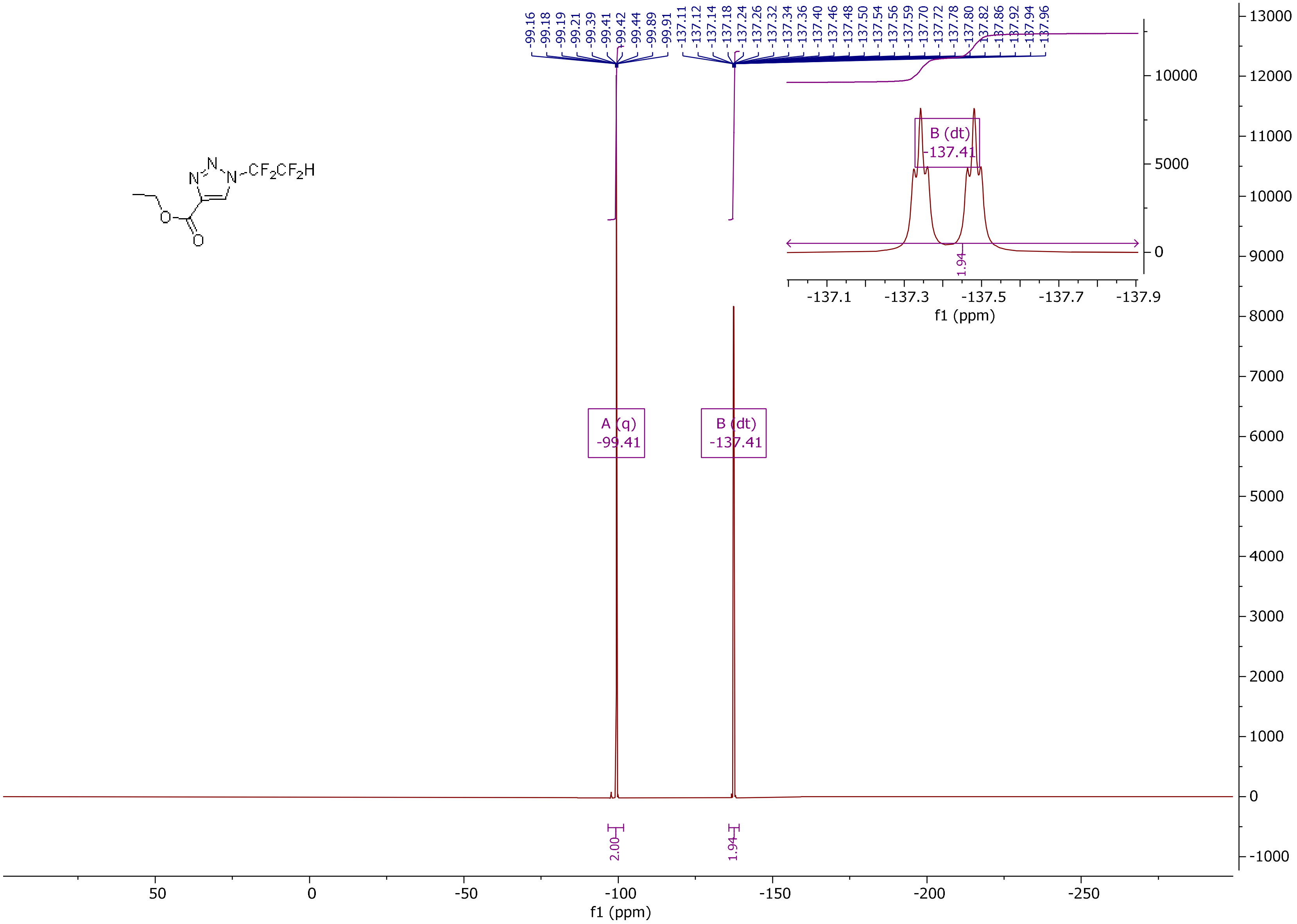

$^{13}C\{^{1}H\}$ NMR spectrum of **2h** ($CDCl_3$, 101 MHz)

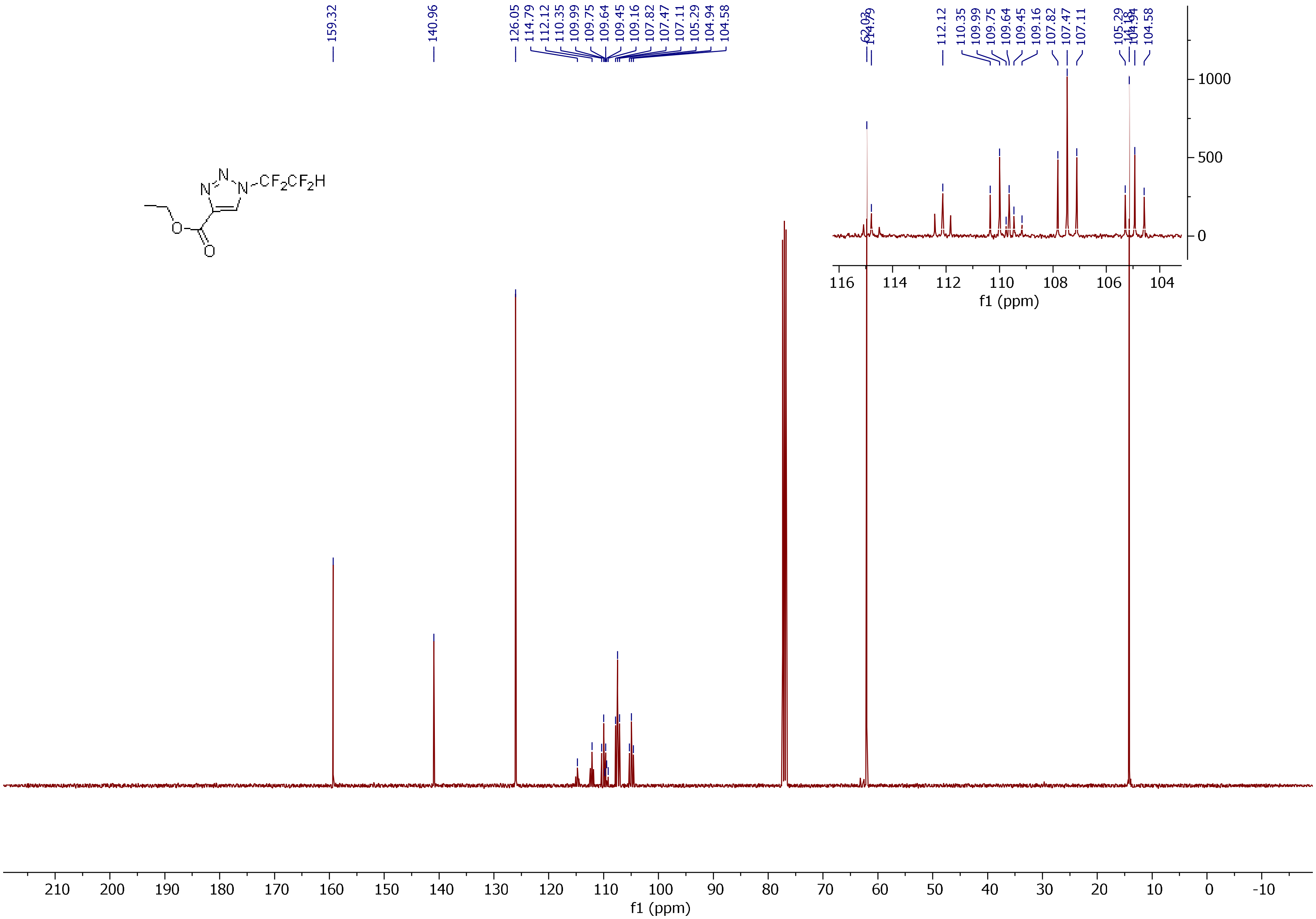

^{1}H NMR spectrum of **2i** ($CDCl_3$, 400 MHz)

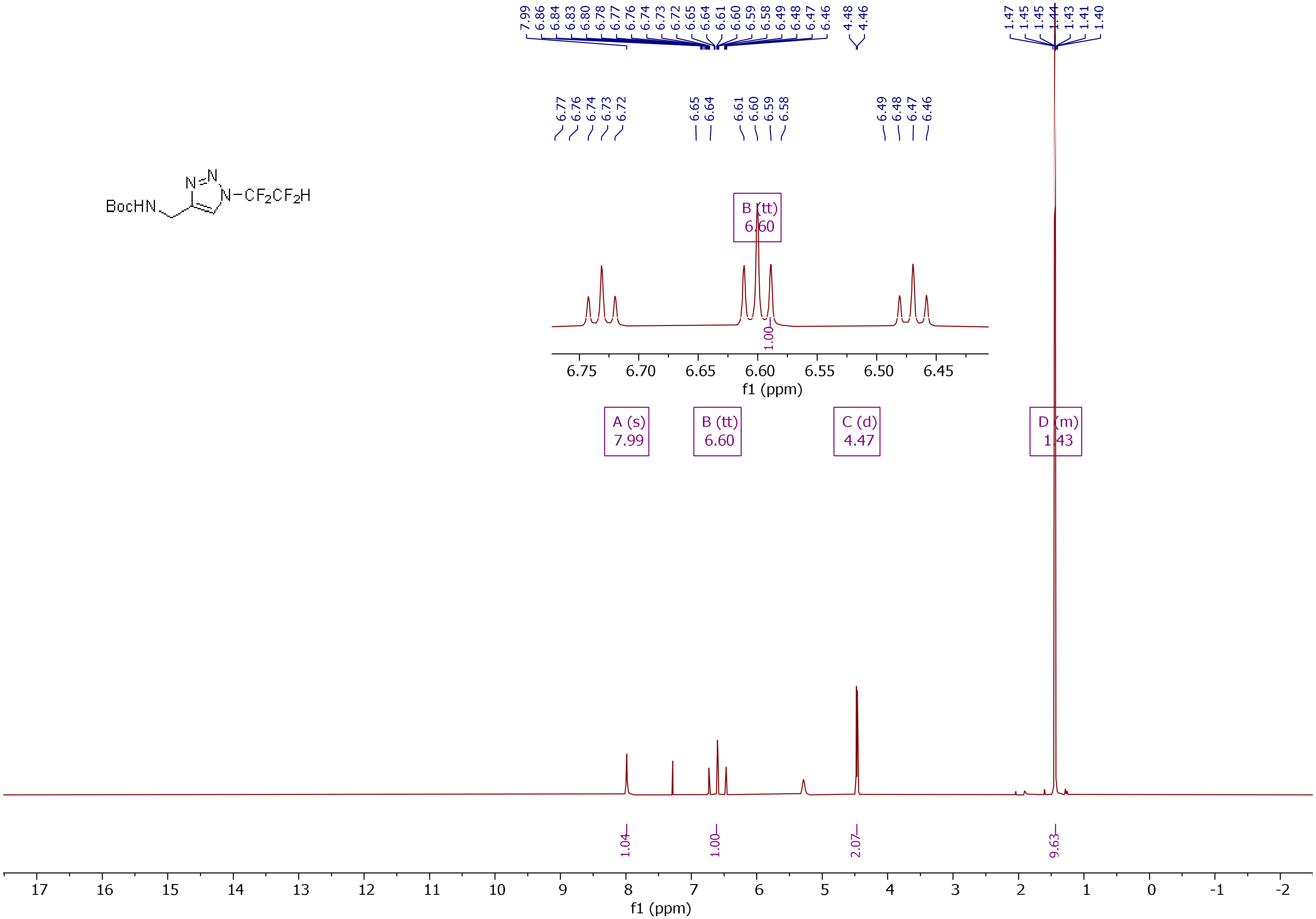

^{19}F NMR spectrum of **2i** ($CDCl_3$, 377 MHz)

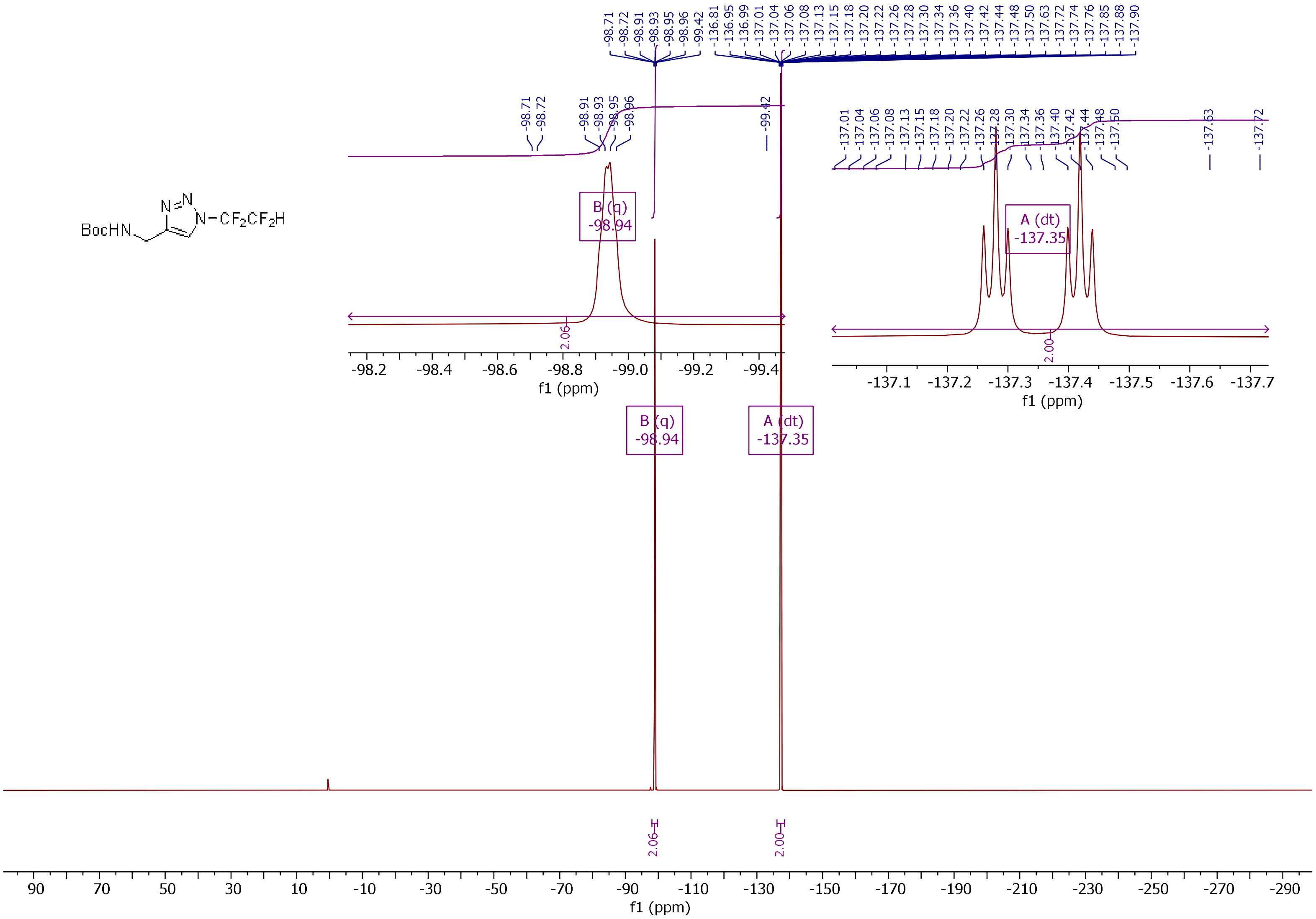

$^{13}C\{^{1}H\}$ NMR spectrum of **2i** ($CDCl_3$, 101 MHz)

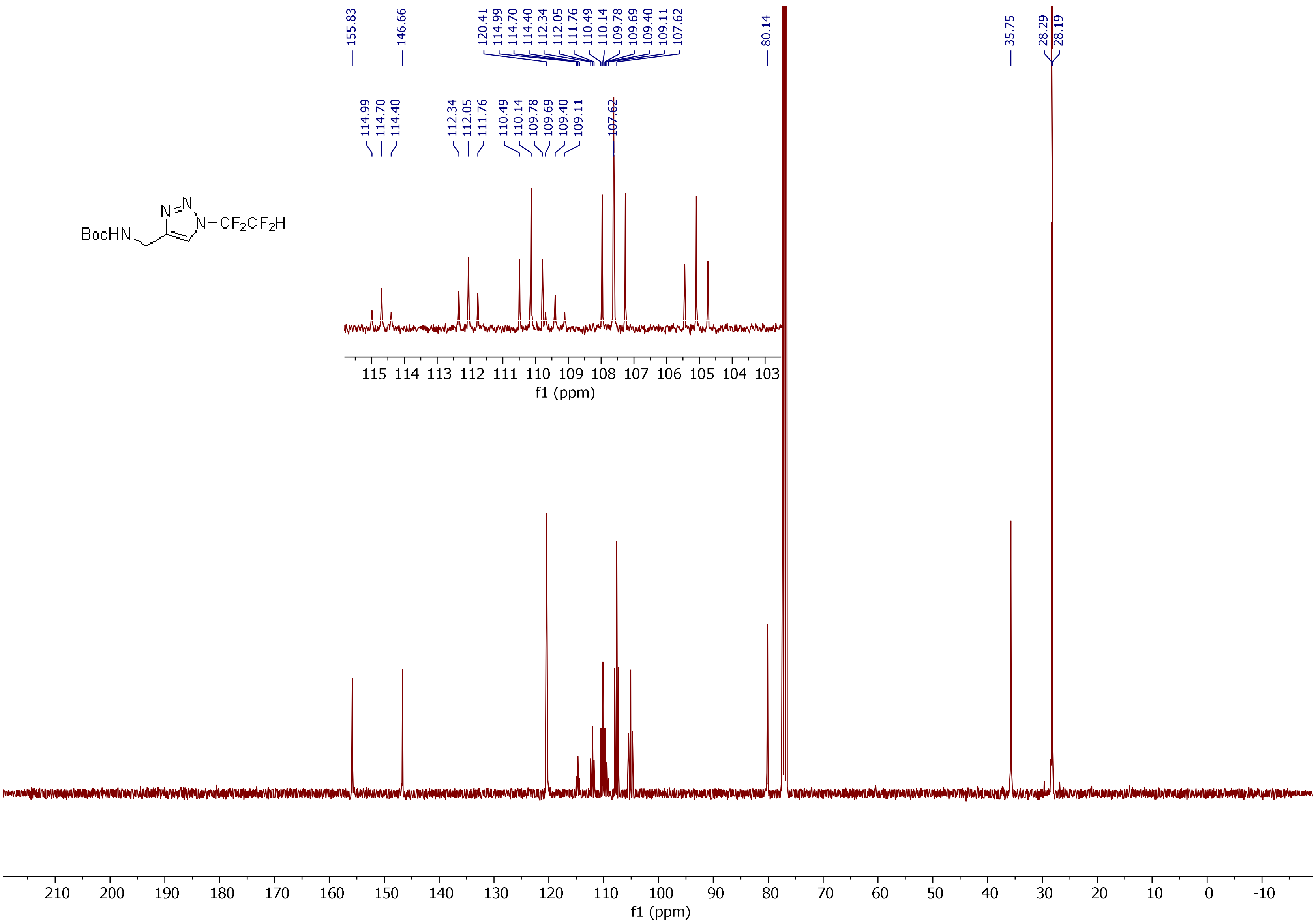

^{1}H NMR spectrum of **2j** (DMSO-d6, 300 MHz)

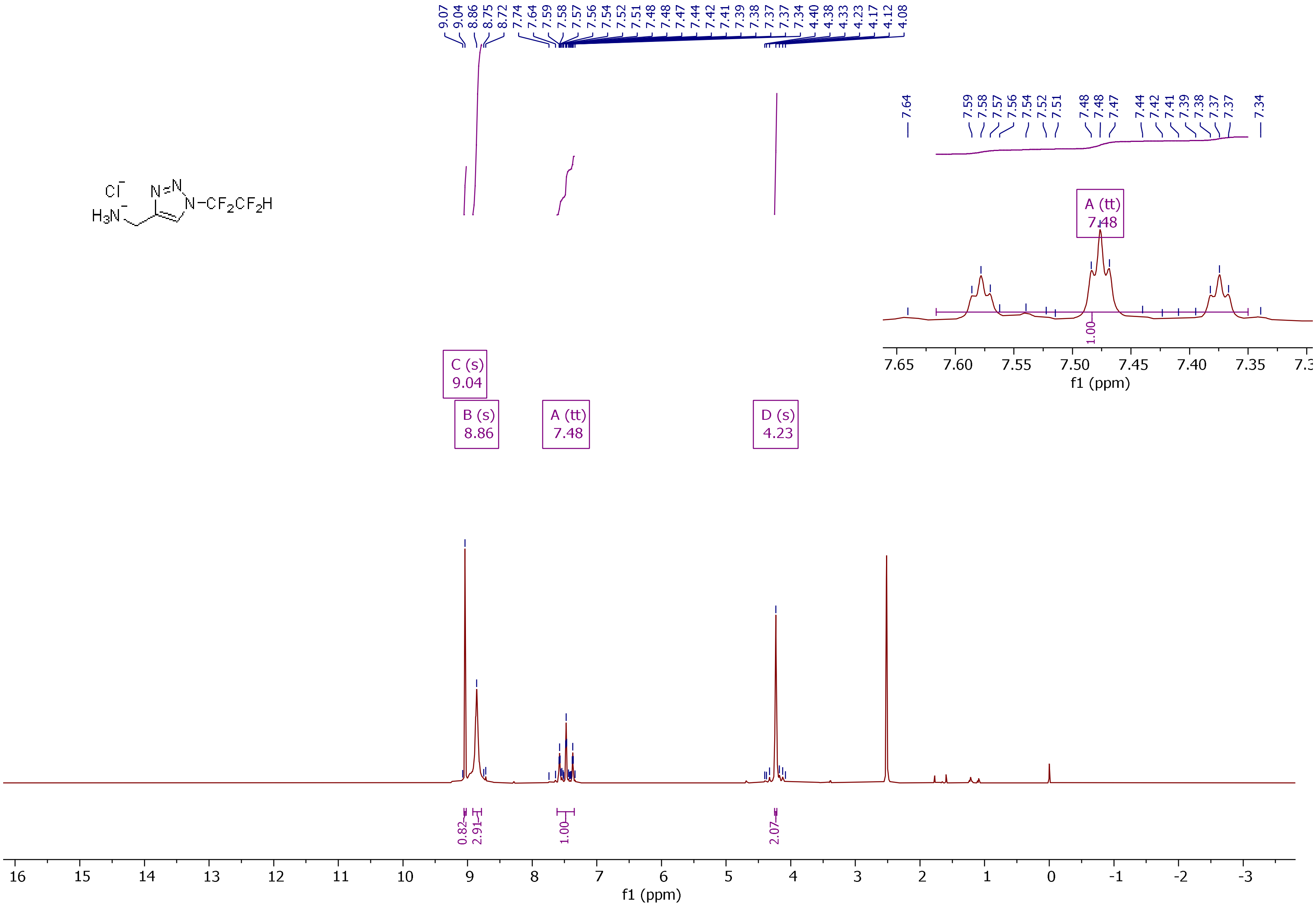

^{19}F NMR spectrum of **2j** (DMSO-d6, 282 MHz)

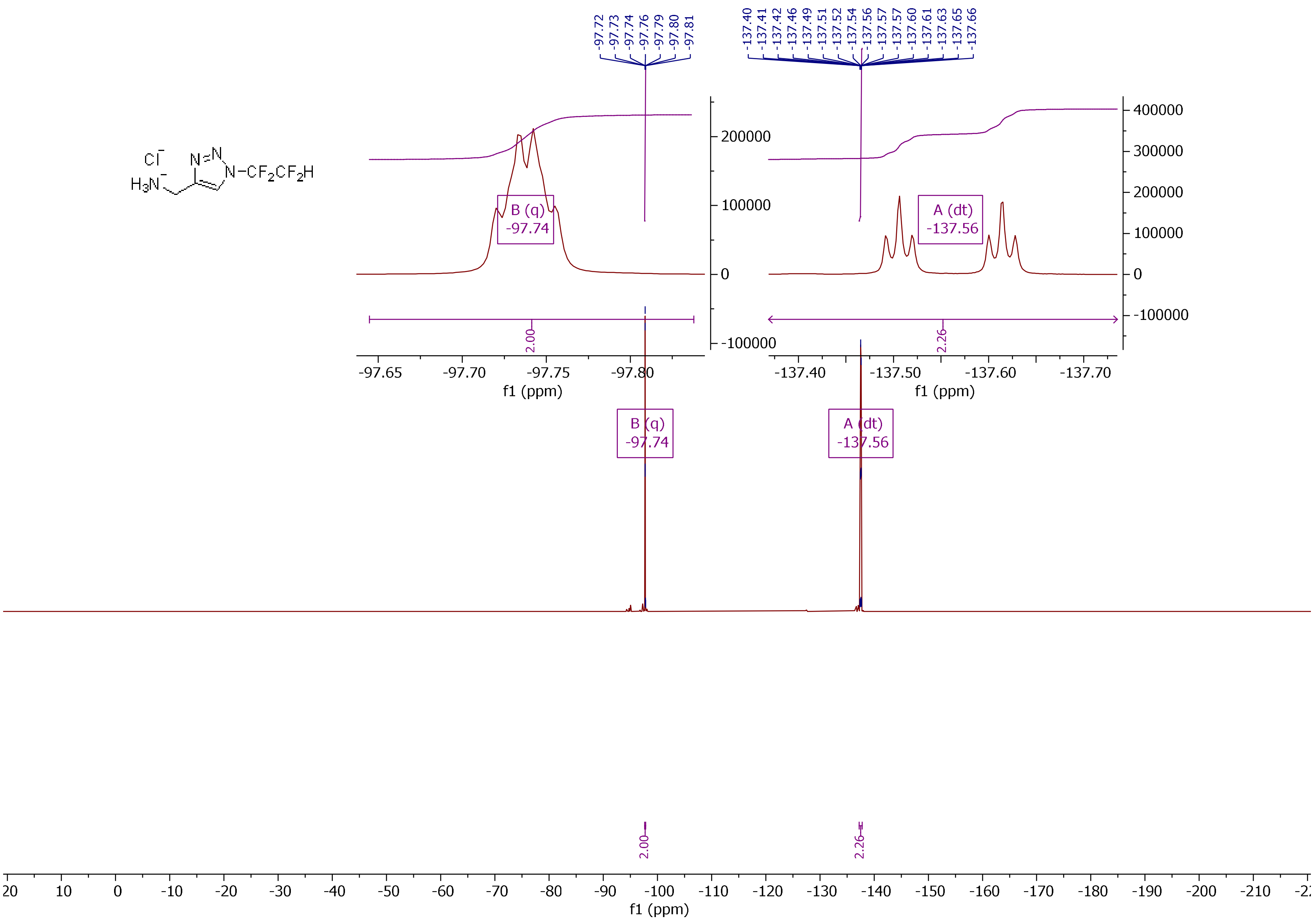

$^{13}C\{^1H\}$ NMR spectrum of **2j** (DMSO-d6, 101 MHz)

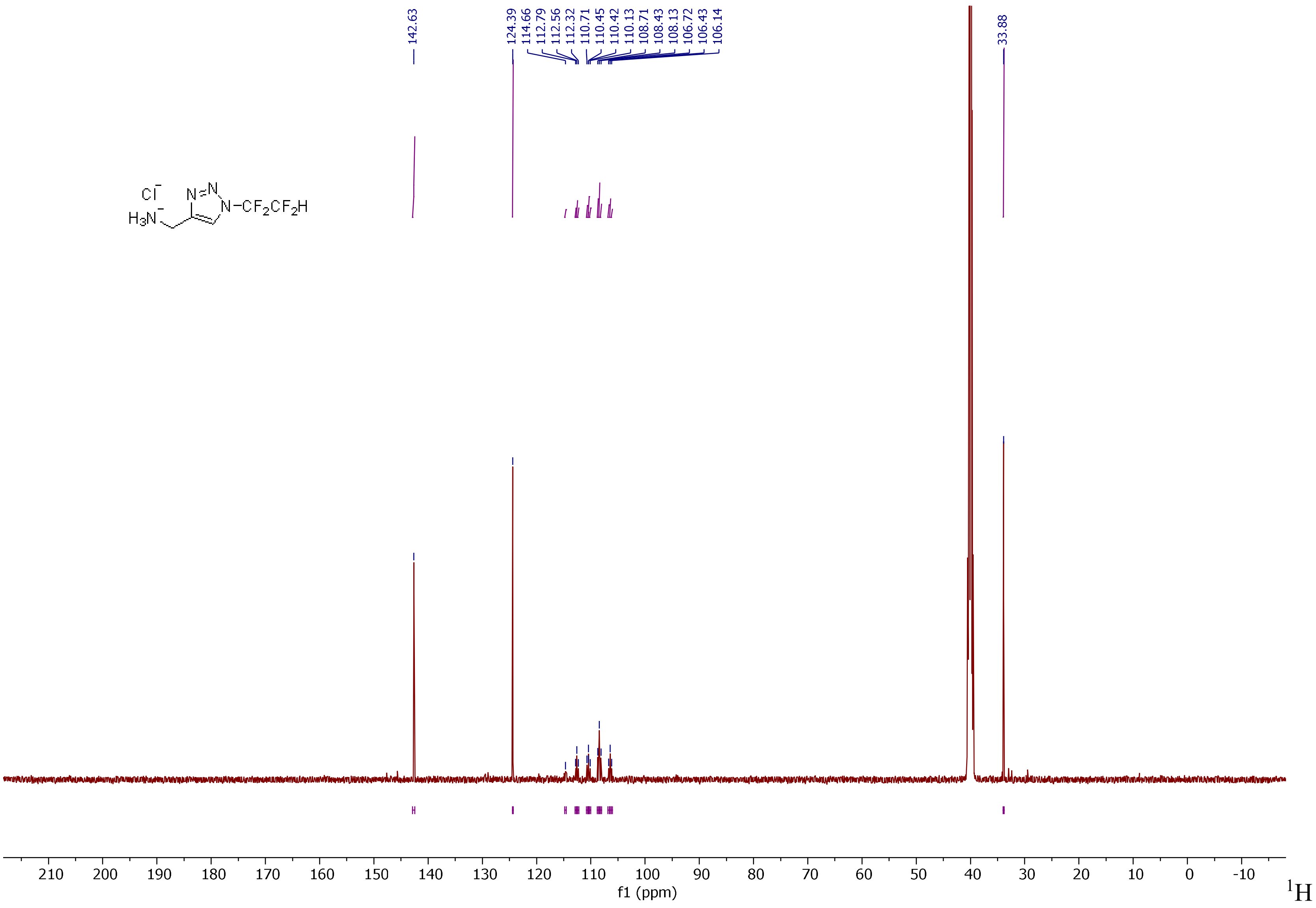

NMR spectrum of **2k** ($CDCl_3$, 400 MHz)

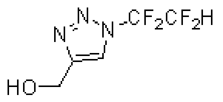

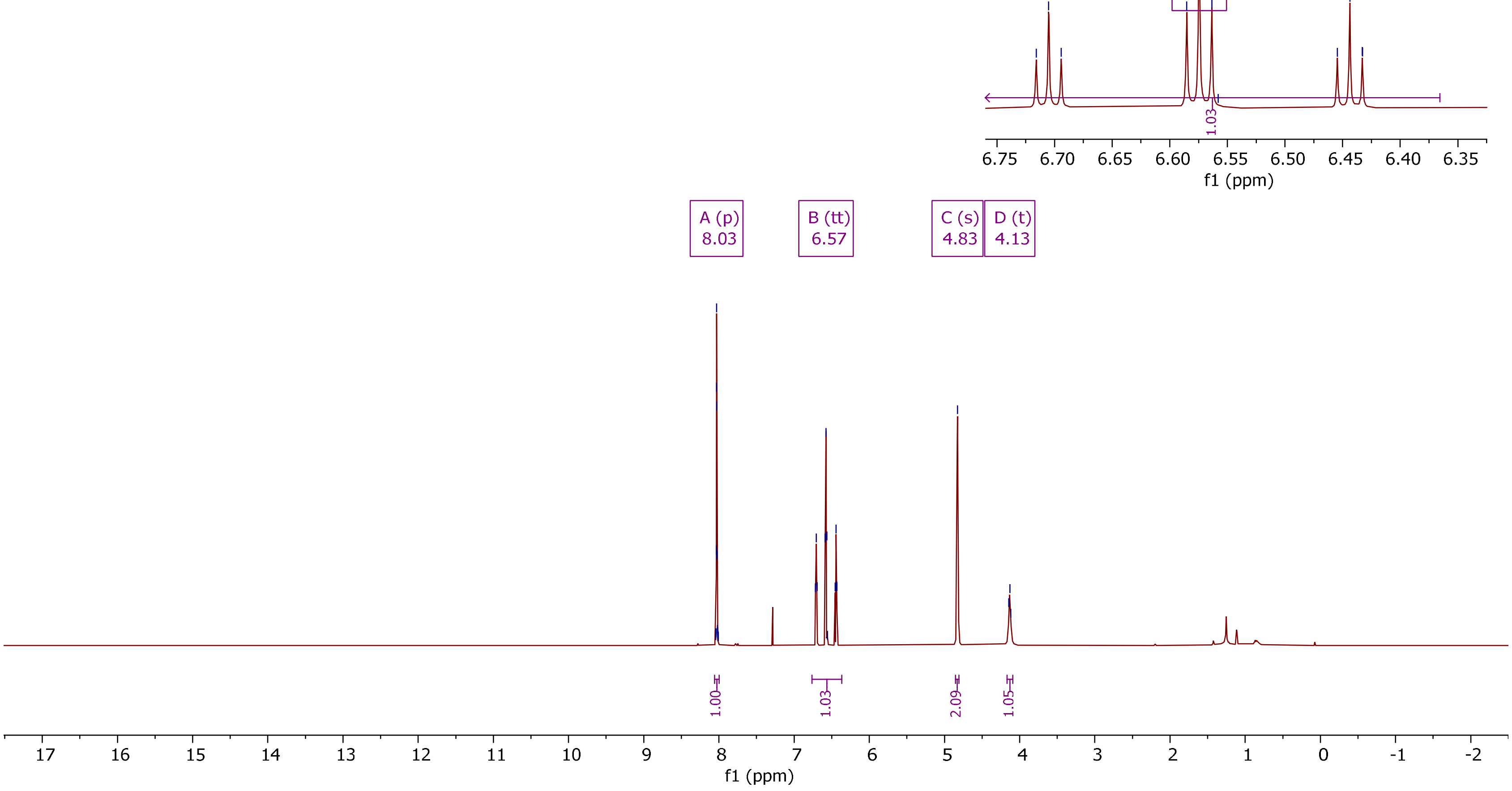

^{19}F NMR spectrum of **2k** ($CDCl_3$, 377 MHz)

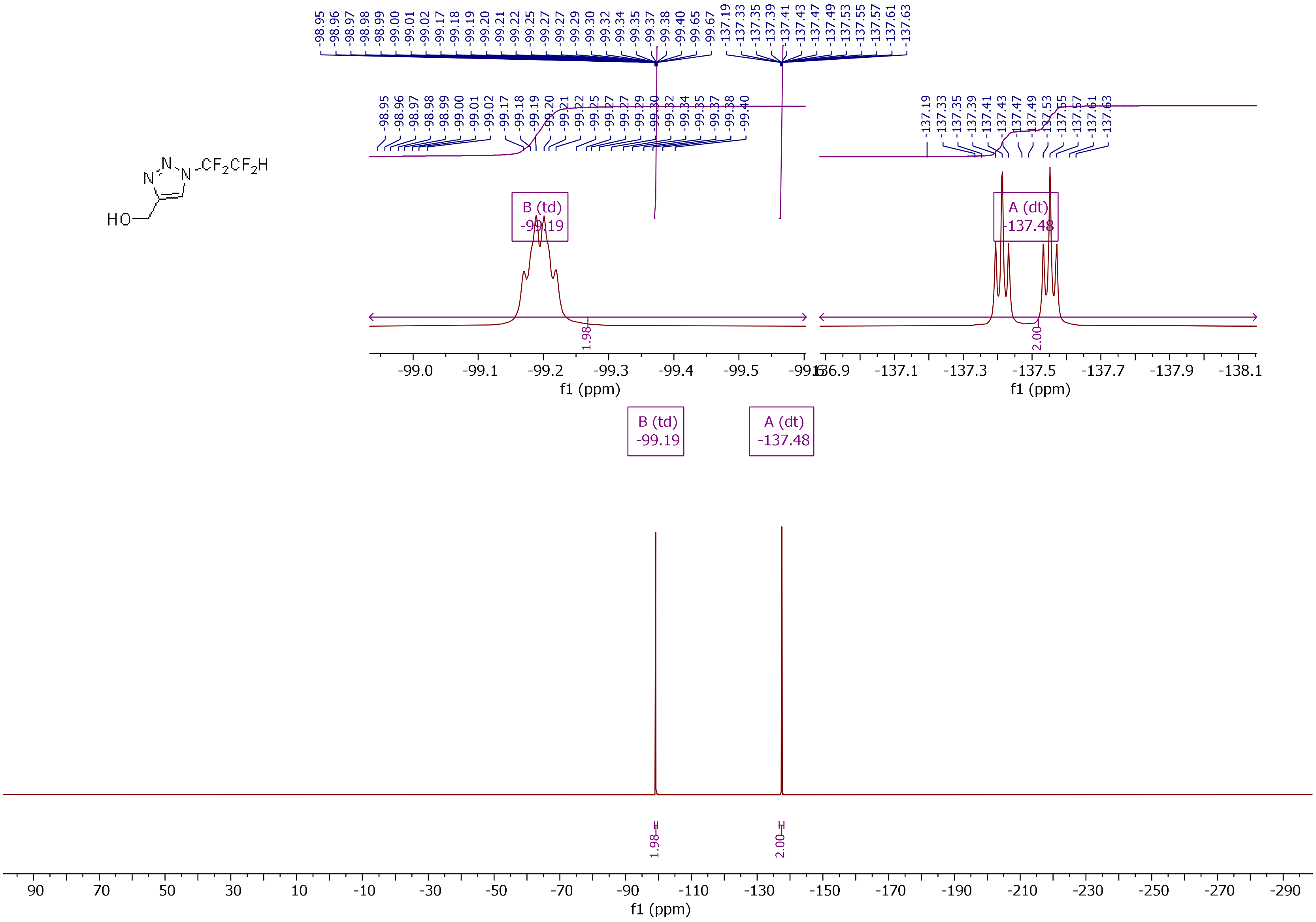

$^{13}C\{^{1}H\}$ NMR spectrum of **2k** ($CDCl_3$, 101 MHz)

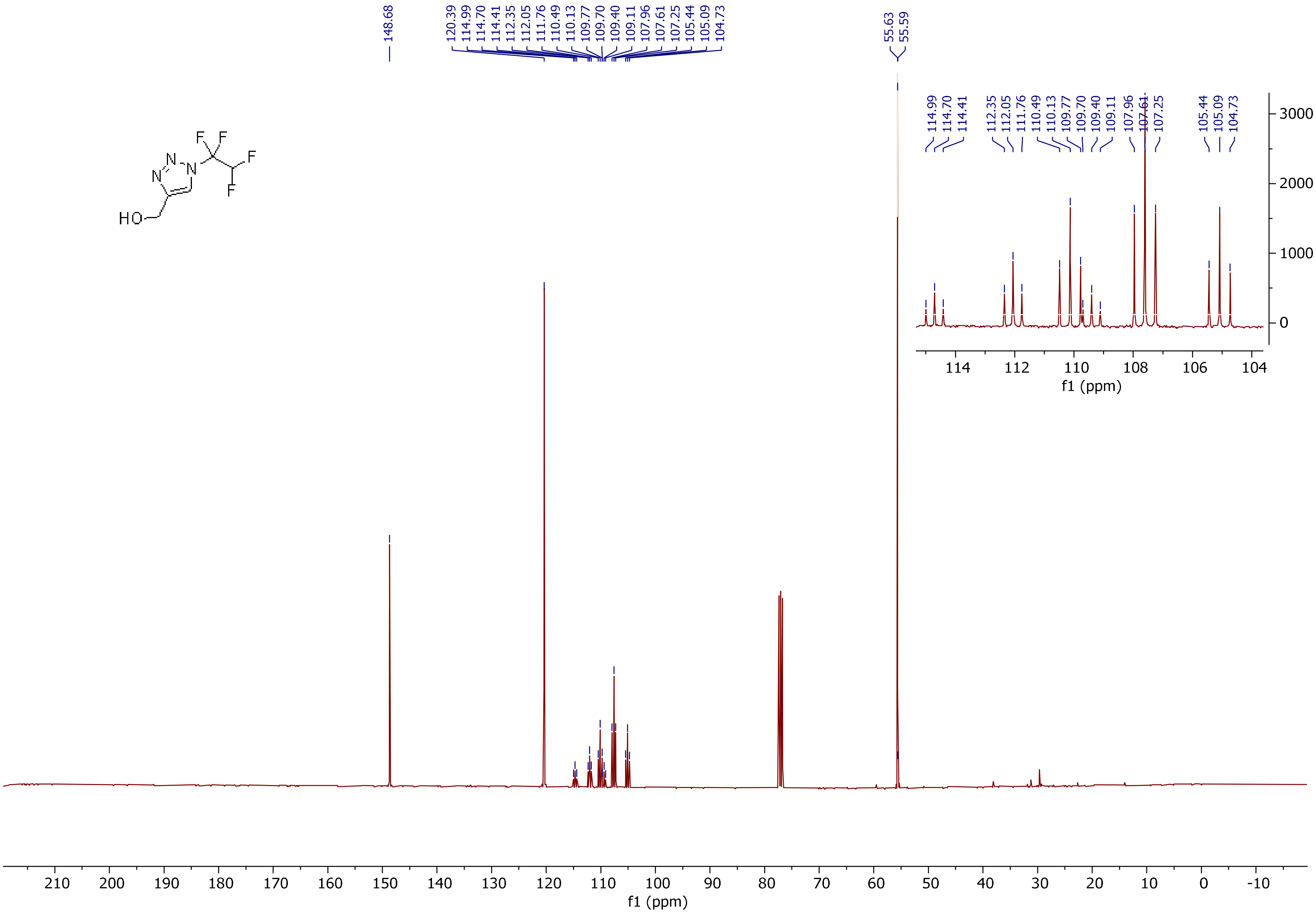

^{1}H NMR spectrum of **2l** ($CDCl_3$, 400 MHz)

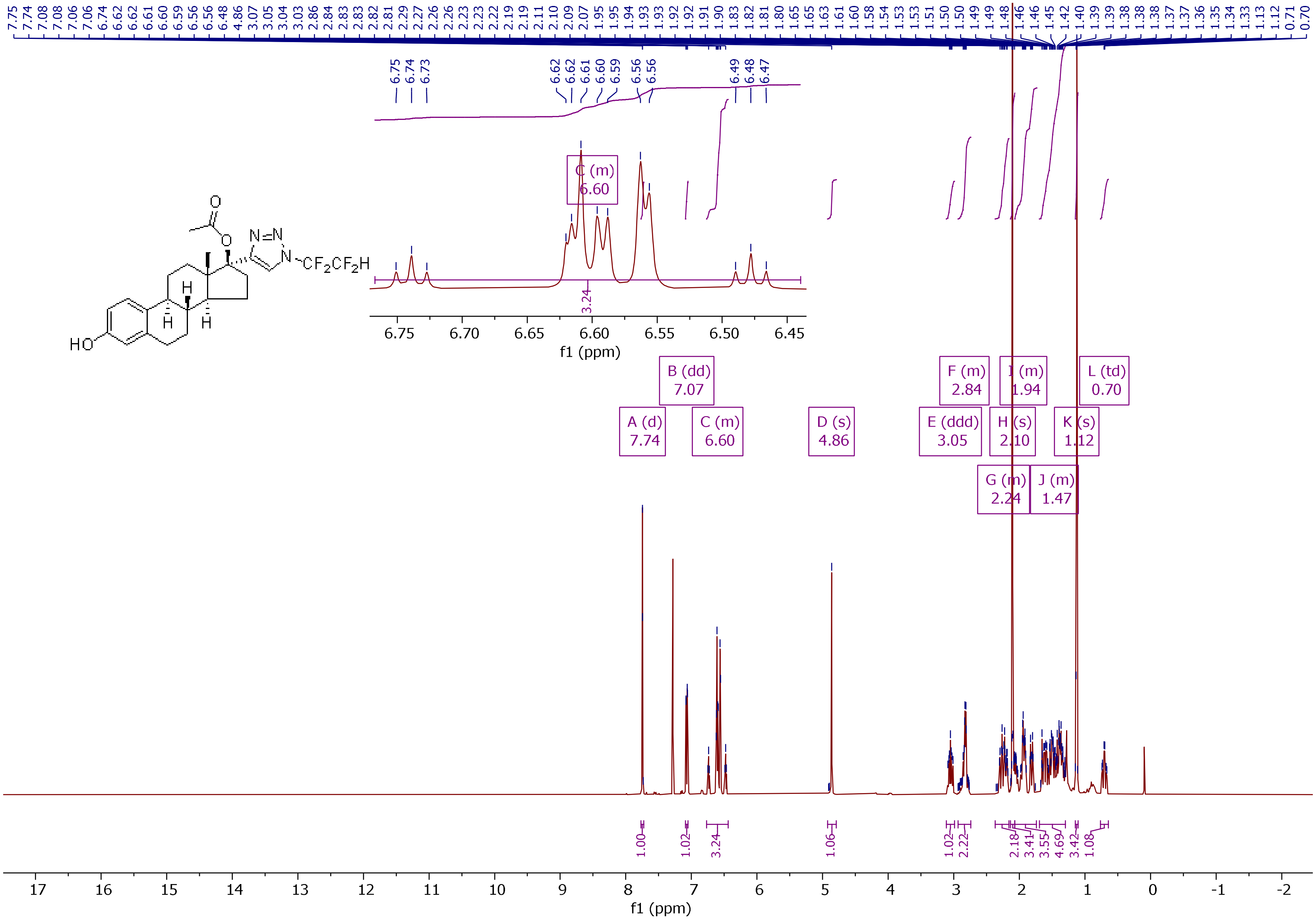

^{19}F NMR spectrum of **2l** ($CDCl_3$, 377 MHz)

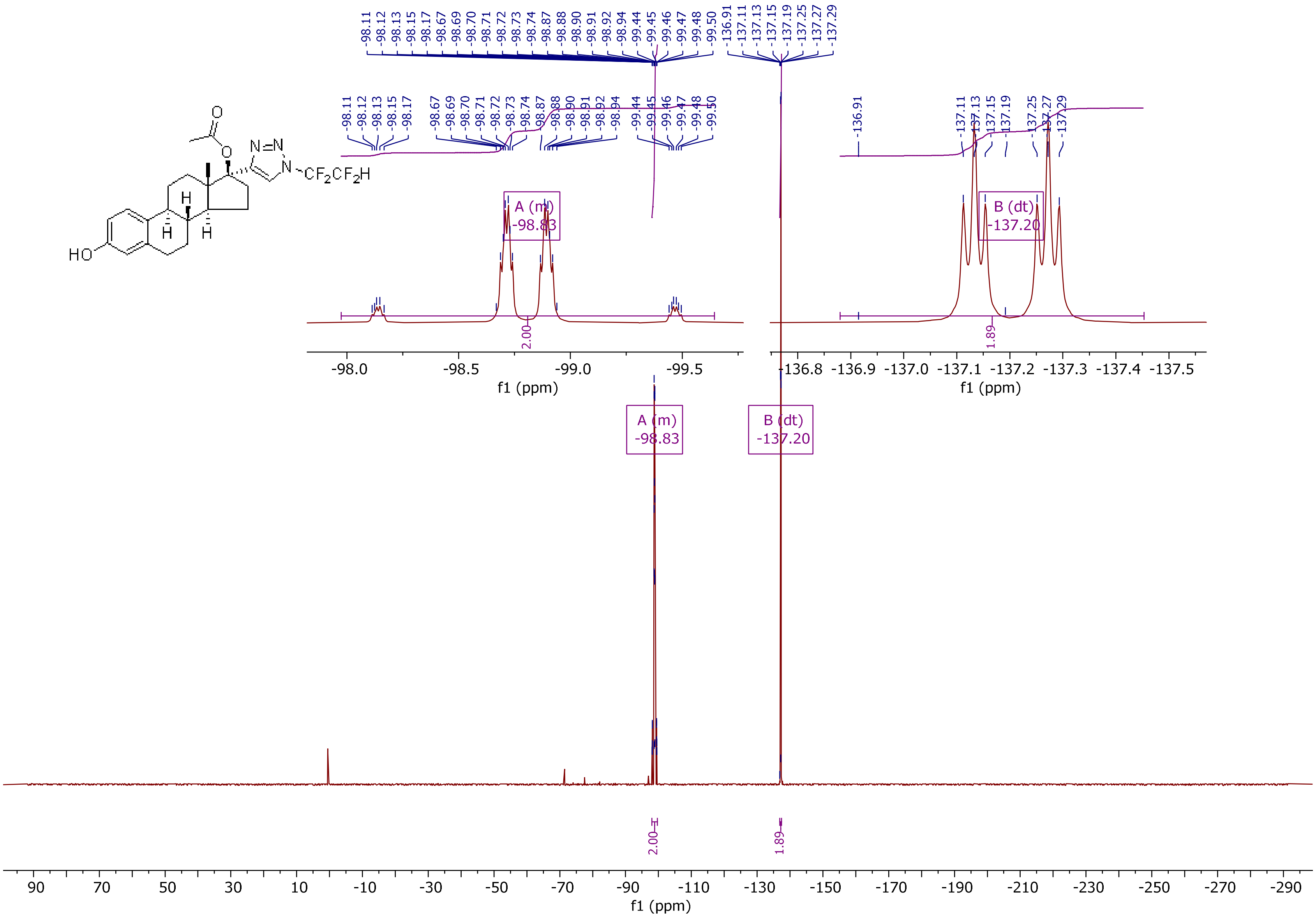

$^{13}C\{^{1}H\}$ NMR spectrum of **2l** ($CDCl_3$, 101 MHz)

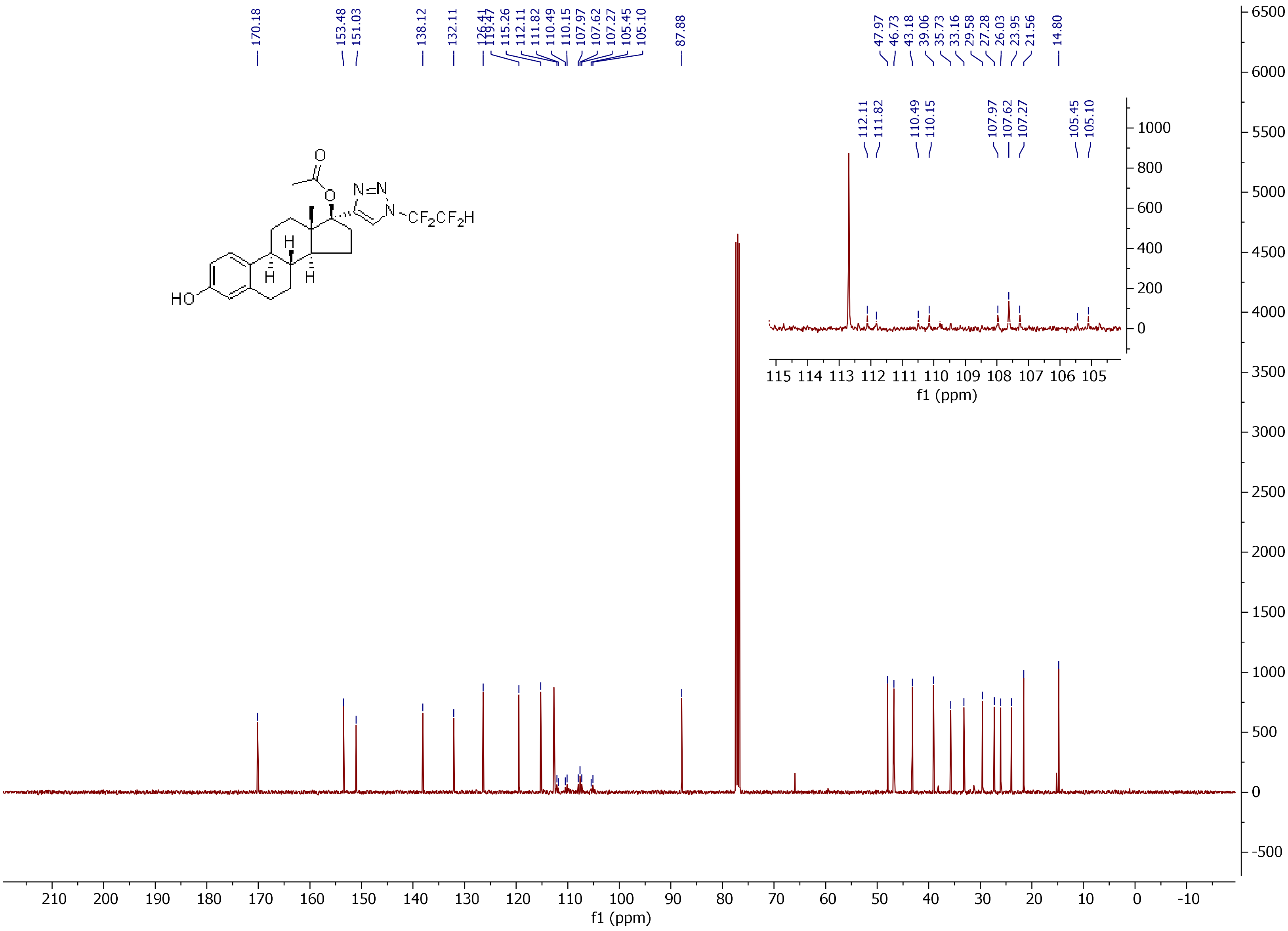

^1H

NMR spectrum of **3a** ($CDCl_3$, 400 MHz)

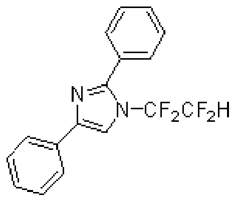

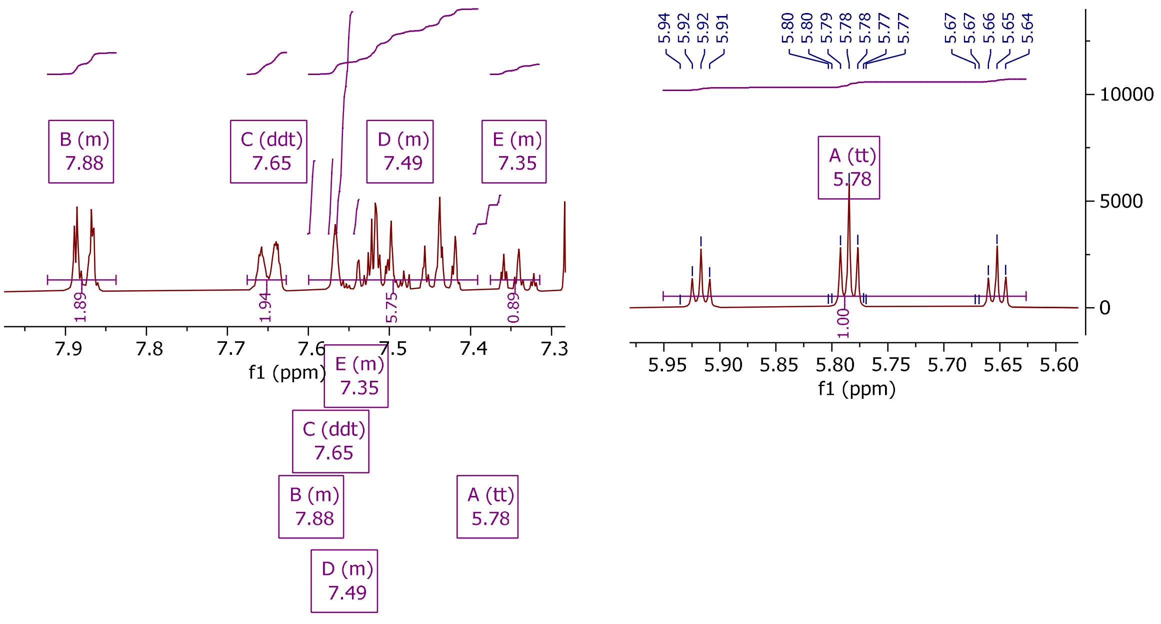

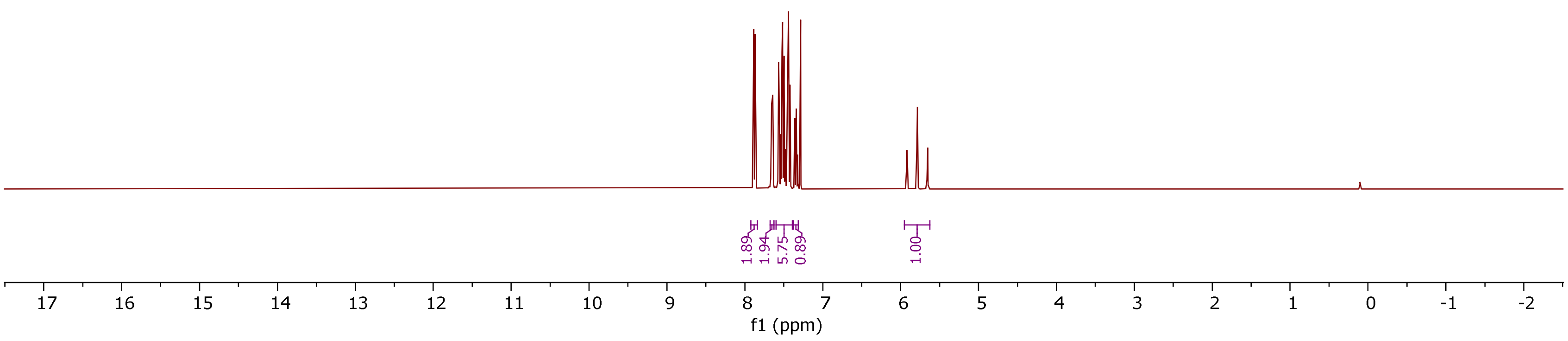

^{19}F NMR spectrum of **3a** ($CDCl_3$, 377 MHz)

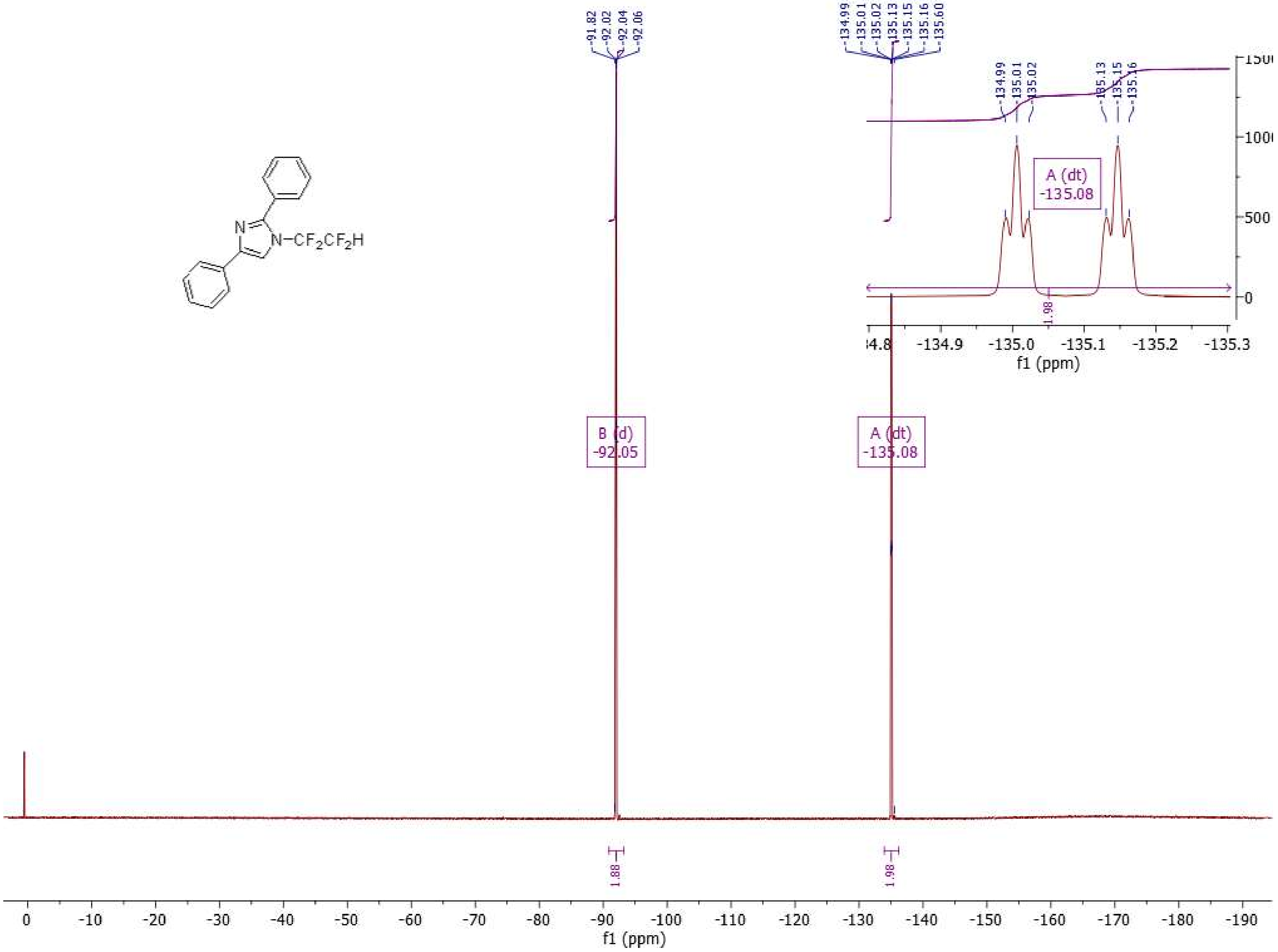

$^{13}C\{^1H\}$ NMR spectrum of **3a** ($CDCl_3$, 101 MHz)

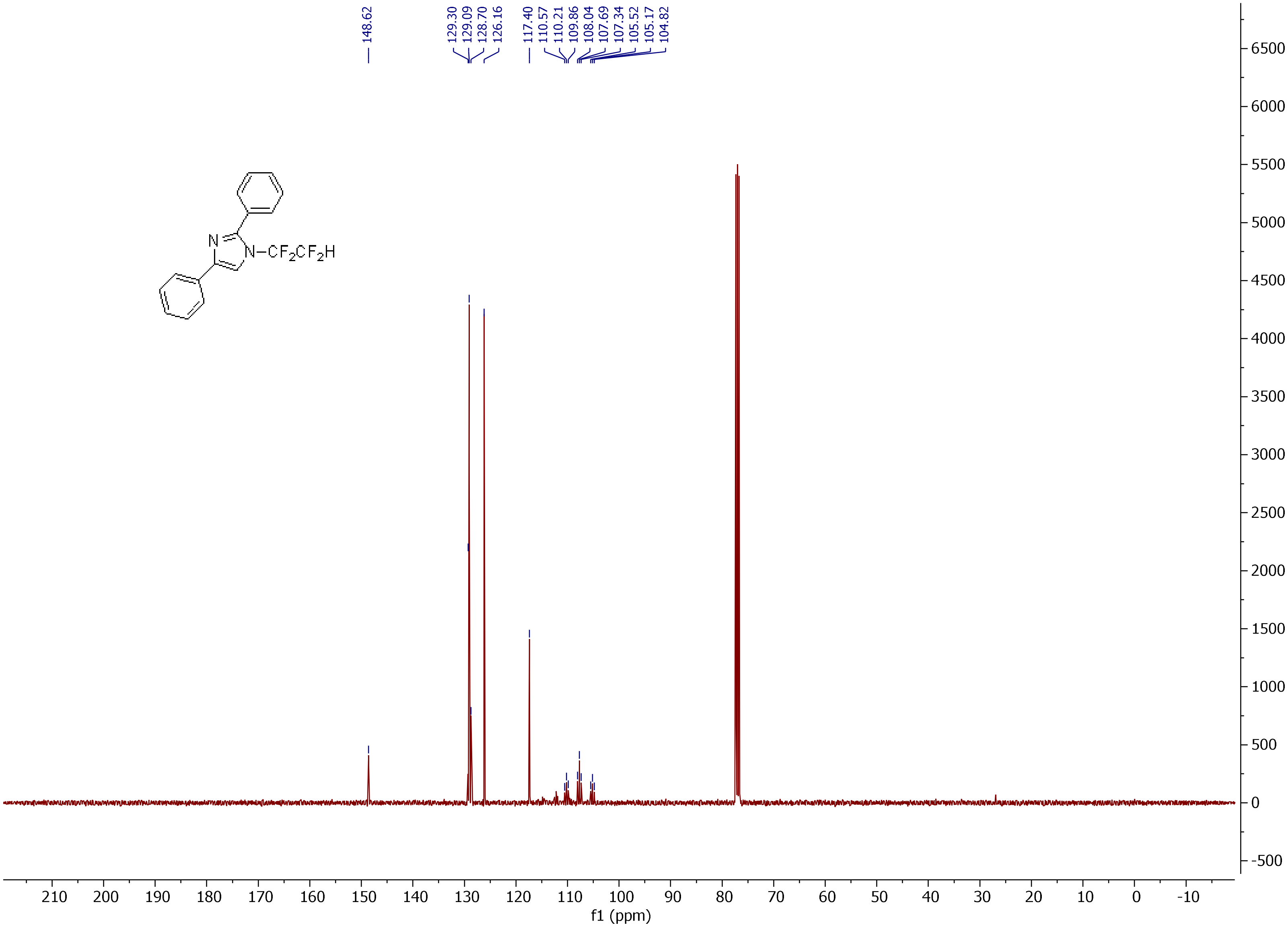

^{1}H NMR spectrum of **3d** ($CDCl_3$, 400 MHz)

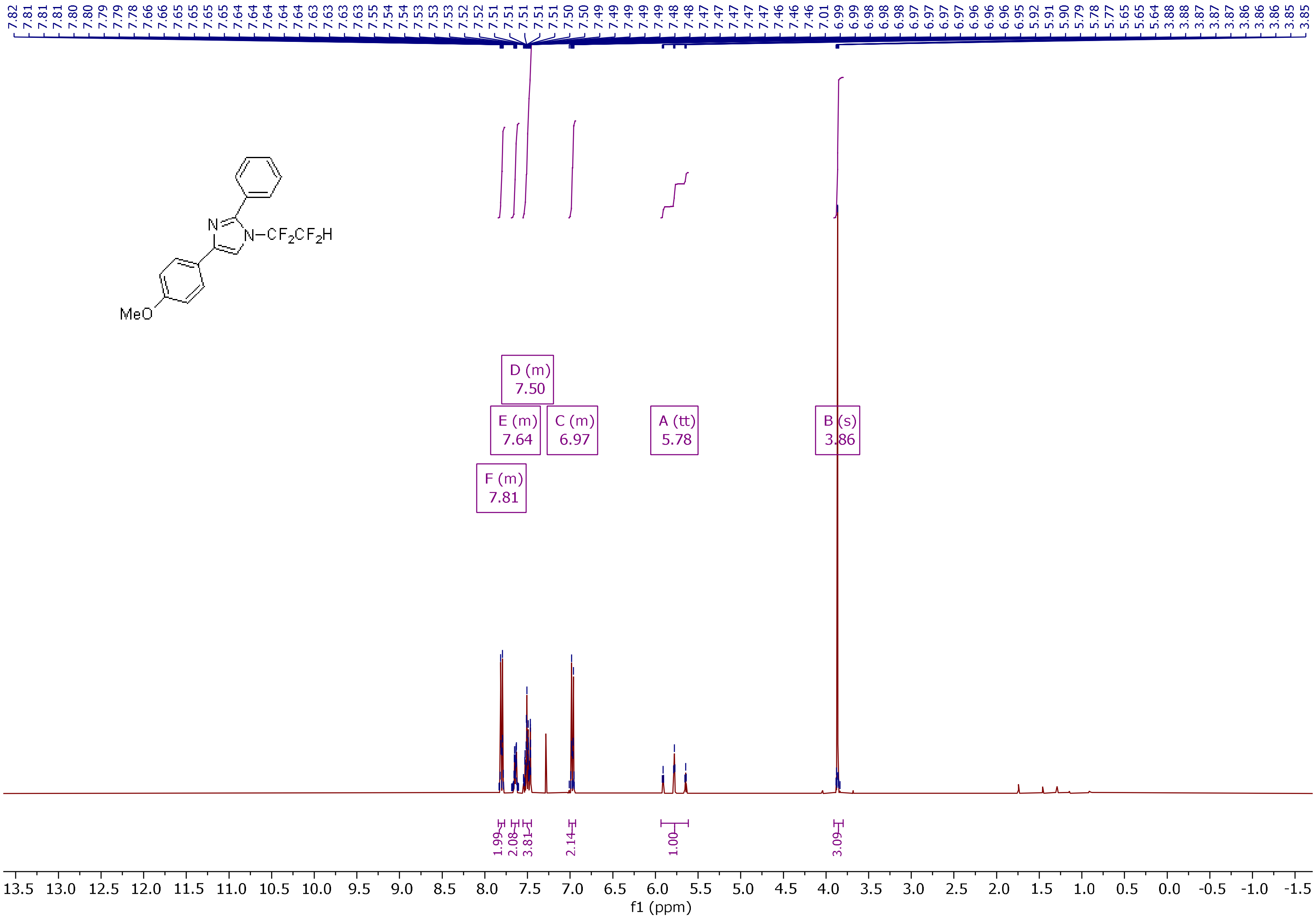

^{19}F NMR spectrum of **3d** ($CDCl_3$, 377 MHz)

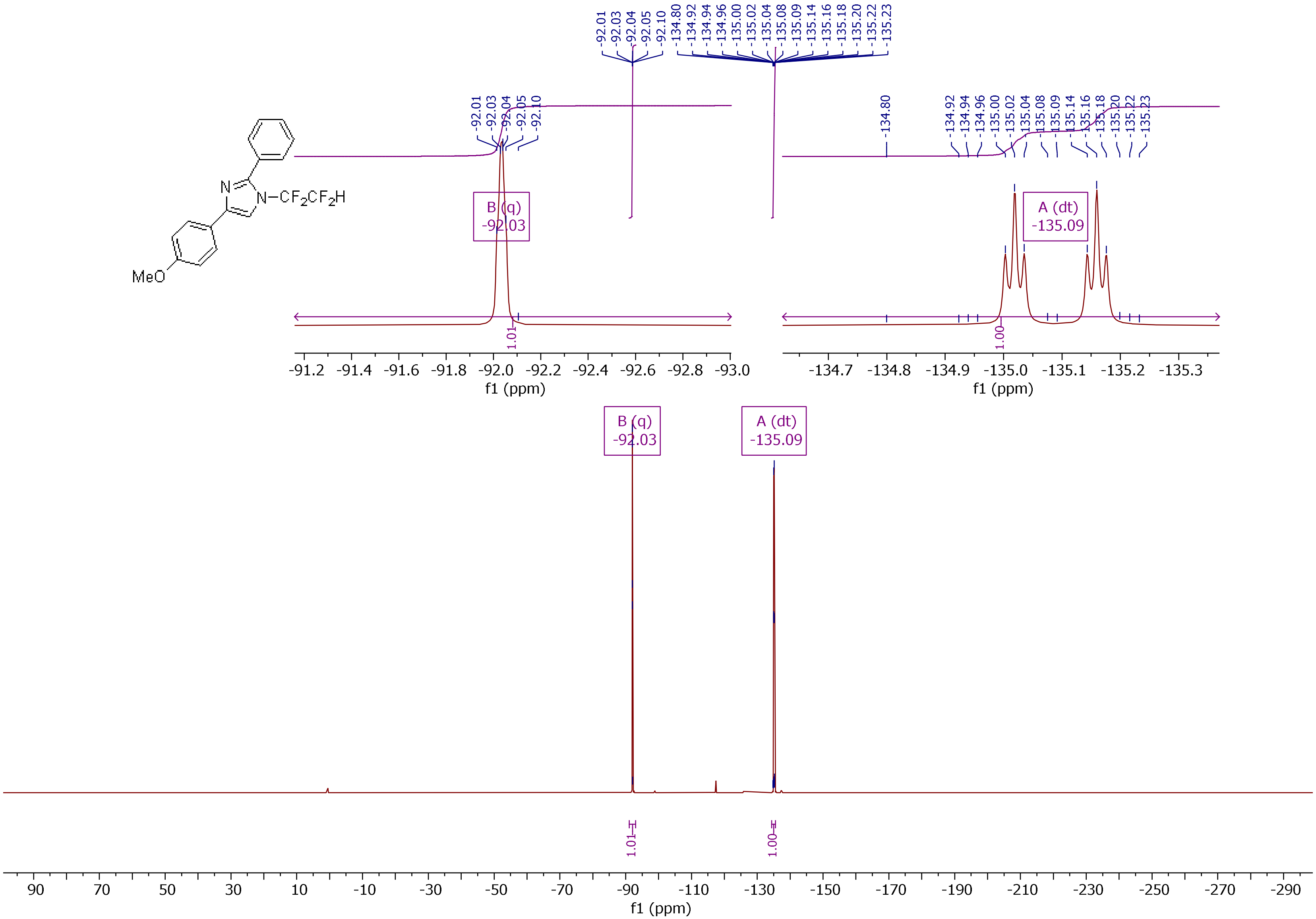

$^{13}C\{^{1}H\}$ NMR spectrum of **3d** ($CDCl_3$, 101 MHz)

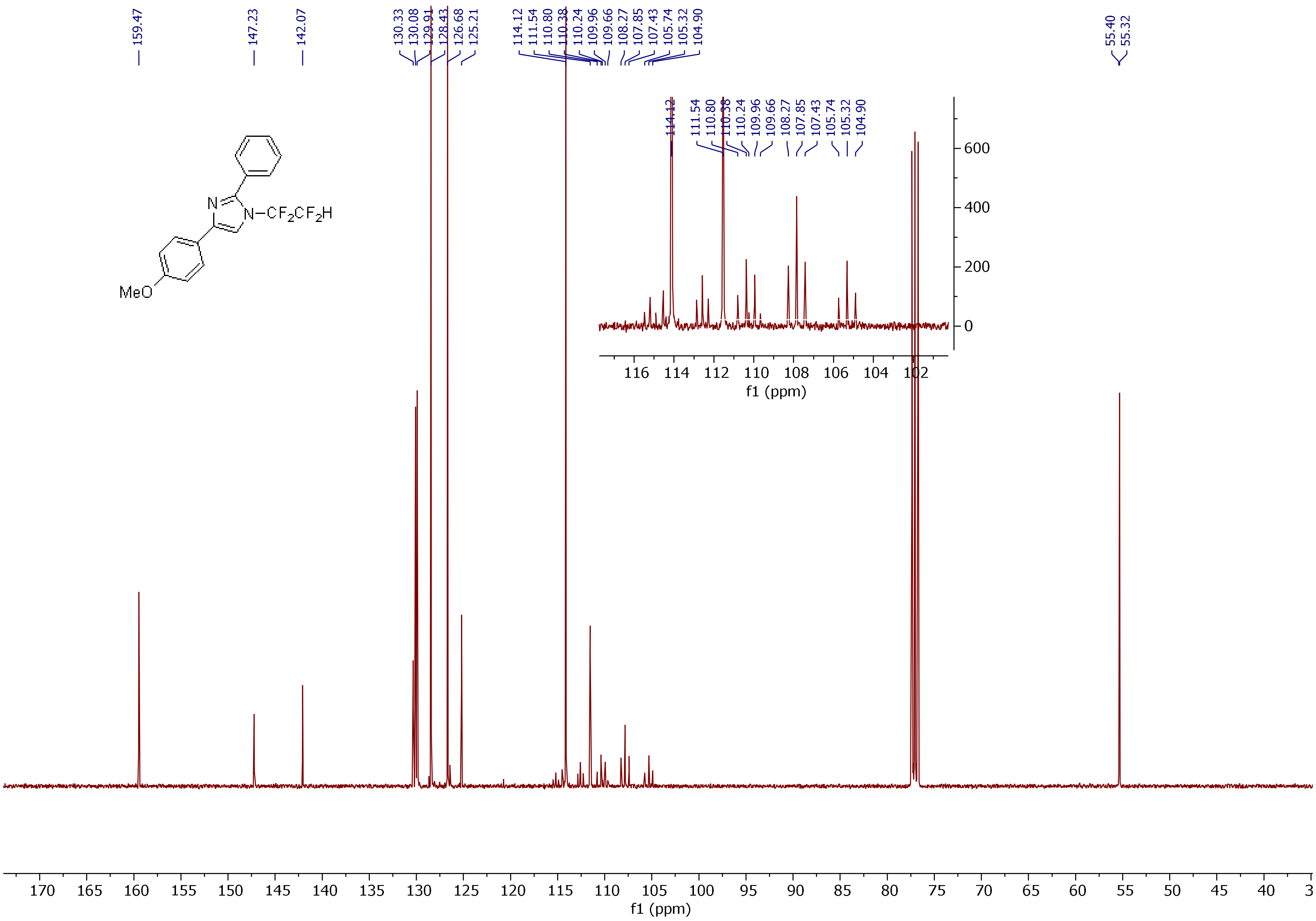

^{1}H NMR spectrum of **3e** ($CDCl_3$, 400 MHz)

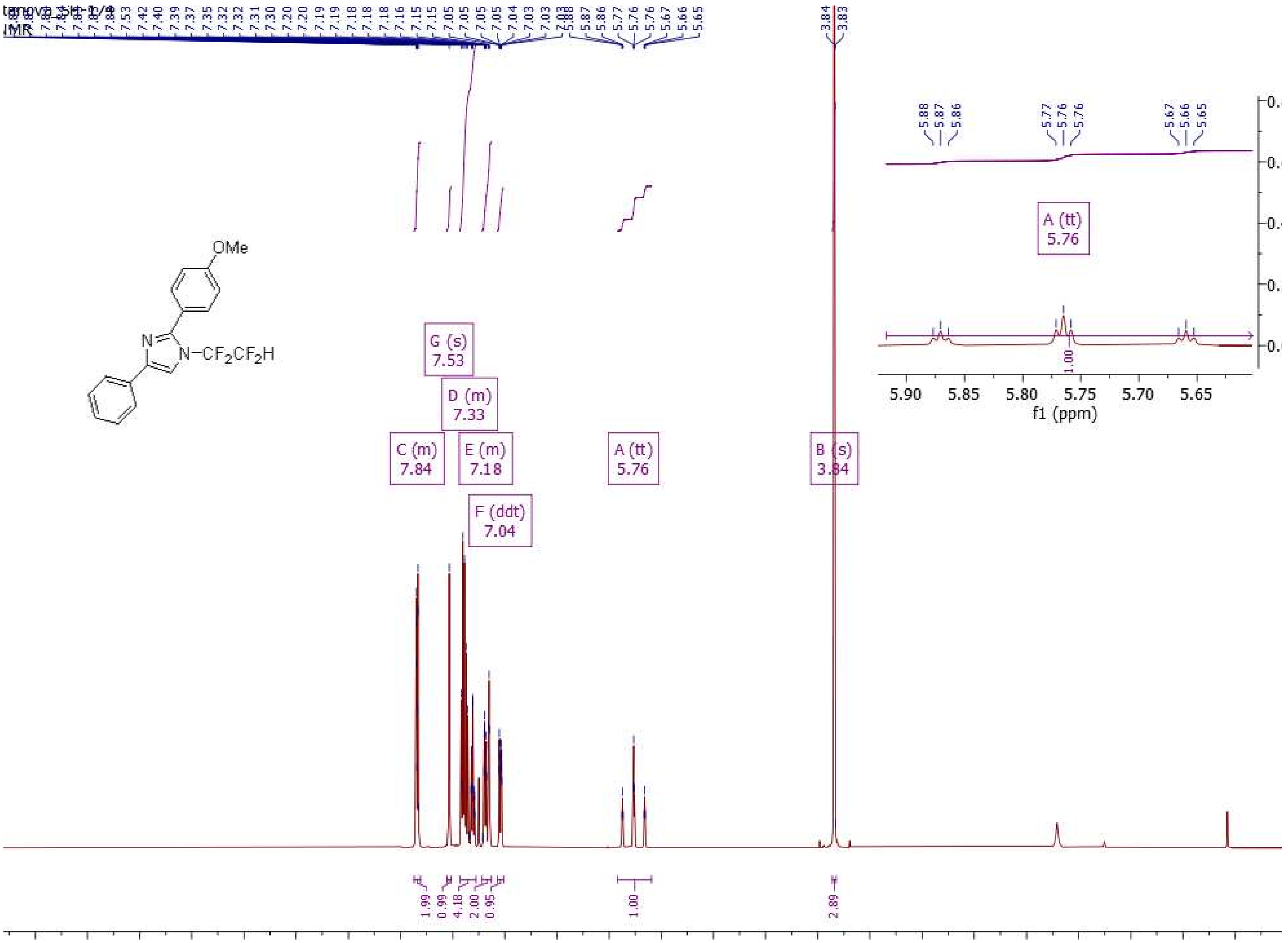

^{19}F NMR spectrum of **3e** ($CDCl_3$, 377 MHz)

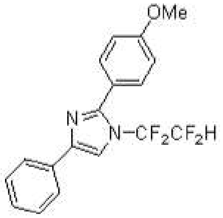

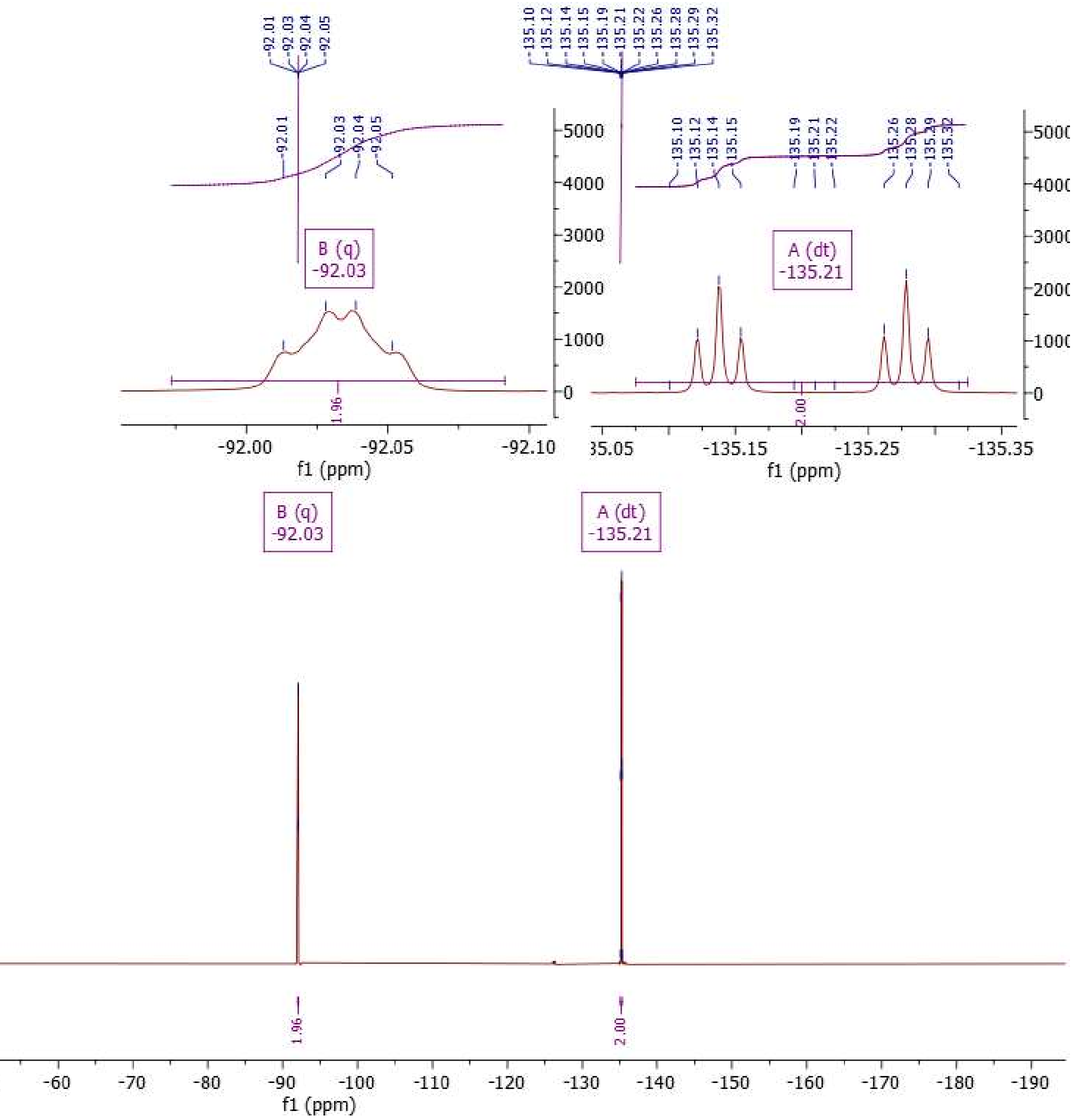

$^{13}C\{^1H\}$ NMR spectrum of **3e** ($CDCl_3$, 101 MHz)

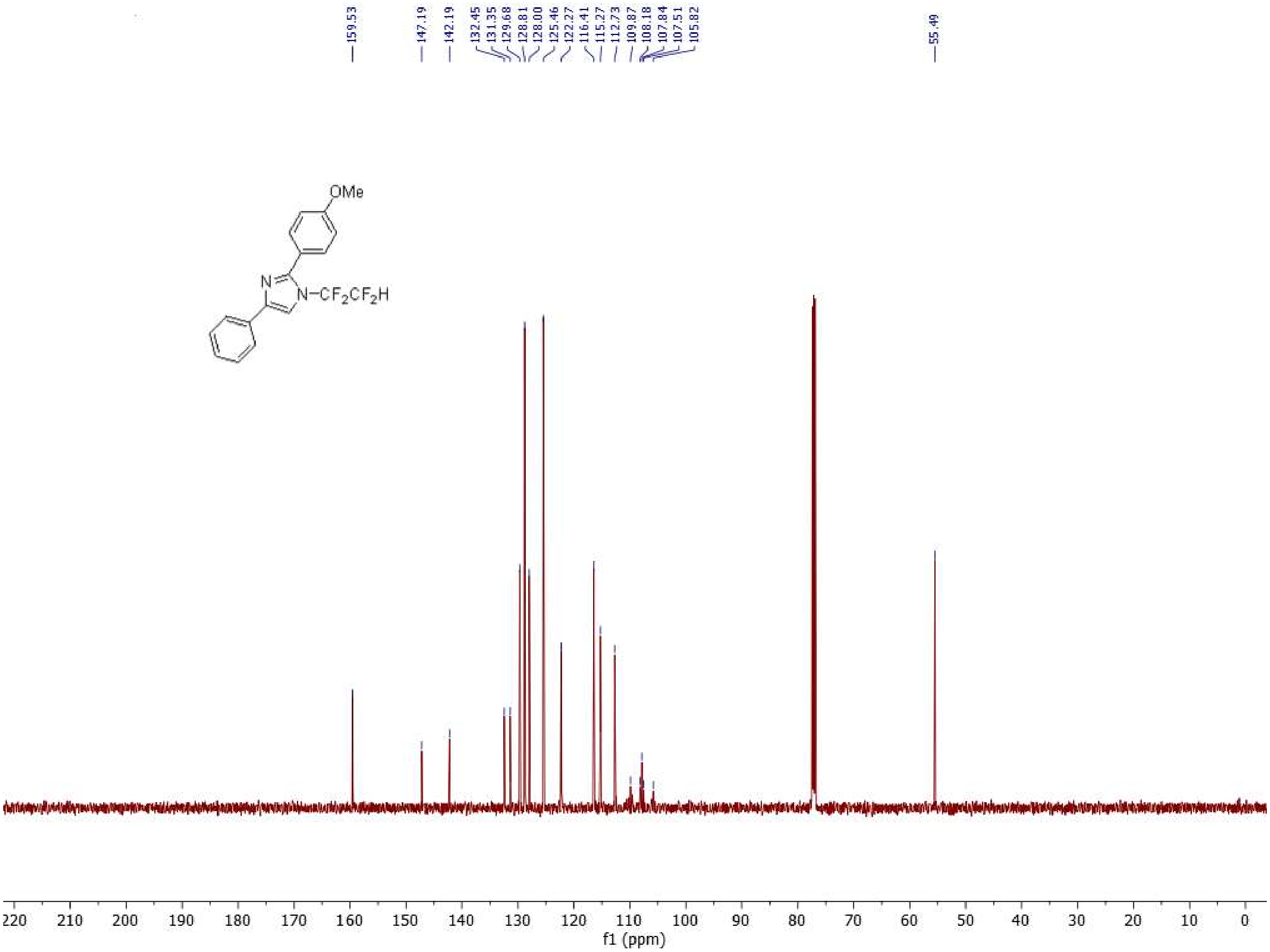

^{1}H NMR spectrum of **3f** ($CDCl_3$, 400 MHz)

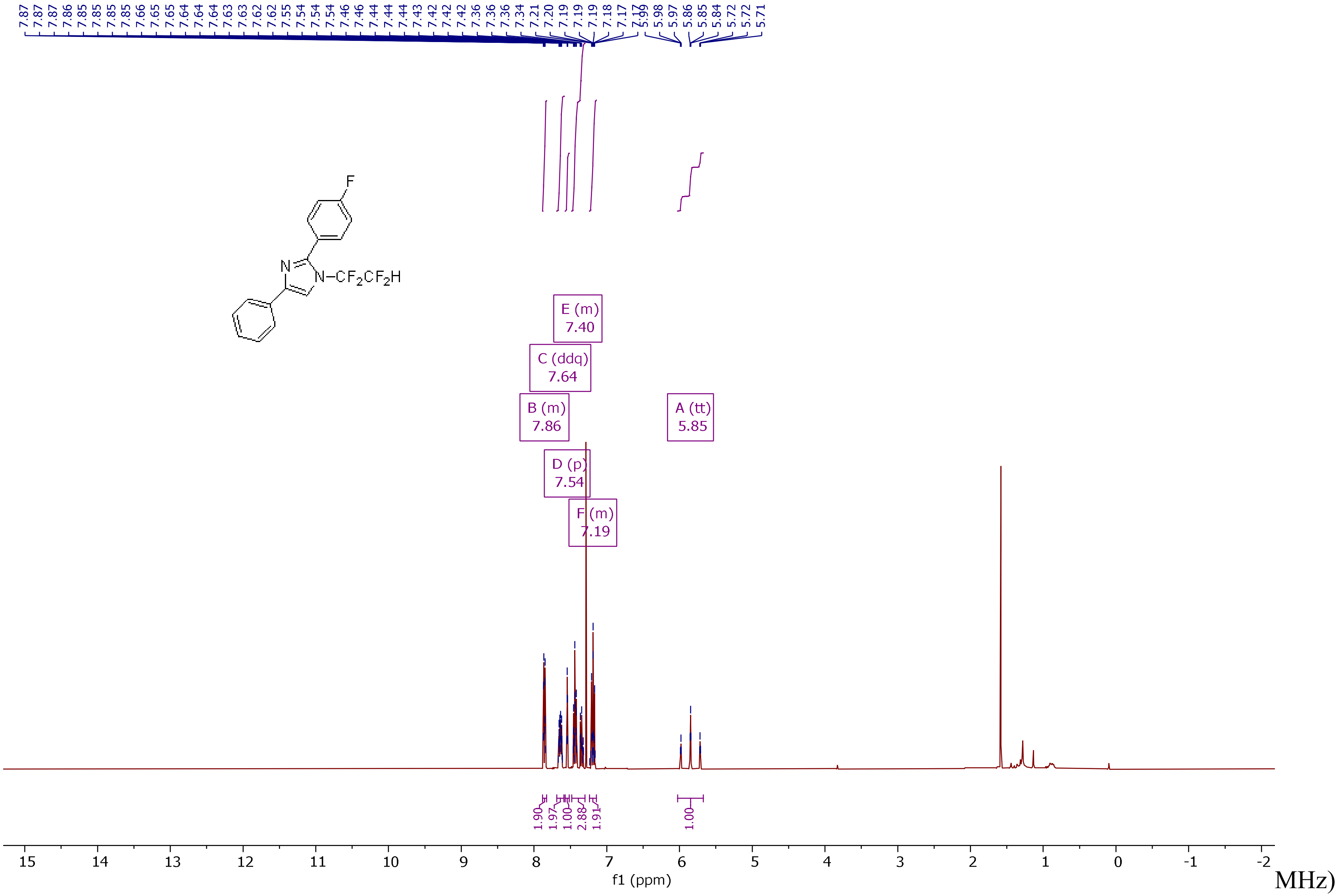

^{19}F NMR spectrum of **3f** ($CDCl_3$, 377 MHz)

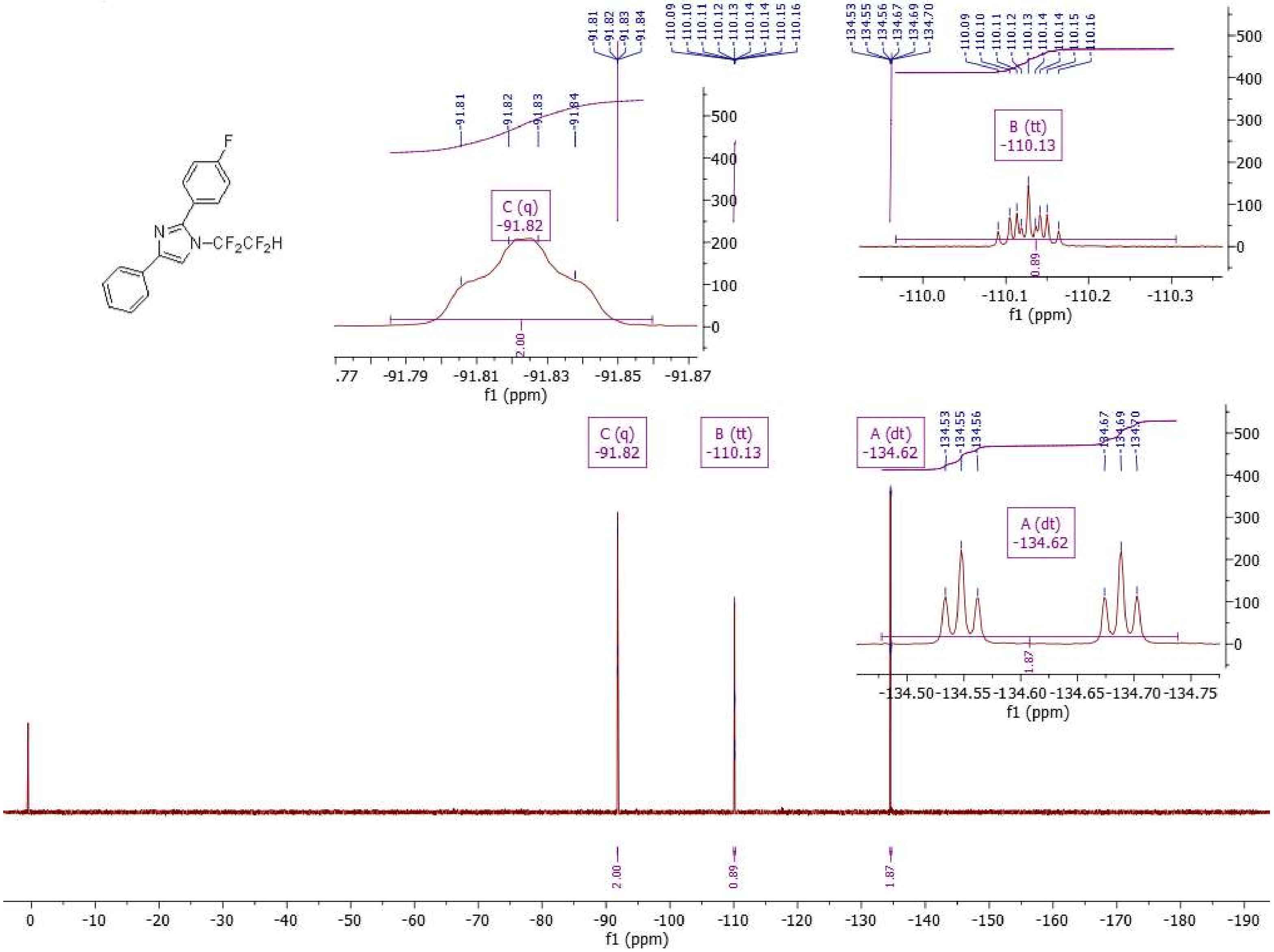

$^{13}C\{^1H\}$ NMR spectrum of **3f** ($CDCl_3$, 101 MHz)

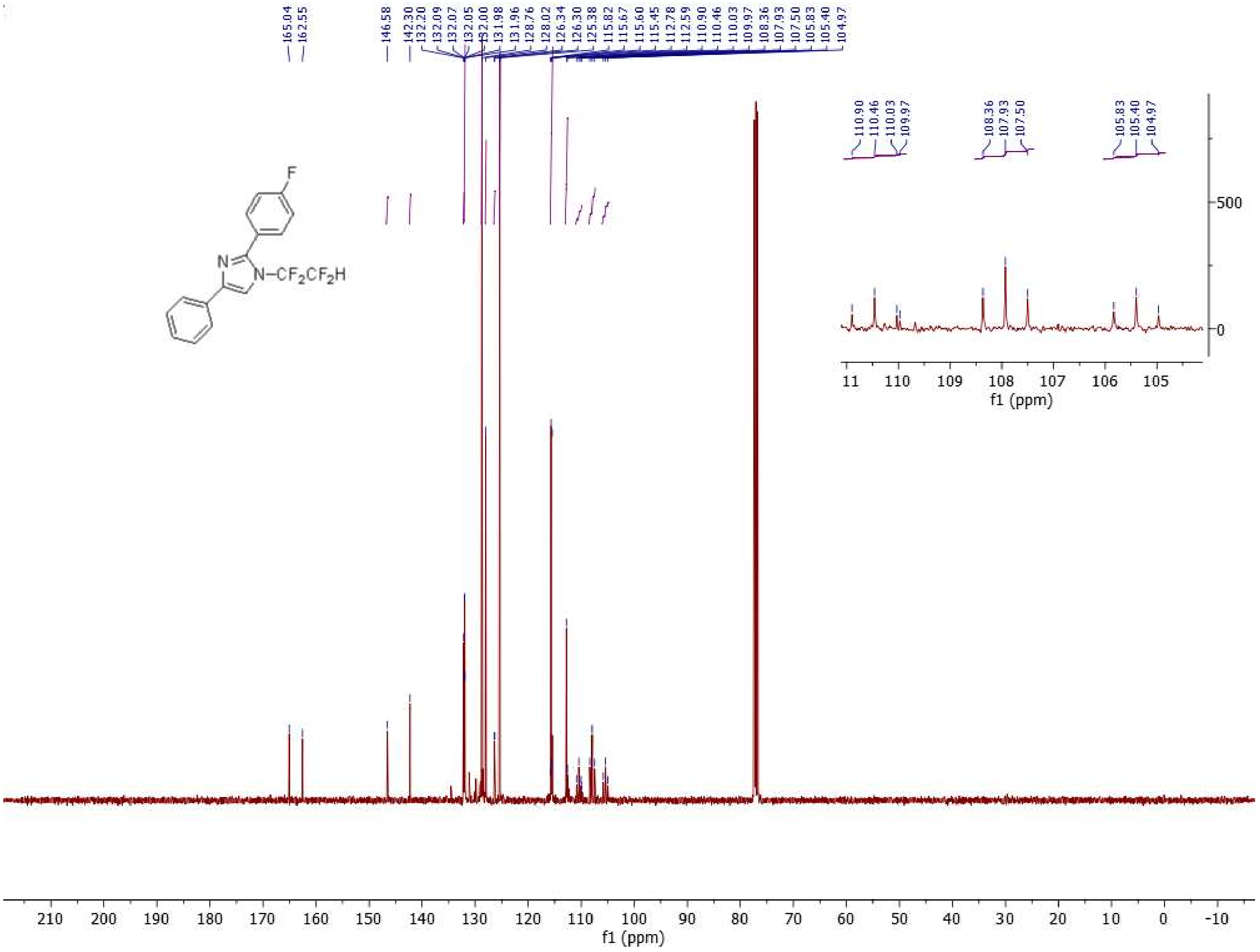

^{1}H NMR spectrum of **3g** ($CDCl_3$, 400 MHz)

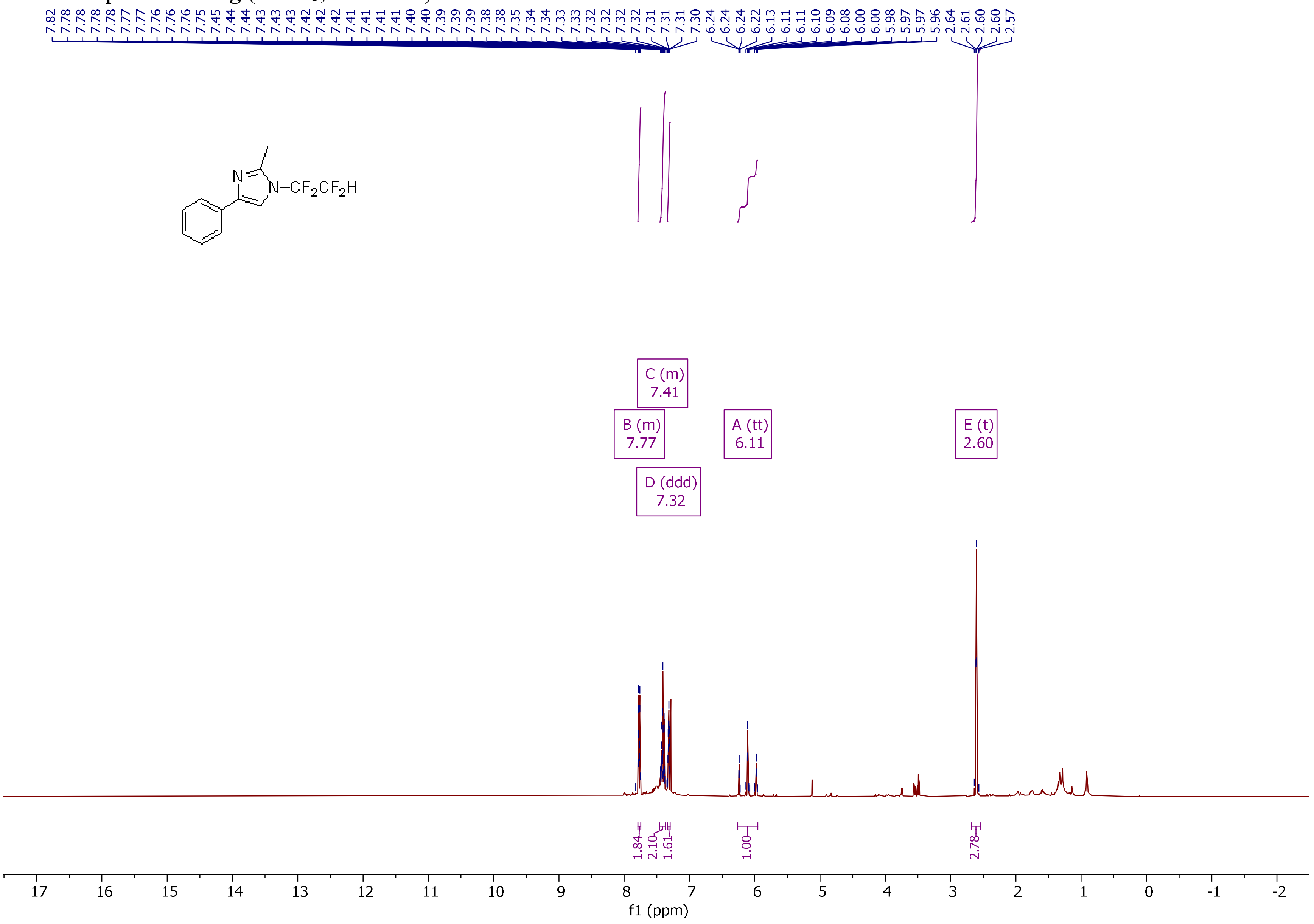

^{19}F NMR spectrum of **3g** ($CDCl_3$, 377 MHz)

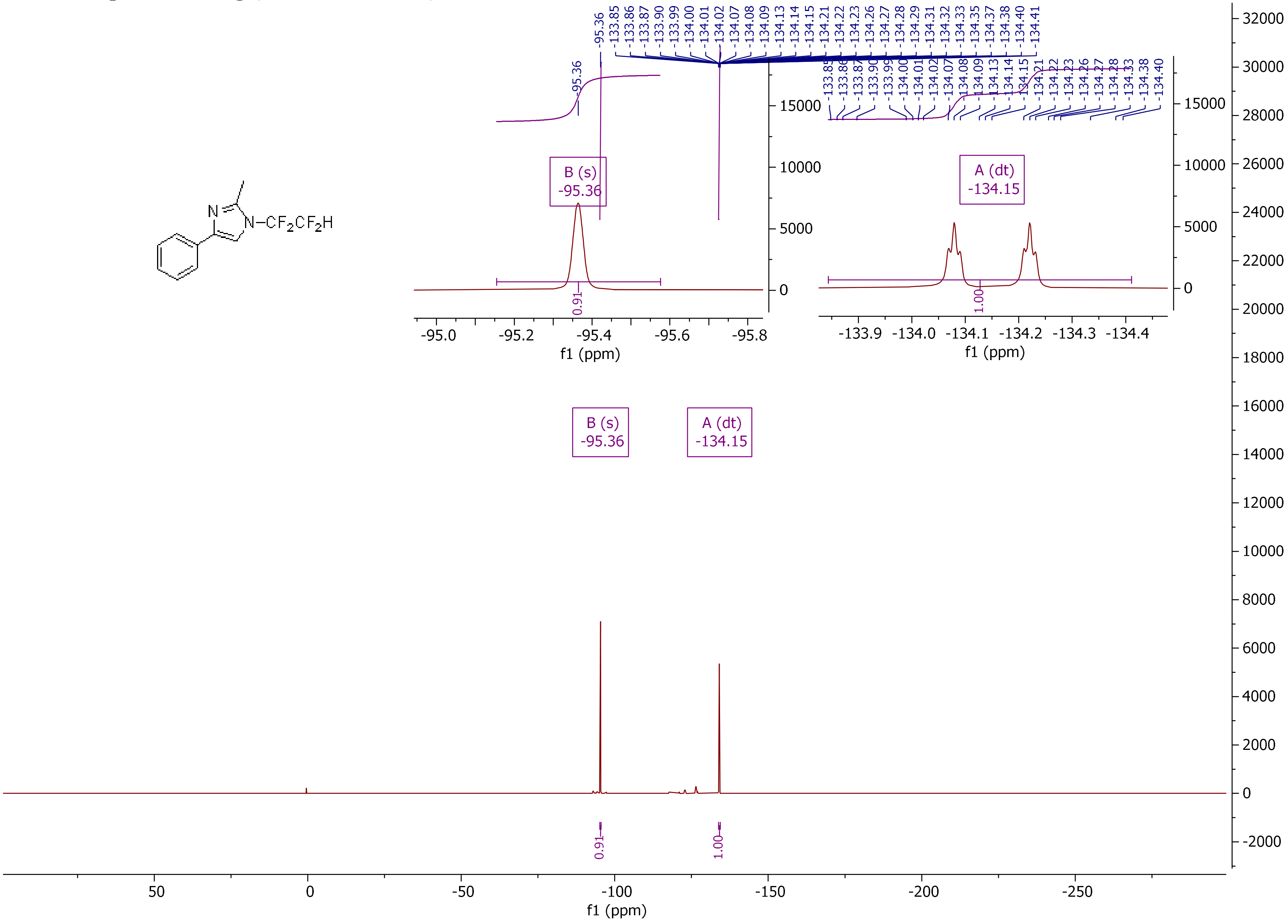

$^{13}C\{^1H\}$ NMR spectrum of **3g** ($CDCl_3$, 101 MHz)

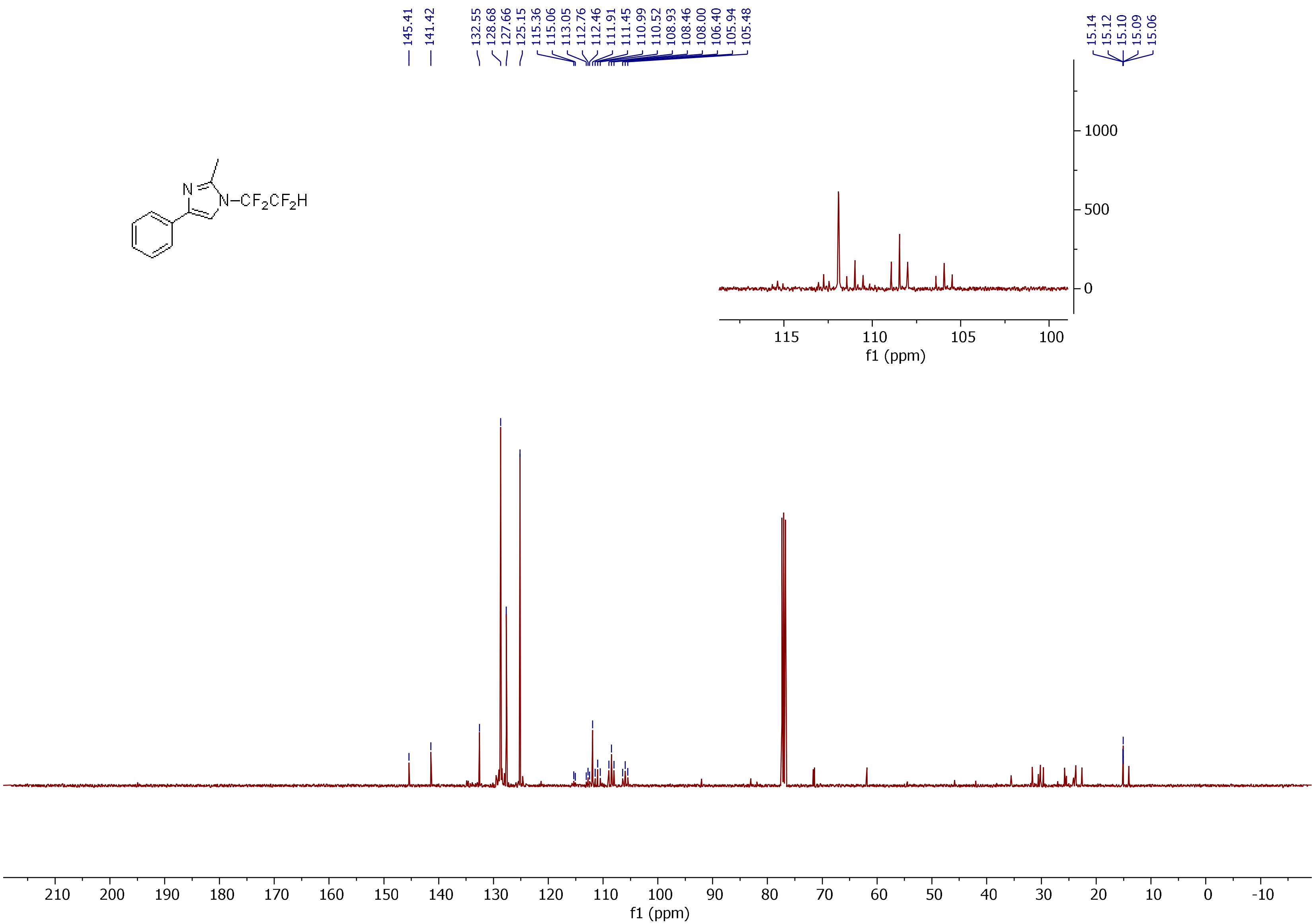

^{1}H NMR spectrum of **4** ($CDCl_3$, 400 MHz)

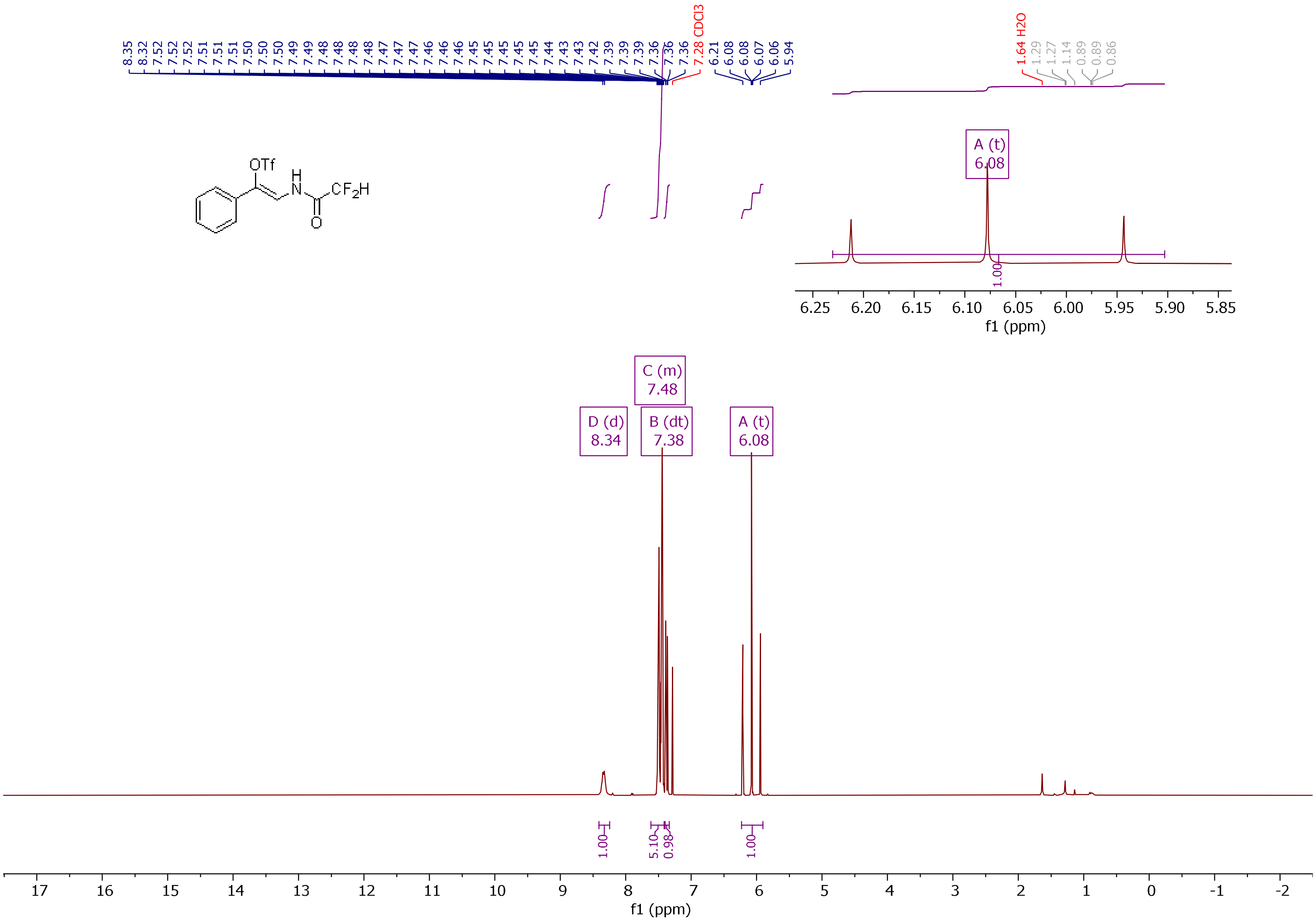

^{19}F NMR spectrum of **4** (CDCl$_3$, 377 MHz)

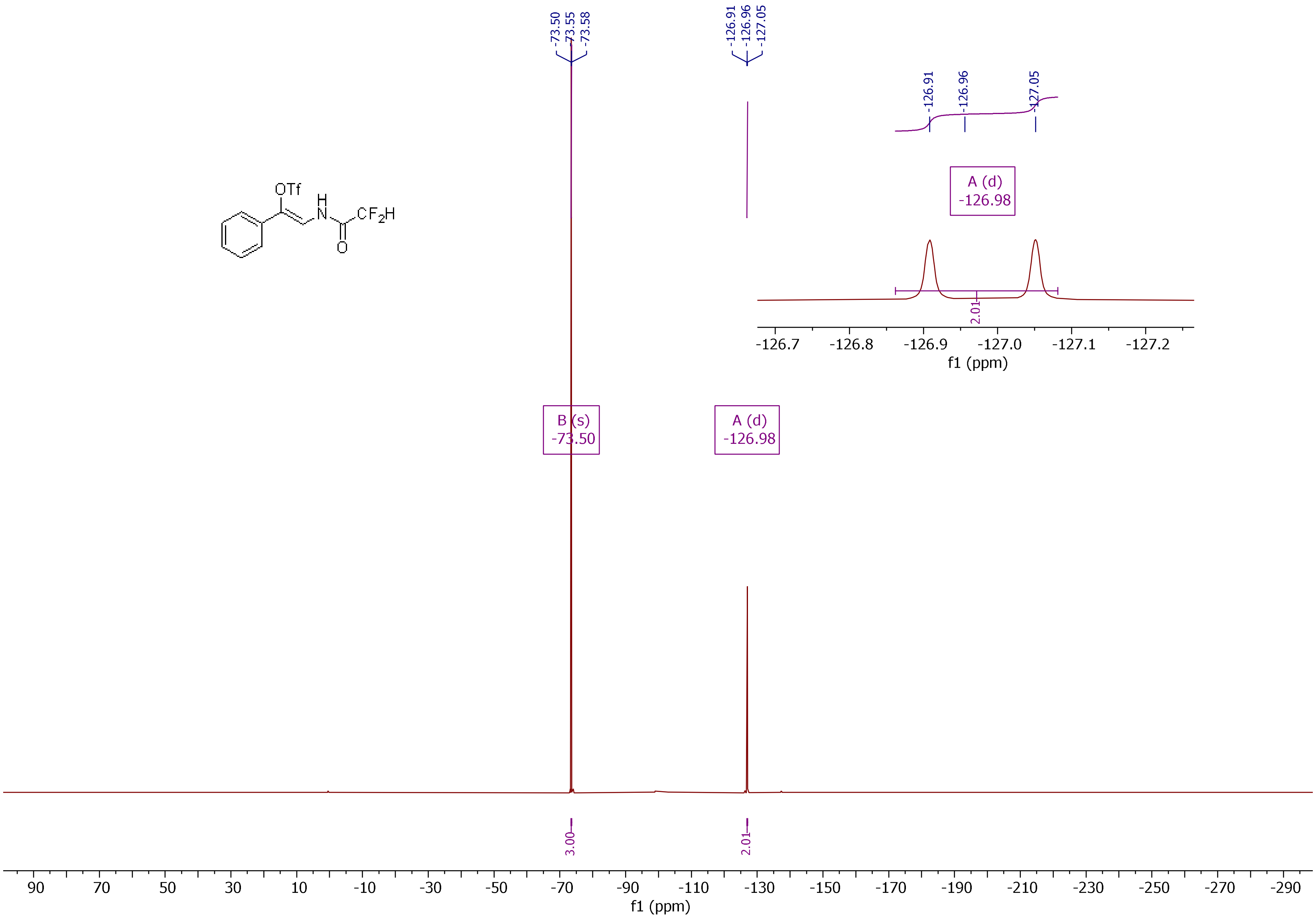

$^{13}C\{^1H\}$ NMR spectrum of **4** ($CDCl_3$, 101 MHz)

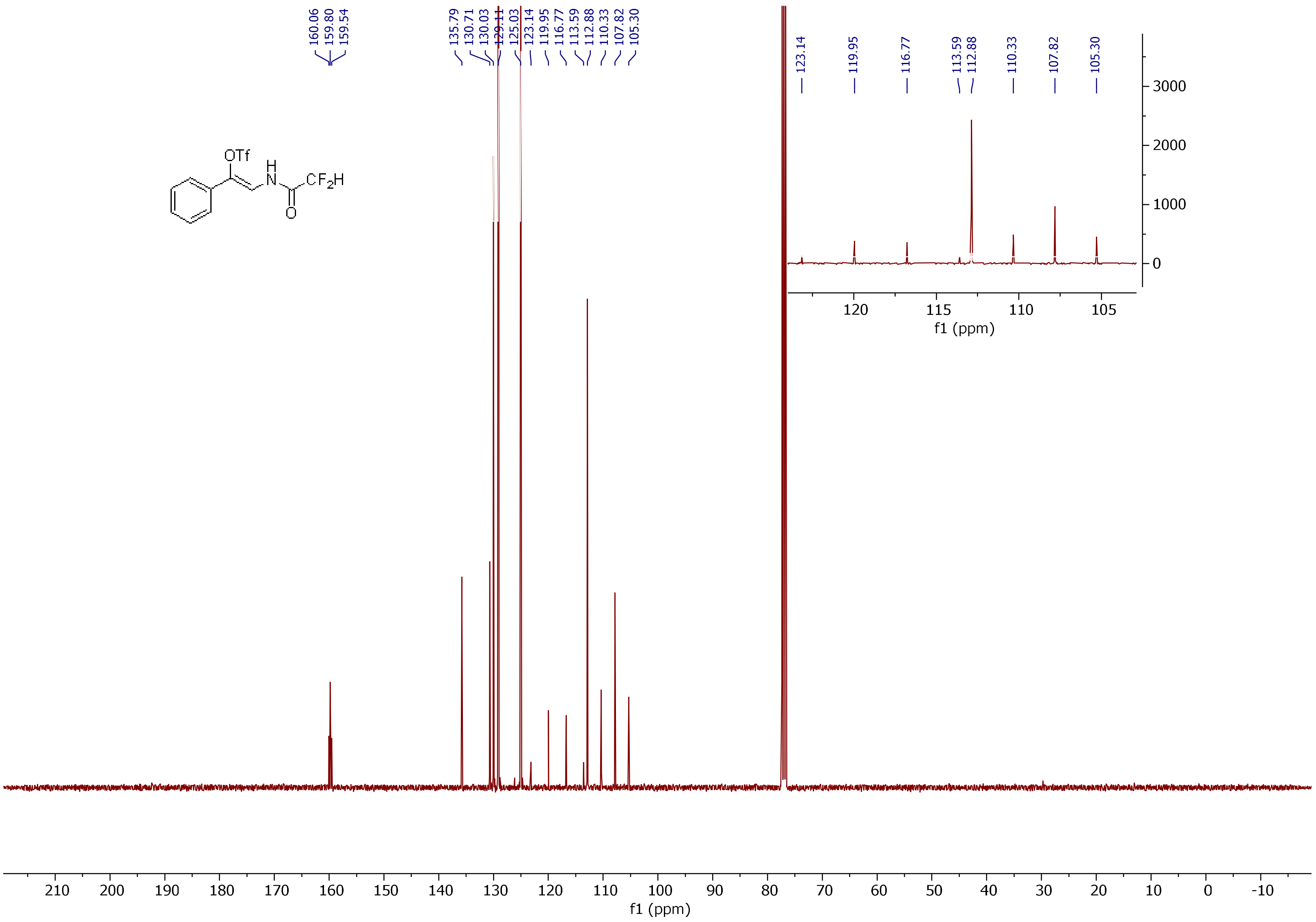

^{1}H NMR spectrum of **5a** ($CDCl_3$, 400 MHz)

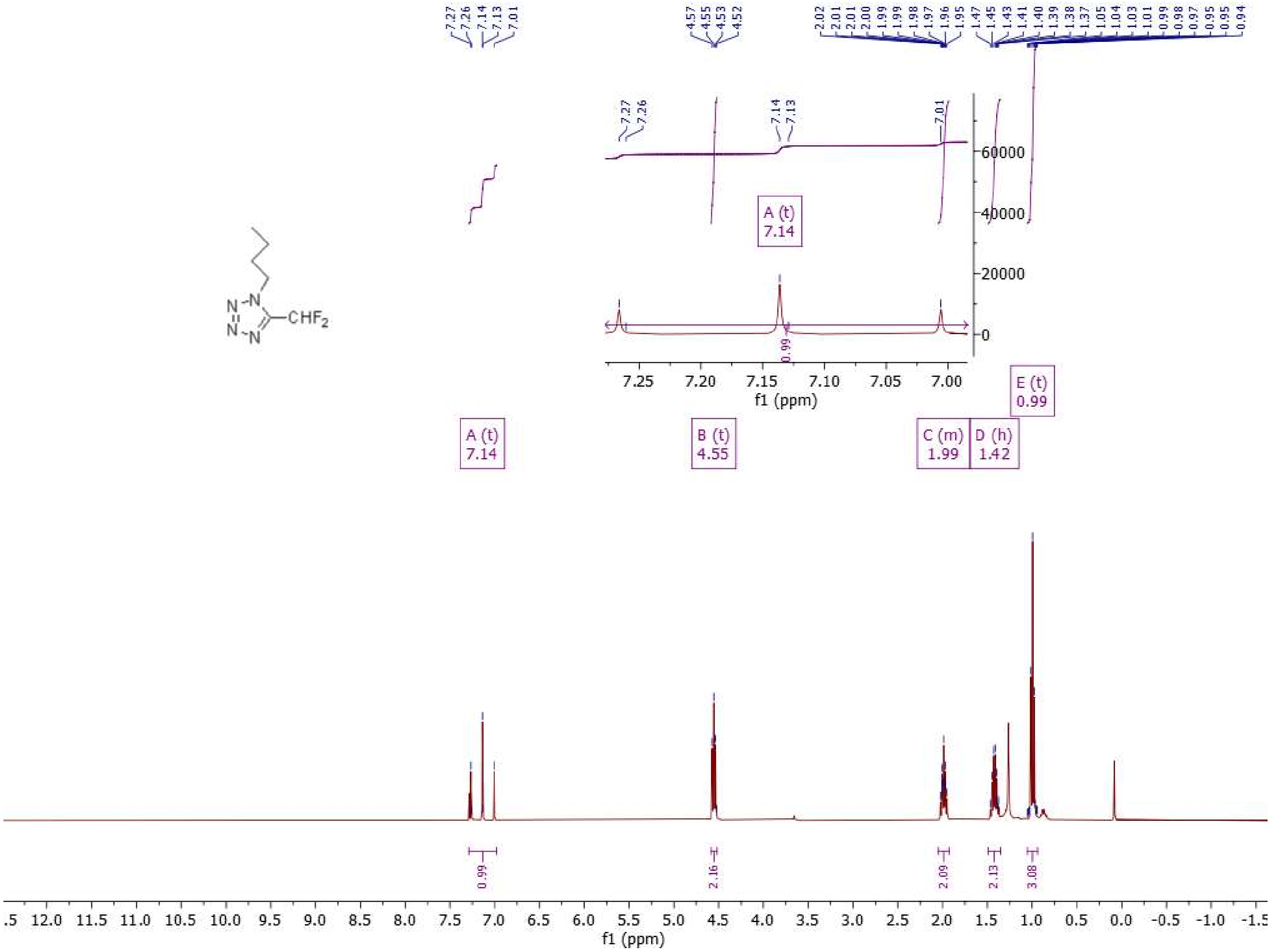

^{19}F NMR spectrum of **5a** ($CDCl_3$, 377 MHz)

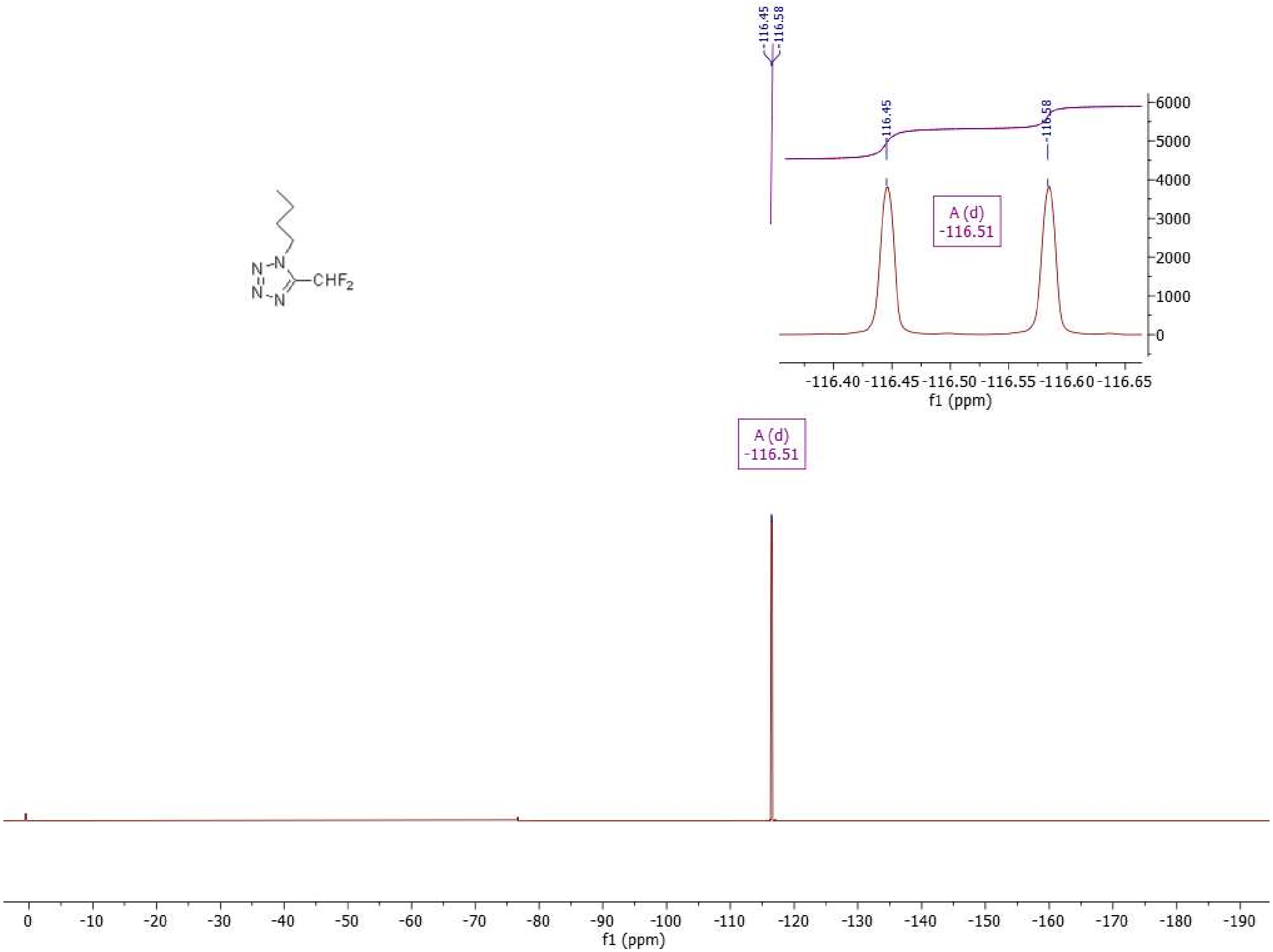

$^{13}C\{^1H\}$ NMR spectrum of **5a** ($CDCl_3$, 101 MHz)

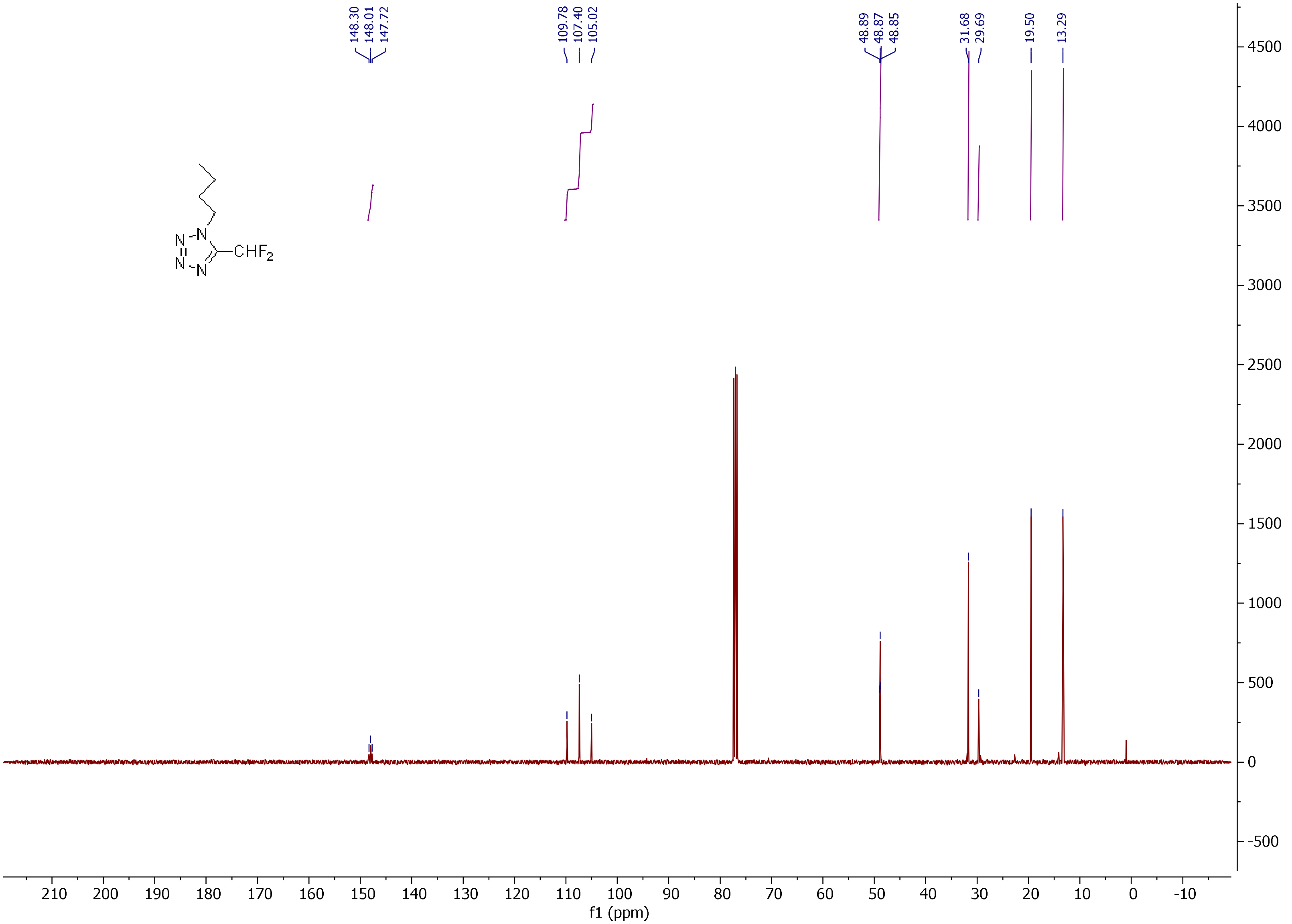

^{1}H NMR spectrum of **5b** ($CDCl_3$, 400 MHz)

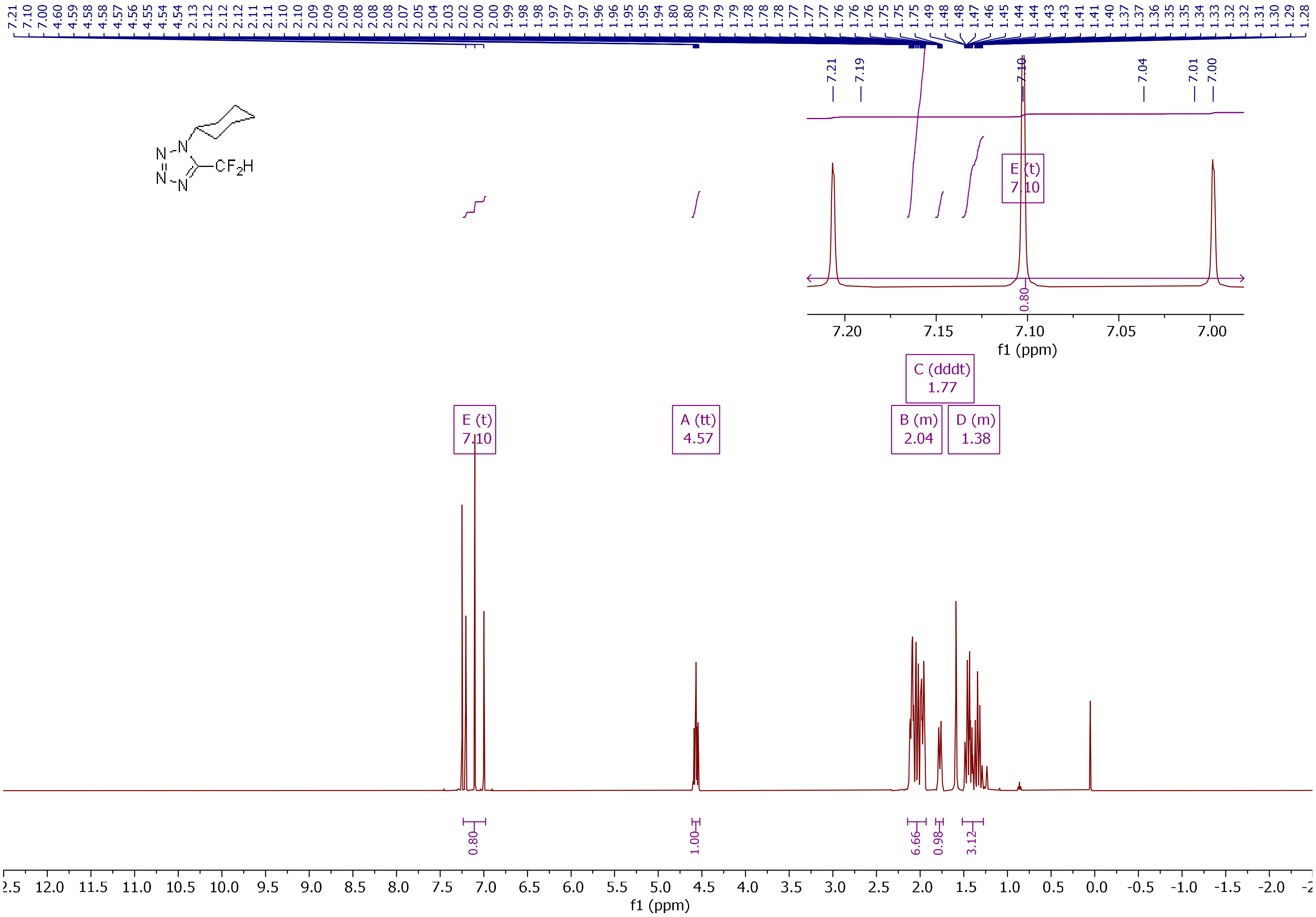

^{19}F NMR spectrum of **5b** ($CDCl_3$, 377 MHz)

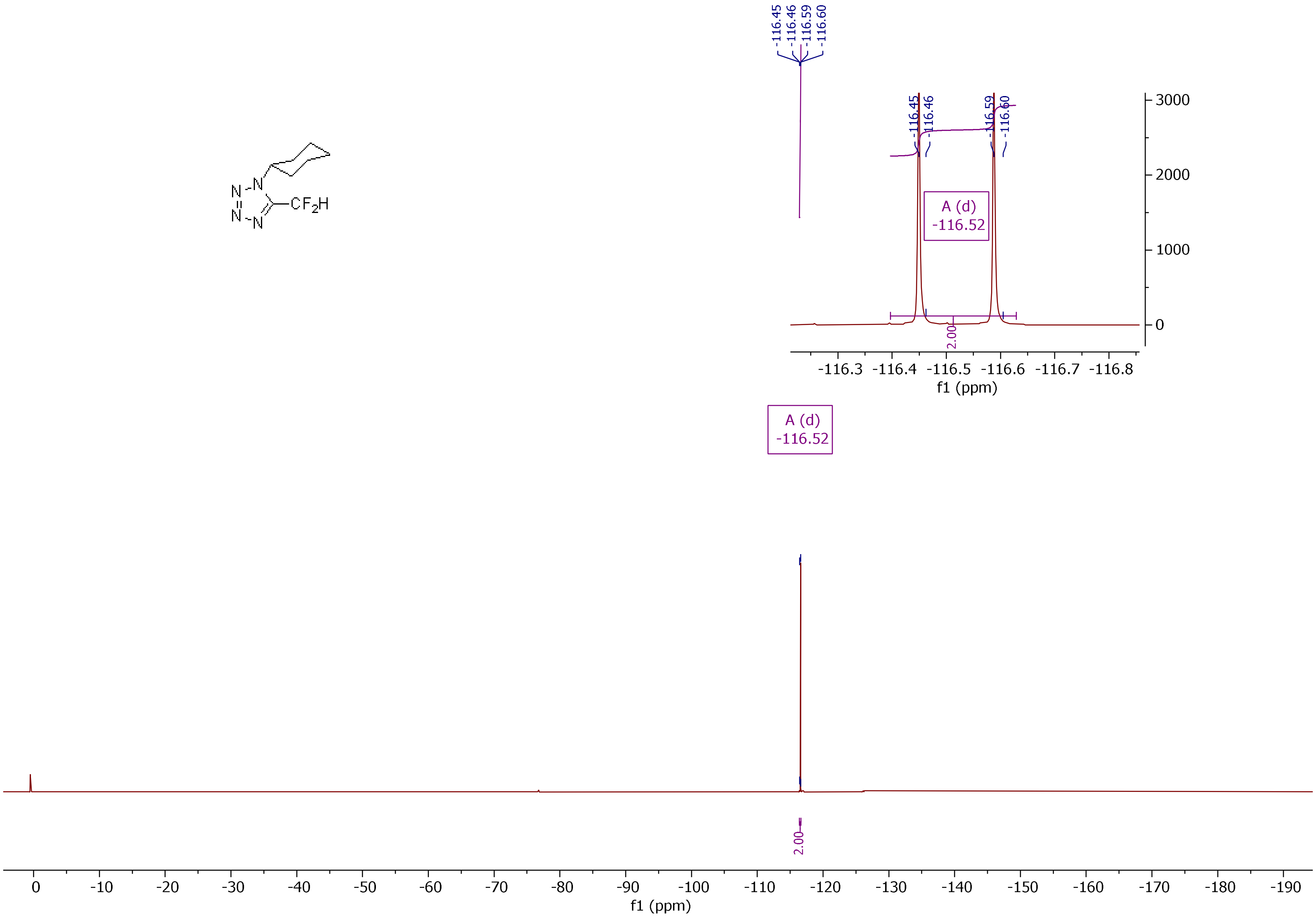

$^{13}C\{^{1}H\}$ NMR spectrum of **5b** ($CDCl_3$, 101 MHz)

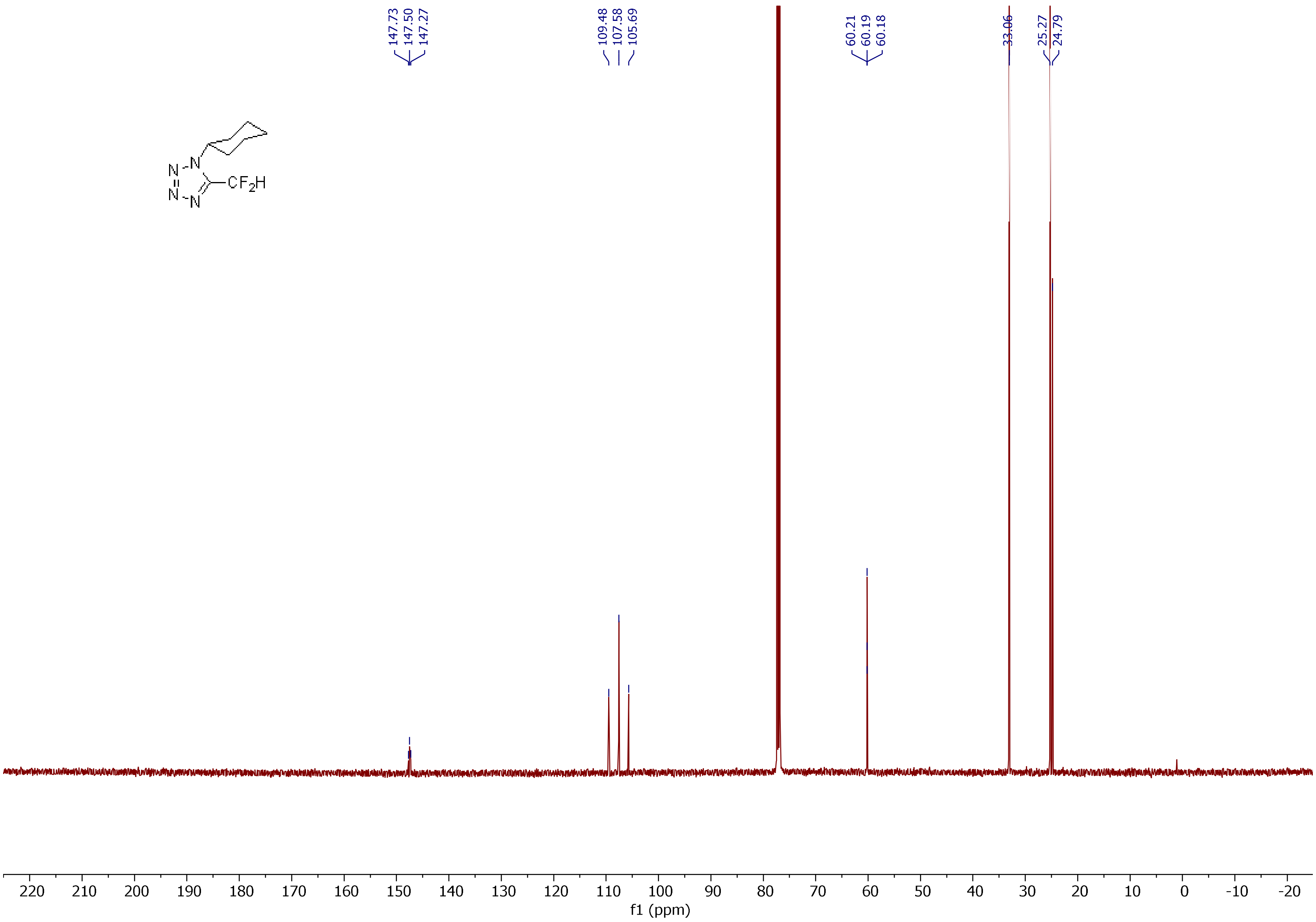

^{1}H NMR spectrum of **5c** ($CDCl_3$, 400 MHz)

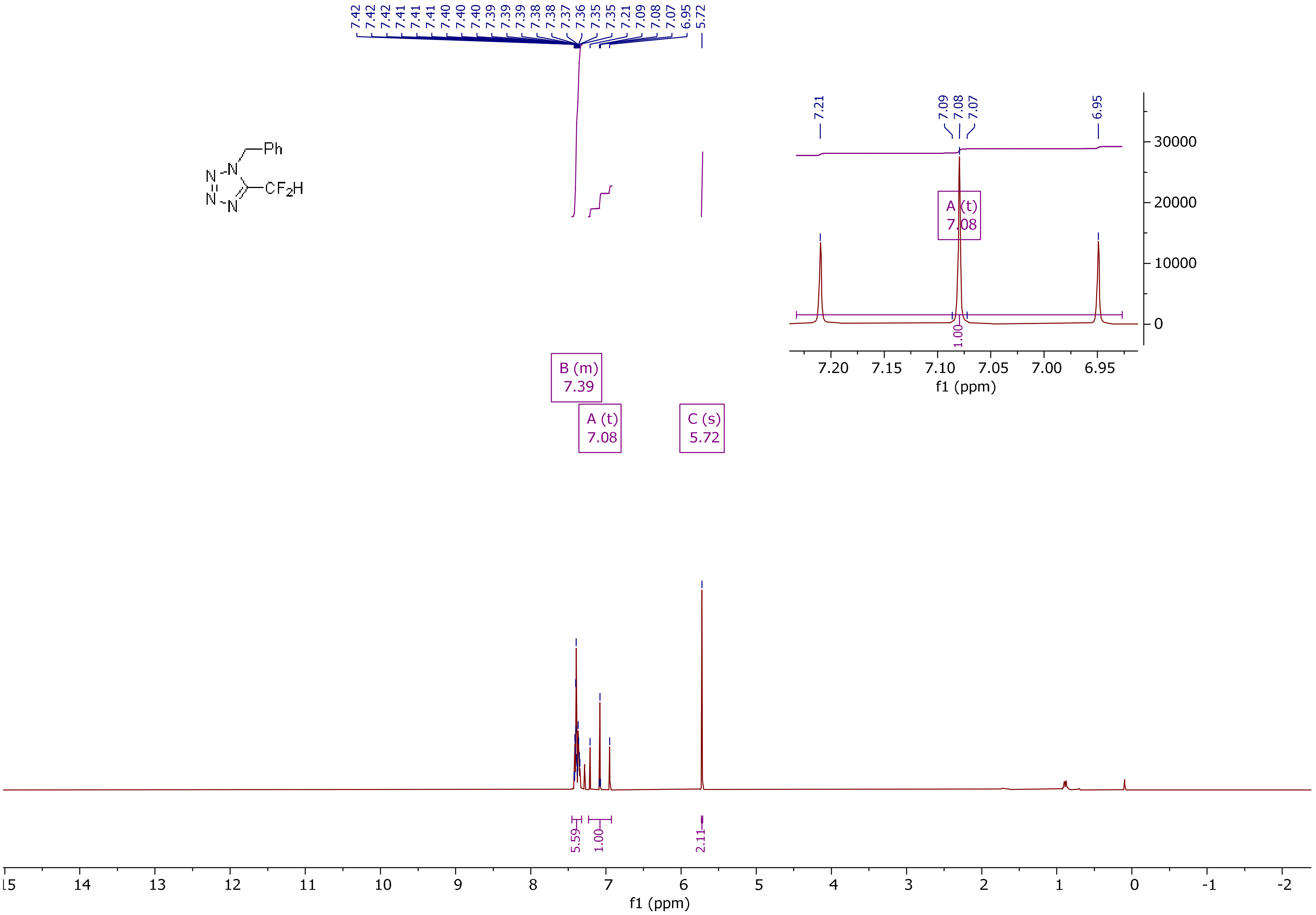

^{19}F NMR spectrum of **5c** ($CDCl_3$, 377 MHz)

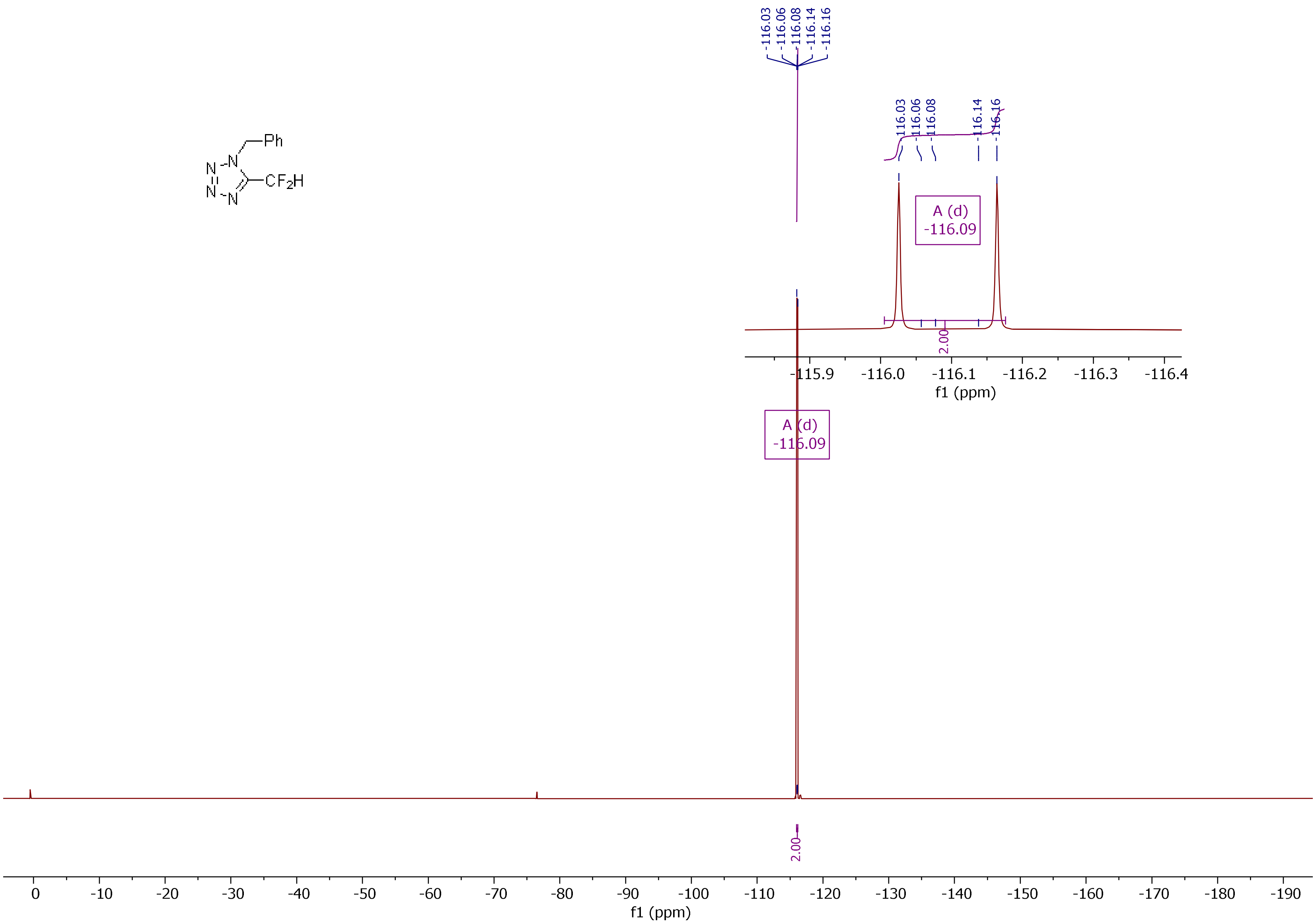

$^{13}C\{^{1}H\}$ NMR spectrum of **5c** ($CDCl_3$, 101 MHz)

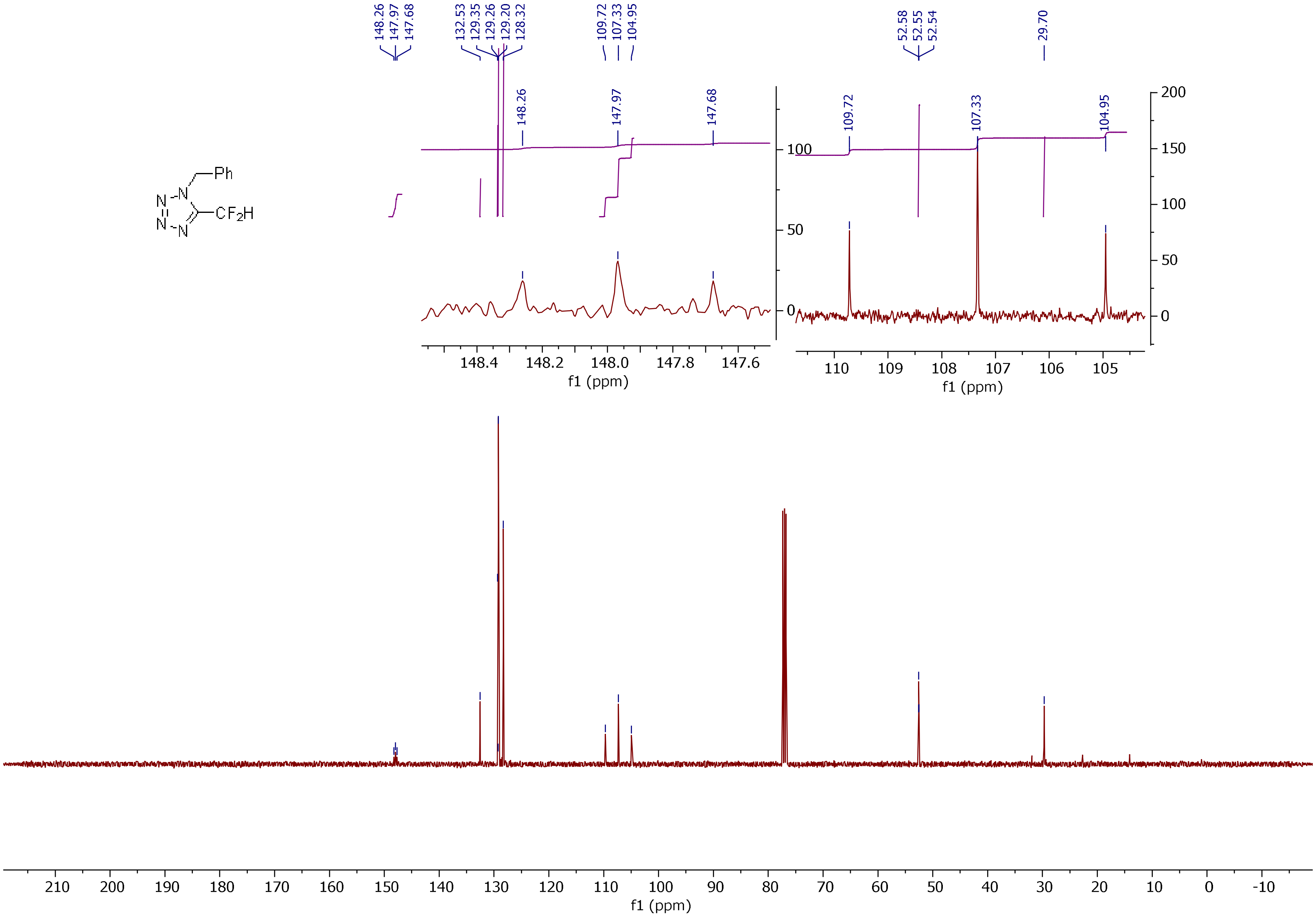

^{1}H NMR spectrum of **5d** ($CDCl_3$, 400 MHz)

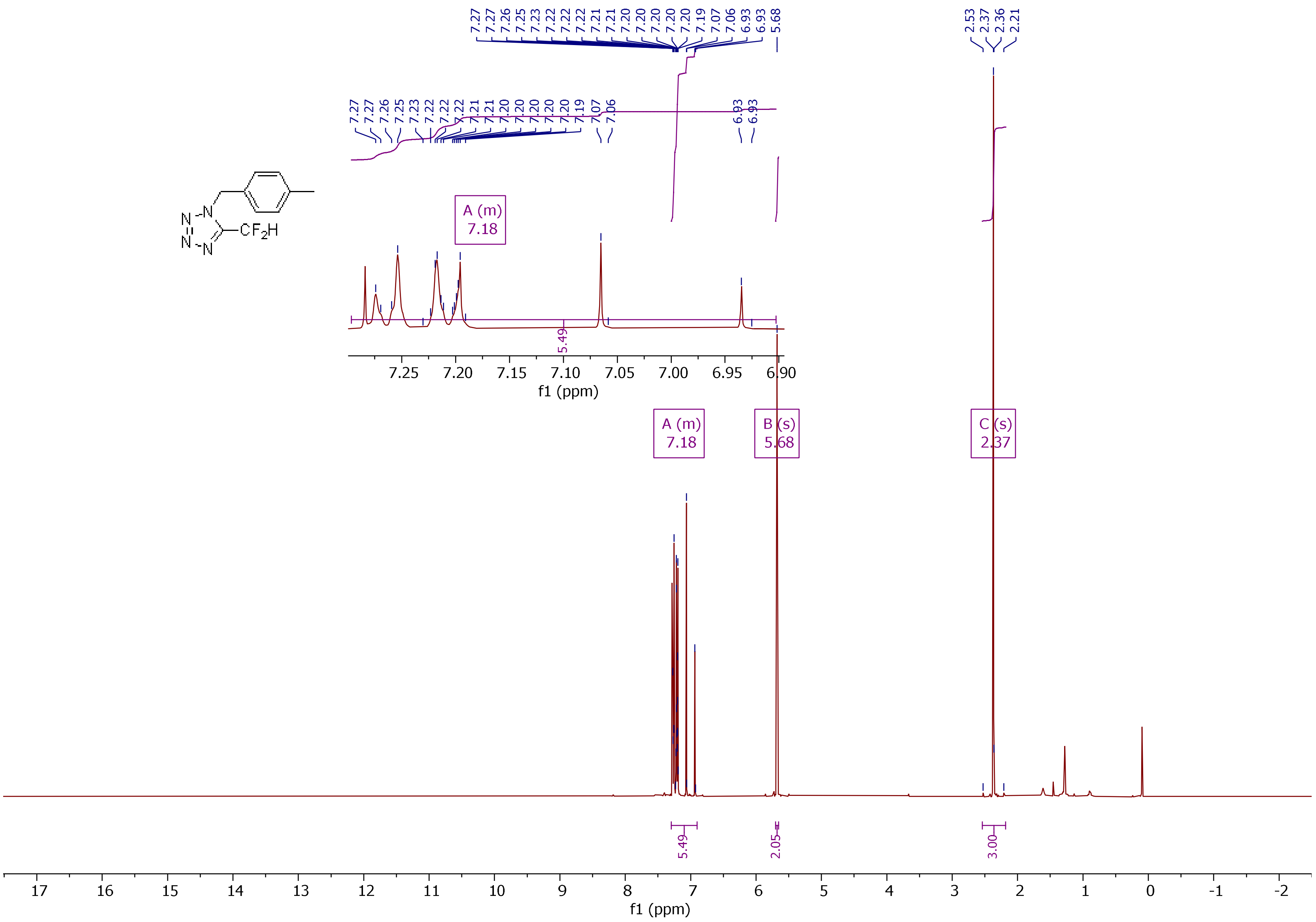

^{19}F NMR spectrum of **5d** ($CDCl_3$, 377 MHz)

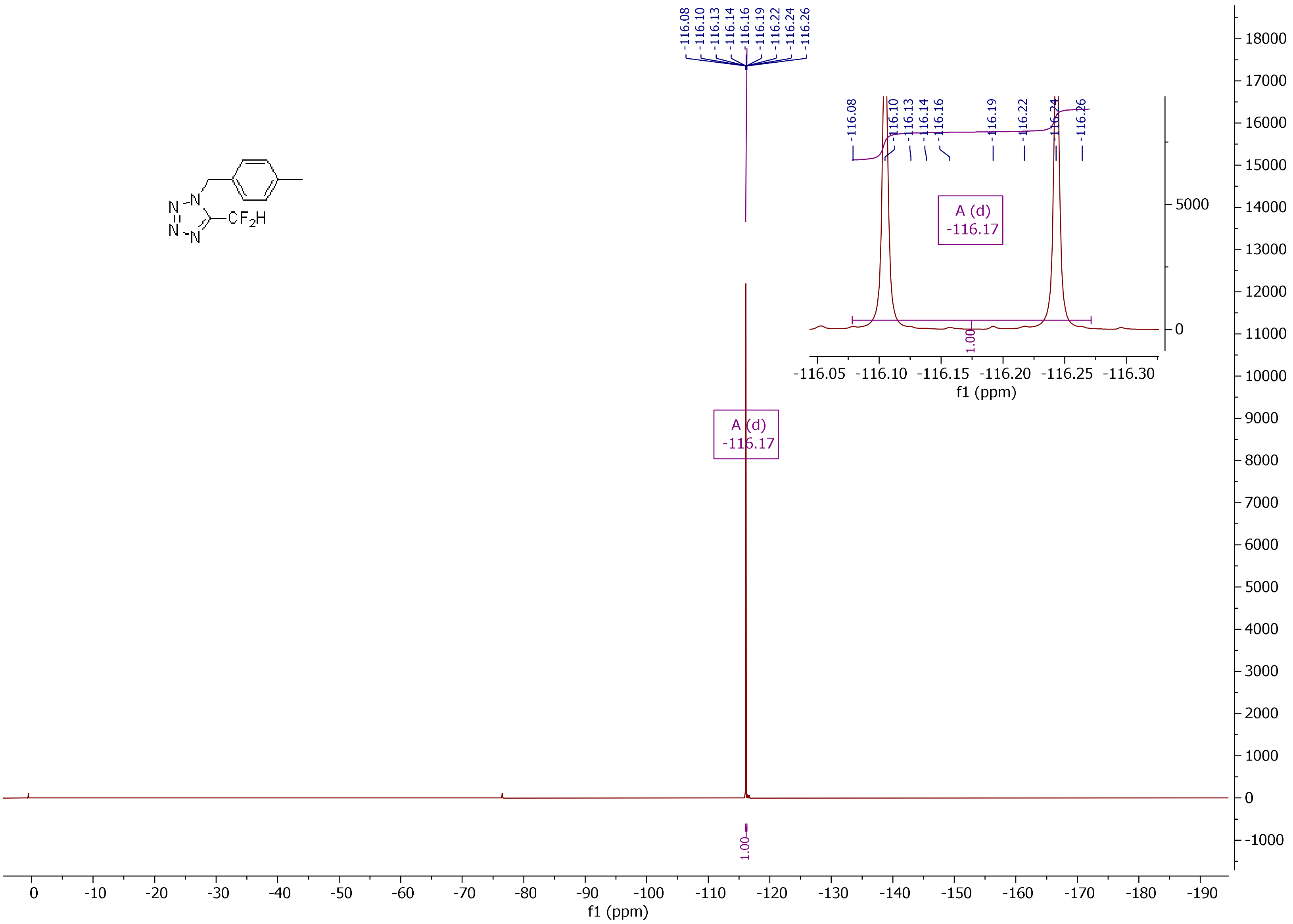

^{13}C{^{1}H} NMR spectrum of **5d** (CDCl$_3$, 101 MH

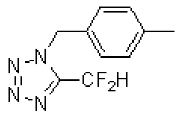

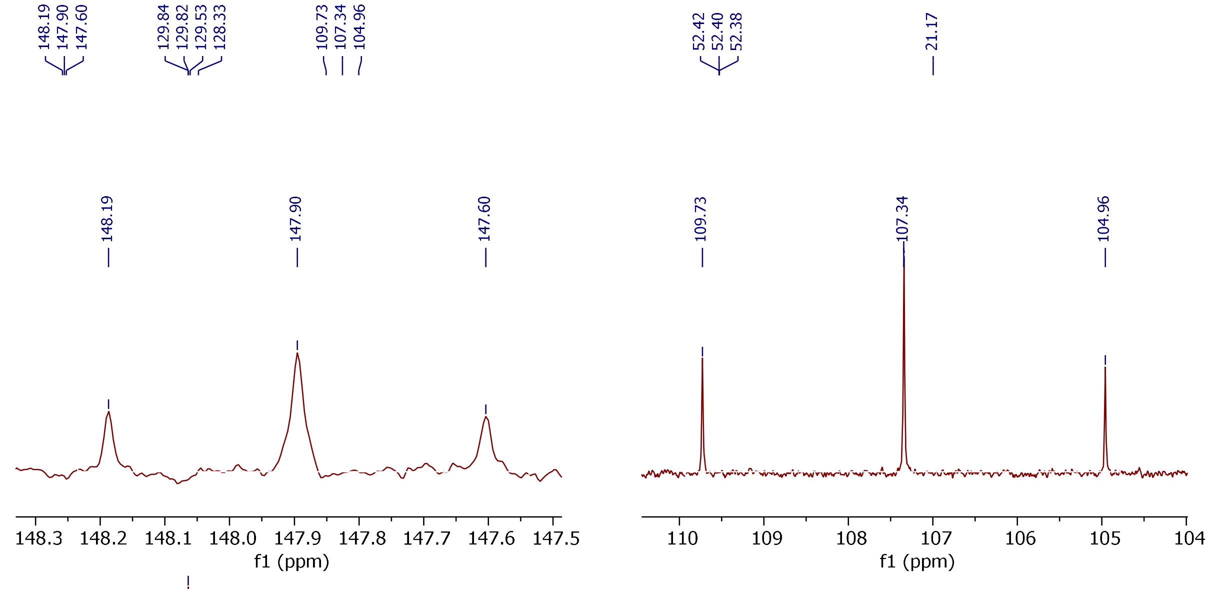

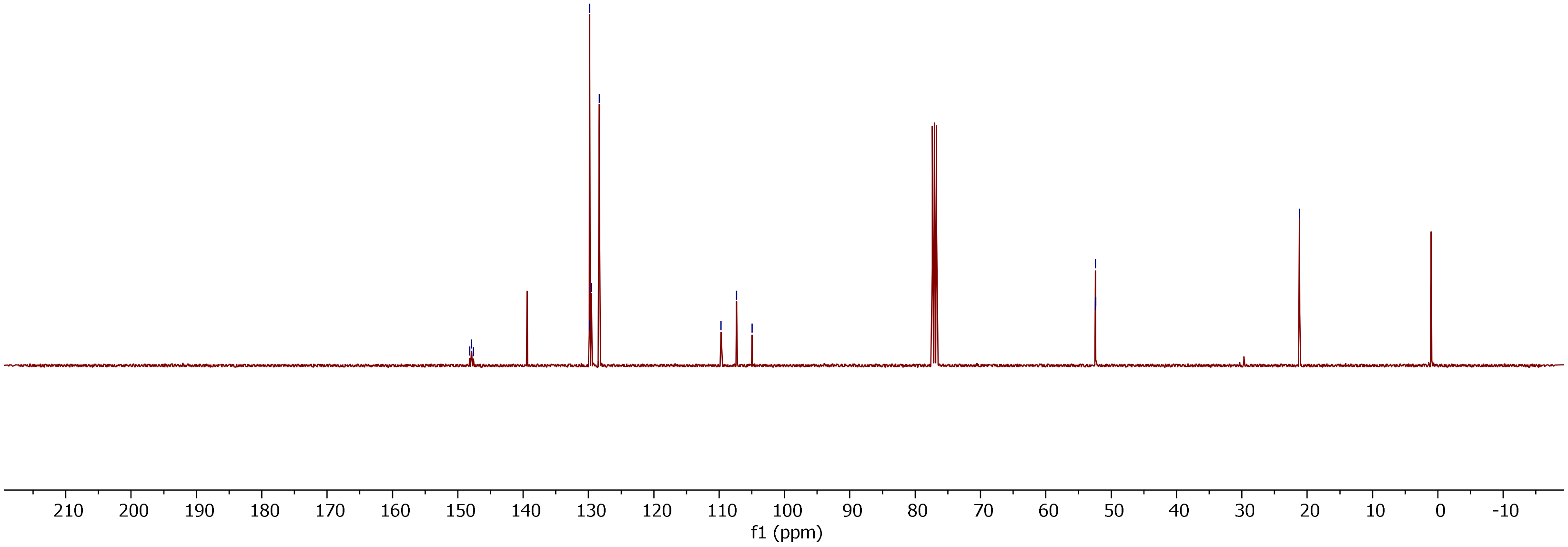

5. References

1 Recommendation on the transport of dangerous goods, Manual of tests and criteria, Union Nations, New York, 2015, pp. 63-68, 85-92.

2 Council Regulation (EC) No. 440/2008, pp. 142/93-142/103.

3 SAINT. Bruker AXS Inc., Madison, Wisconsin, USA, 2015.

4 Altomare, A.; Cascarano, G.; Giacovazzo G.; Guagliardi A.; Burla M. C.; Polidori, G.; Camalli, M. *J. Appl. Cryst.* **1994,** *27*, 435.

5 Betteridge, P. W.; Carruthers, J. R.; Cooper, R. I.; Prout, K., Watkin, D. J. *J. Appl. Cryst.* **2003**, *36*, 1487.
